# Yeast metabolic innovations emerged via expanded metabolic network and gene positive selection

**DOI:** 10.15252/msb.202110427

**Published:** 2021-10-22

**Authors:** Hongzhong Lu, Feiran Li, Le Yuan, Iván Domenzain, Rosemary Yu, Hao Wang, Gang Li, Yu Chen, Boyang Ji, Eduard J Kerkhoven, Jens Nielsen

**Affiliations:** ^1^ Department of Biology and Biological Engineering Chalmers University of Technology Gothenburg Sweden; ^2^ National Bioinformatics Infrastructure Sweden Science for Life Laboratory Chalmers University of Technology Gothenburg Sweden; ^3^ The Novo Nordisk Foundation Center for Biosustainability Technical University of Denmark Lyngby Denmark; ^4^ BioInnovation Institute Copenhagen N Denmark

**Keywords:** genome analysis, genome‐scale metabolic models, metabolic innovation, systems biology, Evolution & Ecology, Metabolism

## Abstract

Yeasts are known to have versatile metabolic traits, while how these metabolic traits have evolved has not been elucidated systematically. We performed integrative evolution analysis to investigate how genomic evolution determines trait generation by reconstructing genome‐scale metabolic models (GEMs) for 332 yeasts. These GEMs could comprehensively characterize trait diversity and predict enzyme functionality, thereby signifying that sequence‐level evolution has shaped reaction networks towards new metabolic functions. Strikingly, using GEMs, we can mechanistically map different evolutionary events, e.g. horizontal gene transfer and gene duplication, onto relevant subpathways to explain metabolic plasticity. This demonstrates that gene family expansion and enzyme promiscuity are prominent mechanisms for metabolic trait gains, while GEM simulations reveal that additional factors, such as gene loss from distant pathways, contribute to trait losses. Furthermore, our analysis could pinpoint to specific genes and pathways that have been under positive selection and relevant for the formulation of complex metabolic traits, i.e. thermotolerance and the Crabtree effect. Our findings illustrate how multidimensional evolution in both metabolic network structure and individual enzymes drives phenotypic variations.

## Graphical abstract

A large‐scale systematic evolution analysis, metabolic model reconstruction and simulation are used to examine the evolutionary mechanism underlying the emergence of diverse traits across 332 different yeast species.

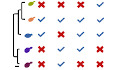

## Introduction

Budding yeasts are unicellular fungi with > 1,000 known species. They have evolved over a period of 400 million years and are widely distributed across different ecosystems (Walker, 2009). These yeast species have numerous traits that are of interest for life science, making them efficient cell factories to produce valuable products (Nielsen, 2019) and model organisms to study human diseases (Poswal & Saini, 2017). Large‐scale whole‐genome sequencing has paved ways towards the understanding of metabolic diversity in different yeast species (Peter *et al*, 2018; Shen *et al*, 2018), for example, by correlating the existence of certain enzyme‐encoding genes with the ability to metabolize a given substrate (Riley *et al*, 2016; Opulente *et al*, 2018). Indeed, connections between genes and metabolic traits in yeast have been mechanistically explored and validated experimentally (Goncalves *et al*, 2020). Moreover, it has been verified that complex traits are always due to the contributions of multiple genes or mutations (Fox *et al*, 2015; Krause *et al*, 2018; preprint: AlZaben *et al*, 2021). Subpathway evolution encompassing the discrete gene evolution events could play a significant role in gain of new functions for yeasts (Wong & Wolfe, 2005; Goncalves & Goncalves, 2019). These studies demonstrate that the evolution from gene to pathways all potentially assure that the strains have the flexibility to gain new capabilities under specific niches.

While it has been shown that yeast metabolic innovation can be attributed to gene duplication and enzyme promiscuity (Hittinger & Carroll, 2007; Opulente *et al*, 2018), it is unknown to what extent these two events have contributed to the evolution at the metabolic network level for gain of functions. Also, besides gene duplication and enzyme promiscuity, there has not been a systematic investigation in yeast for how to connect multiple evolutionary events and characterize general evolutionary trends under the emergence of different metabolic traits. To this end, it is therefore becoming indispensable to apply integrative evolution analysis from gene level, subpathway level to the system level, where the latter can be represented by a metabolic model. Genome‐scale metabolic modelling (GEM) is a computational modelling framework that allows cellular metabolic networks to be examined from a holistic perspective by predicting cellular phenotypes under the disturbance from genotypes and external environments (O'Brien *et al*, 2015). The comparison between predicted cellular traits at large scale with species‐specific GEMs and molecular evolutionary features can help to elucidate evolutionary clues for trait diversity (Seif *et al*, 2020). However, to date GEMs have only been constructed for 12 yeast species (Domenzain *et al*, 2021), limiting their use in elucidating the evolution of metabolic trait diversity among yeasts.

Here, we advance the understanding of the evolutionary mechanisms underlying versatile yeast metabolic traits by combining systematic evolution analysis with metabolic model reconstruction and simulation. We firstly reconstructed a pan‐genome‐scale metabolic model (pan‐GEM), followed by the reconstruction of species‐specific GEMs (ssGEMs) for 332 yeast species and 11 outgroup fungal species, based on detailed gene function annotation and enriched physiological studies. The predictive capabilities of ssGEMs were validated against experimental data. Subsequently, we investigated the main constraints shaping yeast genomic evolution at both gene‐ and residue‐site levels by leveraging the GEM simulation and protein‐structure‐guided function annotation. Lastly, integrative evolution analysis with GEM simulation was used to investigate how various evolutionary events mechanistically resulted in gain and loss of functions in specific lineages. The model prediction and gene selection analyses could identify some consistent gene features relevant for emergence of complex traits (like thermotolerance) in yeast. Together, at a system level, our work highlights that metabolic network expansion through gene duplication and enzyme promiscuity, and divergent sequence evolution (including positive selection) are the main driving forces underlying metabolic innovations in the yeast subphylum.

## Results

### Framework of evolution analysis in connecting genome evolution with trait variance

**Figure 1:**
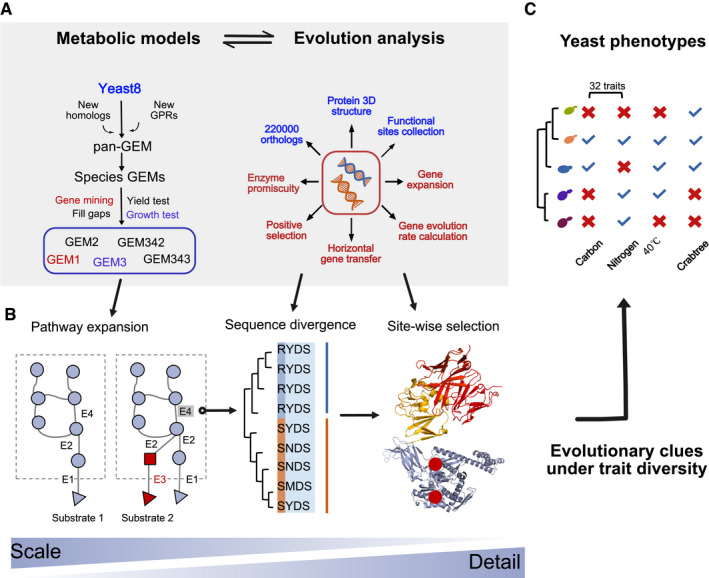
Schematic overview of the framework used in this work to explore how new traits evolved across yeast subphylum by combining systematic evolution analysis with the metabolic network reconstruction and simulation AReconstruction of 343 species‐specific GEMs by leveraging the pan‐GEM expanded from the *S. cerevisiae* GEM Yeast8 (left); and different kinds of sequence‐based evolution analysis conducted in this work (right). During the evolution analysis, the gene ortholog group, protein 3D structure data and functional site annotation (blue part) were used as the basis for other more in‐depth calculations (red part).BPotential mechanisms underlying trait diversity of yeast. Pathway expansion by distinct enzymes could enable novel substrate utilization. Divergent sequence evolution could further change the specialty of enzymes by increasing its activity. Lastly, long‐term site‐wise selection will fix useful mutations to increase the cellular fitness under specific niches.CVarious trait data collected for yeast species, including the ability to grow on 32 carbon and nitrogen sources, and the complex traits of thermotolerance and Crabtree effect. All the trait data could be found in Dataset EV1.

In order to examine the evolutionary origin of observed trait diversity in yeast, a comprehensive evolution analysis was conducted by leveraging GEM reconstruction and simulation (Fig 1A). Starting from the available genomes of 332 yeast species plus 11 outgroup fungal species (Shen *et al*, 2018), we collected detailed traits data for each species (Kurtzman *et al*, 2011; Hagman *et al*, 2013; Hagman & Piskur, 2015; Opulente *et al*, 2018; Shen *et al*, 2018), e.g. information on substrate utilization, presence of the Crabtree effect and heat tolerance (Dataset EV1). This allowed association of genome evolution at multidimensional levels (i.e. subpathway, gene and residue site) to different cellular traits (Fig 1B and C). Meanwhile, we annotated all of the studied genomes in detail (Appendix Fig S1A‐1I), yielding the ideal input for reconstruction of a yeast pan‐GEM and ssGEMs (Fig 1A, Appendix Fig S1J), as well as systematic analyses of fungal genome evolution, such as horizontal gene transfer events, gene family expansion and gene evolution rate estimation (Fig 1B).

### Reconstruction ssGEM for 343 fungal species

**Figure 2:**
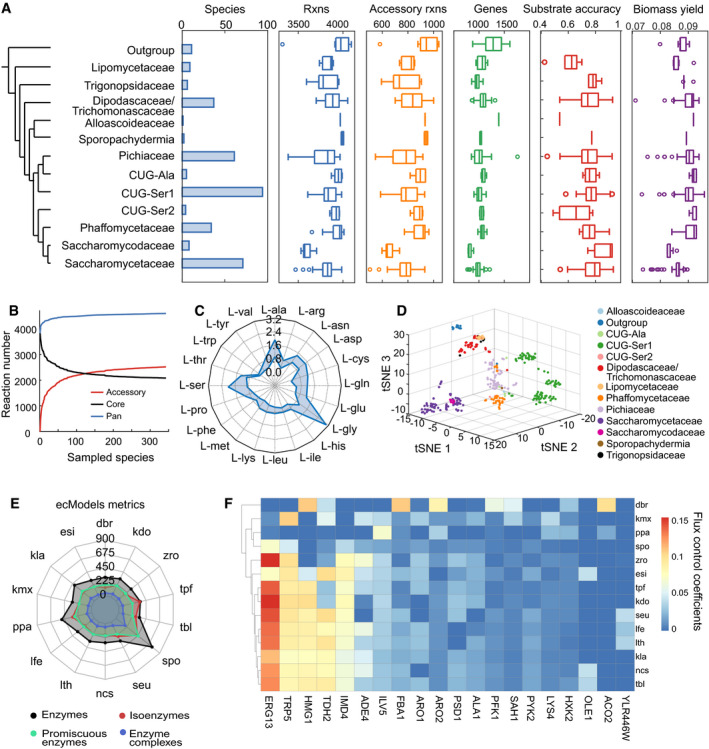
Yeast genomic and metabolic diversity can be reflected by GEM reconstruction, comparison and simulation AMetabolic variance of 332 yeast species from 12 major clades revealed by model reconstruction and simulation. The tips in the phylogenetic tree represent 12 major clades in the subphylum classification for 332 yeast species plus 11 fungal species as outgroup, according to Shen *et al* (2018), and this species classification is used throughout analyses. Substrate accuracy specifies the accuracy for substrate utilization prediction against experimental data (Dataset EV1). Biomass yield was estimated by simulation on minimal media with 1 mmol glucose as input. In each boxplot, the central band and boxes represent the median and interquartile values, respectively, and the whiskers extend up to 1.5 times the interquartile range beyond the box range. During calculation, each group takes the maximal number of yeast species it covers, i.e. larger than three different species except for clade of *Alloascoideaceae* (one species) and clade of *Sporopachydermia* (two species). Each point represents a distinct species. The number of species in each clade for each box plot is shown in the left bar plot. Outliers beyond the whisker are plotted as single points.BProfiles of pan, core (existing in all sampled species) and accessory (existing in part of sampled species) reactions as the numbers of analysed yeast and fungal species increase from 1 to 343.CRanges of *in silico* theoretical maximum production yields of each amino acid across yeast species. The blue zone represents the distribution of predicted maximum yields from all yeast species in this study. The unit for the yield is mol amino acid per mol glucose.Dt‐SNE clustering analysis of yeast species based on the existence of reactions in ssGEMs.ENumber of enzymes within different classes (all, isoenzymes, promiscuous and involved in enzyme complexes) from ecGEM reconstruction for 14 yeast species. The horizontal labels in the radar map represent the number of different kinds of enzymes encompassed in ecGEMs.FHeatmap of flux control coefficients calculated by ecGEMs for 14 yeast species. The *x*‐axis refers to yeast enzymes, while the *y*‐axis indicates yeast species. dbr: *Dekkera bruxellensis*; esi: *Eremothecium sinecaudum*; kla: *Kluyveromyces lactis*; kmx: *Kluyveromyces marxianus*; ppa: *Komagataella pastoris*; lfe: *Lachancea fermentati*; lth: *Lachancea thermotoleran*s; nc*s*: *Naumovozyma castellii*; seu: *Saccharomyces eubayanus*; spo: *Schizosaccharomyces pombe*; tbl: *Tetrapisispora blattae*; tpf: *Tetrapisispora phaffii*; zro: *Zygosaccharomyces rouxii*; kdo: *yHMPu5000034710 Kluyveromyces dobzhanskii*. Source data are available online for this figure.

We firstly reconstructed a yeast pan‐GEM using a new pipeline (Materials and Methods) developed based on the template model strategy (Machado *et al*, 2018; Correia & Mahadevan, 2020) (Fig 1, Materials and Methods). The pan‐GEM comprises of metabolic reactions and enzymes from all 343 fungal species, containing a total of 3,135 metabolites, 4,599 reactions and 3,751 ortholog groups, which therefore represents a significant expansion of coverage in metabolism compared with a prior fungal pan‐GEM (Correia & Mahadevan, 2020) (Appendix Fig S1J). The ssGEMs for all 343 fungal species were initially generated based on the existence of enzyme orthologs as annotated in the pan‐GEM, then followed by gap‐filling (Appendix Fig S1K, Materials and Methods). Overall, each ssGEMs contain 3,500–4,000 reactions, and around 1,000 genes for each (Fig 2A), which are comparable to the previously curated models of eight intensively studied yeast species (Appendix Fig S2A). The reactions from pan‐GEM can be subdivided into 2,080 core and 2,519 accessory ones based on their incidence across all 343 species (Fig 2B, Materials and Methods). The core reactions are found to be more likely involved in central metabolism, while accessory ones are more likely to be involved in degradation pathways and secondary metabolism (Appendix Fig S2B), in agreement with the idea that different accessory reactions may increase the abilities of individual yeast species to adapt to their niche (Opulente *et al*, 2018).

We found that large‐scale ssGEM reconstructions can aid in genome annotations for less‐studied species by characterizing previously unclear substrate utilization pathways. For example, the erythritol degradation pathway has yet not fully elucidated for most yeast species (Carly *et al*, 2018), while two distinct degradation pathways were recorded in the MetaCyc database (Caspi *et al*, 2016) (Appendix Fig S2C). ssGEMs show that erythritol degradation pathway II is likely more widespread in budding yeasts than erythritol degradation pathway I, as no hits are found for two essential reactions of the latter pathway. In 85 yeast species, the genomic evidence for all three enzymes of erythritol degradation pathway II was detected, consistent with experimental evidence that 68 of these species are able to utilize erythritol. In contrast, two key enzymes (EC 2.7.1.215 and 5.1.1.38) in erythritol degradation pathway I are absent across all studied species (Appendix Fig S2C). Of the remaining 17 species with genomic evidence for erythritol degradation pathway II, no experimental data were available for 11 species, while the remaining six species were not able to utilize erythritol based on trait data (Dataset EV1), which may be caused by, e.g. transcriptional regulation and low enzymatic activity.

During ssGEM reconstruction, we were also able to curate the reaction existence and gene association in the reference *S. cerevisiae* model Yeast8 (Lu *et al*, 2019). For example, glucosamine‐6‐phosphate deaminase catalyses alpha‐D‐glucosamine‐6‐phosphate degradation to fructose 6‐phosphate (reaction r_0465 in pan‐GEM, R00765 in KEGG), which is critical for catabolism of N‐acetyl‐D‐glucosamine and its related metabolites. This reaction has been present in *S. cerevisiae* GEMs ever since the first *S. cerevisiae* GEM iFF708 from 2003 (Förster *et al*, 2003), and likewise in the here used Yeast8 model. However, the pan‐GEM‐derived model for this species indicated the absence of this reaction, which is consistent with absence of *in vivo* growth on N‐acetyl‐D‐glucosamine (Flores & Gancedo, 2018). Also, Yeast8 did not have genes associated with fifth step of CoA synthesis from (R)‐pantothenate, while our model construction pipeline annotated the gene YGR277C to this reaction, in consistence with the SGD database annotation (Cherry *et al*, 2012). As a whole, we refined gene associations for 14 reactions and expanded the gene coverage by adding 15 genes to our reference model Yeast8 (Dataset EV2).

### ssGEM simulations can recapitulate metabolic phenotypes and evolutionary relationships between yeast species

Using ssGEMs to simulate species‐specific substrate utilization, the average accuracy of model predictions against experimental data was above 75% (Fig 2A), reflecting the high quality of the ssGEMs that we have constructed. In the cases where there are inconsistencies between model predictions and experimental evidence for substrate utilization (primarily false positives), we assigned cause of inconsistencies to the potential reactions and corresponding enzymes from ssGEMs and observed that these partially arise from uncertainties related to promiscuous enzymes that catalyse multiple reactions in GEMs (Nam *et al*, 2012) (Appendix Fig S2D). As an additional test to benchmark the quality of our ssGEMs, we also used model simulations to predict essential metabolic genes in five species for which there is experimental evidence of gene essentiality (Dataset EV2). The average accuracy in metabolic essential gene prediction using our ssGEMs was over 0.78 for all five species, comparable to that from the reported ssGEMs (Appendix Fig S3A), again showcasing the high quality of the ssGEMs constructed in this study.

For each yeast species, we were able to predict their metabolic flux distributions at maximum growth rate, in minimal medium under aerobic conditions. This allowed the species‐specific biomass, ATP and amino acid yields to be calculated *in silico* (Fig 2A and C, Appendix Fig S2B). The *Saccharomycodaceae* and *Saccharomycetaceae* clades have a lower biomass and ATP yield *in silico* (two‐tailed Wilcoxon rank sum test, *P* value < 0.001, compared with *Phaffomycetaceae* clade), coinciding with the absence of complex I of the electron transfer chain in these yeasts (Appendix Fig S2B), which is consistent with the measured biomass yields for yeast species with and without complex I (Van Hoek *et al*, 1998; Christen & Sauer, 2011; Juergens *et al*, 2020) (Dataset EV2). We then performed the classification of yeast species based on similarity of their corresponding ssGEMs, and found that they clustered largely according to their taxonomic clades (Fig 2D), reflecting that members from the same clade have more similar metabolic network topology structures. However, clusters of certain clades, particularly CUG‐Ser1 (Fig 2D, dark green) and *Phaffomycetaceae* (Fig 2D, dark orange), can be further divided into distinct groups, suggesting that metabolic diversity presented by ssGEMs might enable further classification under these clades.

Previously, we have shown that the predictive performance of traditional GEMs can be improved by incorporating constraints on enzyme concentrations (Sanchez *et al*, 2017). Construction of enzyme‐constrained GEMs (ecGEMs) is, however, dependent on the availability of species‐specific enzyme turnover (*k*_cat_) data, which limits its construction for all species. Here, we were able to build ecGEMs for 14 yeast species with relatively rich *k*_cat_ data using the GECKO toolbox (Sanchez *et al*, 2017) (Fig 2E, Dataset EV2). Given that ecGEMs can predict the metabolic phenotypes of the 14 yeast species with very high accuracy (Appendix Fig S3B), these ecGEMs were used to calculate *in silico* flux control coefficient (Stephanopoulos *et al*, 1998) (FCC, ratio of change in growth rate to enzyme activity) for each enzyme. The result illustrated that, with the exception of ERG13, the FCCs of ortholog enzymes are largely consistent between different yeast species (Fig 2F).

### Gene‐specific evolution rates are related to metabolic function and essentiality

**Figure 3:**
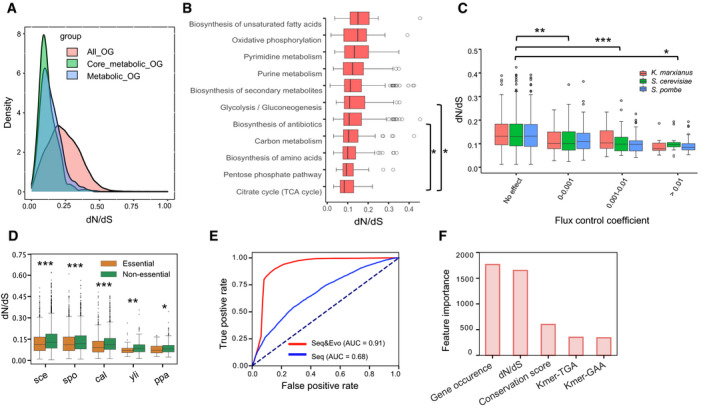
Gene evolution rate is constrained by its protein function in the metabolic network AGene‐specific dN/dS distribution for all OGs, metabolic OGs in pan‐GEM and OGs connected with core reactions across all ssGEMs.BDistribution in gene‐specific dN/dS from the several main typical pathways annotated in ssGEMs.CCorrelation analysis between the average dN/dS and their relevant flux control coefficients as calculated from ecGEMs of three typical yeast species with growth as the objective function.DGene‐specific dN/dS for both essential genes and non‐essential genes across several typical yeast species. sce: *S*. *cerevisiae*, spo: *S*. *pombe*, cal: *C*. *albicans*, yli: *Y*. *lipolytica*, ppa: *K*. *pastoris*.EImproved essential gene prediction on training dataset using support vector machine (SVM) algorithm by adding evolution‐based parameters. The dashed line represents diagonal line with an AUC of 0.5, which means random guessing.FTop features in SVM contributing to the essential gene prediction. Kmer‐TGA and Kmer‐GAA refer to specific 3‐nucleotide sequence fragments, features with lower importance are not shown. Feature importance scores were calculated by the chi‐square test. Data information: The statistical analysis in (B–D) is based on the two‐tailed Wilcoxon rank sum test. *P* value < 0.05 (*), *P* value < 0.01 (**) and *P* value < 0.001 (***). In each boxplot, the central band and boxes represent the median and interquartile dN/dS, respectively, and the whiskers extend up to 1.5 times the interquartile range beyond the box range. During calculation, each group takes the maximal number of ortholog genes it covers, i.e. larger than three for each group. Each point represents a distinct ortholog gene. Outliers beyond the whisker are plotted as single points. Source data are available online for this figure.

We catalogued about 200,000 gene families from the 343 species. Removal of spurious sequences and gene families associated with less than seven species resulted in about 13,000 ortholog groups (OGs) that were subsequently used in the calculation of gene‐specific ratios of non‐synonymous to synonymous nucleotide changes (dN/dS) (Materials and Methods). It displays that for most OGs the dN/dS are far smaller than 1, with a median of around 0.25 (Materials and Methods, Fig 3A), which indicates that non‐synonymous mutations in most genes are detrimental and negatively selected during evolution. The dN/dS of metabolic genes are even lower, with a median of around 0.1, suggesting that mutations in metabolic enzymes are more likely to be detrimental than mutations in other genes, and therefore subject to a higher pressure for negative selection. By mapping gene‐specific dN/dS onto metabolic subpathways, we then found that the evolutionary rates of genes in the TCA cycle are significantly lower than those from other pathways (Fig 3B, two‐tailed Wilcoxon rank sum test, *P* value < 0.05). Notably, based on simulation of the newly reconstructed ecGEMs, we showed that enzymes with high control over the cell growth rate (high FCCs) have significantly lower evolutionary rates, suggesting that these enzymes that play pivotal roles in cell growth are highly conserved in evolution (Fig 3C).

Similarly, we found that the essentiality of genes is also directly linked with their evolution rates as essential genes have significantly lower dN/dS in five yeast species with experimental evidence of gene essentiality (Fig 3D). Meanwhile, the essential genes were also shown to be accompanied by higher protein‐level conservation scores and frequency of incidence across the yeast species examined (Appendix Fig S4B). Based on these observations, we hypothesized that these evolution‐based features could be used to distinguish essential from non‐essential genes in a whole‐genome level for a given species, complementing our capability to predict essential metabolic enzymes using ssGEMs. To test this hypothesis, we evaluated two machine learning models (Appendix Fig S4A, Materials and Methods), support vector machines (SVMs) and random forest (RF), to predict gene essentiality based on sequence features alone, or using sequence features in combination with gene’s evolution‐based features. Our results verified that the gene essentiality prediction could be substantially improved by integrating evolution‐based features (Fig 3E and F, Appendix Fig S4C and D). We then used the SVM pipeline to predict essential genes for all remaining 338 fungal species, and compared the essential metabolic genes predicted by this machine learning approach and those predicted via metabolic modelling using ssGEMs. Here, an overall consistency of 66.9% was achieved (Appendix Fig S4E, Dataset EV3). Together, these predictions provide a valuable resource for further studies in the yeast research community.

### Positive selection at the amino acid level is related to both function and localization on the protein 3D structure

**Figure 4:**
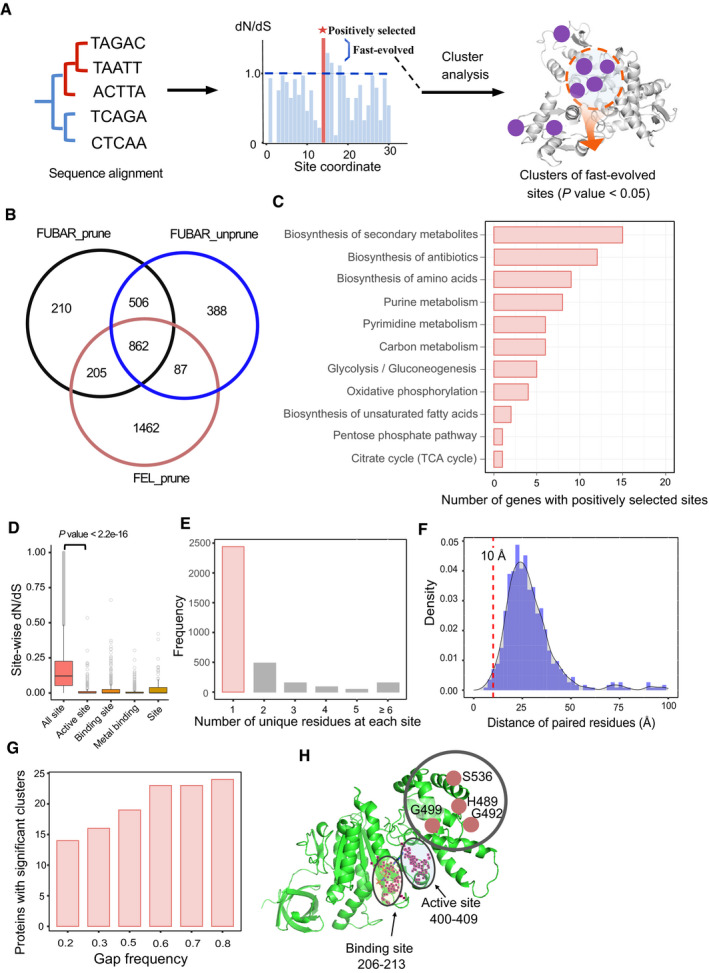
Evolution analysis in codon (amino acid site) level based on protein 3D structures ASchematic pipeline to calculate the site‐wise evolution rate and map the fast‐evolved or positively selected sites onto the protein 3D structures, to enable the mutation cluster analysis.BNumber of genes with positively selected sites across 343 fungal species as calculated by site model using three different methodologies (Materials and Methods). The “prune” indicates whether paralog genes were automatically removed from gene families, to yield just a single gene copy for each yeast species.CDistribution of genes with positively selected sites in different metabolic subpathways. Results for more pathways are shown in Appendix Fig S5.DSite‐wise dN/dS for each functional category of residue sites defined in the UniProt database (The UniProt Consortium, 2017). For comparison, only dN/dS between 0 and 1 are plotted here. Results for more functional categories are shown in Appendix Fig S5. *P* value is from two‐tailed Wilcoxon rank sum test. In each boxplot, the central band and boxes represent the median and interquartile dN/dS, respectively, and the whiskers extend up to 1.5 times the interquartile range beyond the box range. During calculation, each group takes the maximal number of residue sites it covers; i.e., at least 199 residues site from group of “Site” and the number is much larger in other groups. Each point represents a distinct residue site. Outliers beyond the whisker are plotted as single points.EStatistical analysis in count of unique amino acid residues at each functionally important site (including active site, binding site, metal binding site and site according to UniProt database) across 343 fungal species.FDensity plot of spatial distances between the highly conserved sites (e.g. active sites, binding sites) and the fast‐evolved sites (including the positively selected sites), across 343 fungal species. The dashed line represents adjacent distance at 10 Å.GEffects of the cut‐off in gap ratio during the multiple sequence alignment on the number of proteins detected with significant clusters (*P* value < 0.05) consisting of the fast‐evolved sites (dN/dS > 1).HExample of CLUMPS analysis to obtain the significant clusters consisting of fast‐evolved sites based on protein 3D structures. Shown is subunit alpha of F1F0‐ATP synthase, coded by gene YBL099W and part of ortholog group OG1533. The homology 3D structure data for YBL099W were downloaded from the SWISS‐model database (Waterhouse *et al*, 2018). The coordinate of binding site is from the UniProt database (The UniProt Consortium, 2017) and the coordinate of active site is from the SGD database (Cherry *et al*, 2012), respectively. Fast‐evolved sites at positions 489, 492, 499 and 536 are away from the conserved binding and active sites. Source data are available online for this figure.

While mutations in most OGs are negatively selected at the gene level, we nevertheless screened 862 OGs of high possibility with at least one positively selected site (or amino acid residue, PSS) (Fig 4A and B, Appendix Fig S5A, posterior probability > 0.9) and they are also accompanied by higher evolution rates compared with the remaining OGs (Appendix Fig S5B). Mapping these OGs with PSSs onto metabolic pathways annotated based on KEGG database, the genes with PSSs were mainly detected in the MAPK signalling pathway, Biosynthesis of secondary metabolites and Ribosome (Appendix Fig S5C), among which it is significantly enriched in “Ribosome” in hypergeometric test (*P* value = 0.0017). We also found part of genes from some core metabolic pathways, including central carbon and nitrogen metabolic pathways (Fig 4C and Appendix Fig S5C), consistent with previous reports in primate evolution (Daub *et al*, 2017), and hinting that this pattern of site‐wise positive selection occurs across a wide range of evolutionary domains.

At the amino acid residue level, a wide range of site‐wise dN/dS from 0 to > 3.0 was observed, indicating that diverse selection pressures may be acting on mutations of specific amino acid residues (Appendix Fig S5D). To examine this, we classified each amino acid residue into 32 functional categories (Appendix Fig S5E) based on the functional annotation of the *S*. *cerevisiae* S288c proteome. Notably, the functional categories of particular importance to metabolic enzymes, e.g. active sites and binding sites, have significantly lower dN/dS compared with other functional categories, signifying that the catalytic capabilities of enzymes are highly conserved in evolution (Fig 4D). Focusing on the evolutionary trend at these important sites, we found that most of them are extremely conserved as 2,440 of 3,370 residue sites are kept the same across species in 1,364 proteins with reference from *S*. *cerevisiae* (Fig 4E). For example, all five functionally important sites (H9, R60, E87, K98 and H182) of phosphoglycerate mutase 1 (YKL152C) are invariable across all yeast species. Also, we could find signs of divergent evolution at some important sites; for example, for the putative 6‐phosphofructo‐2‐kinase (YLR345W) in 337 fungal species, the active site at D173 in 23 species mainly from *Metschnikowia* genus was substituted by glutamic acid, while the reference and alternative residues are both belong to ionic amino acids.

We then mapped the PSSs and fast‐evolved sites (site‐wise dN/dS > 1) onto about 3,700 reference protein 3D structures from *S*. *cerevisiae* S288c (Fig 4A, Appendix Fig S6, Materials and Methods), to examine the spatial distribution of these sites. As a whole, it exhibits that positively selected and/or fast‐evolved sites are spatially distant from functionally important sites (i.e. active sites and binding sites) as calculated based on protein 3D structures, with most fast‐evolved sites being > 10 angstrom away from protein active sites or binding sites (Fig 4F). These positively selected and/or fast‐evolved sites also tend to be spatially diffuse, as most of them do not form clusters when mapped onto protein 3D structures. Significant clusters encompassing the above sites with gap frequency cut‐off of ≤ 0.3 were detected in only 16 proteins (Fig 4G, *P* value < 0.05), several of which are relevant to the cellular stress response (Dataset EV4). The spatial distribution of these significant clusters on the protein 3D structures is again found to be distant from active sites and binding sites, as exemplified by 3D structure of YBL099W (Fig 4H). Taken together, these results confirm that amino acid residues in metabolic enzymes are subject to different selection pressures depending on both their function and localization in the protein 3D structures (Echave *et al*, 2016).

### Evolutionary mechanisms underlying substrate utilization diversity in yeast

**Figure 5:**
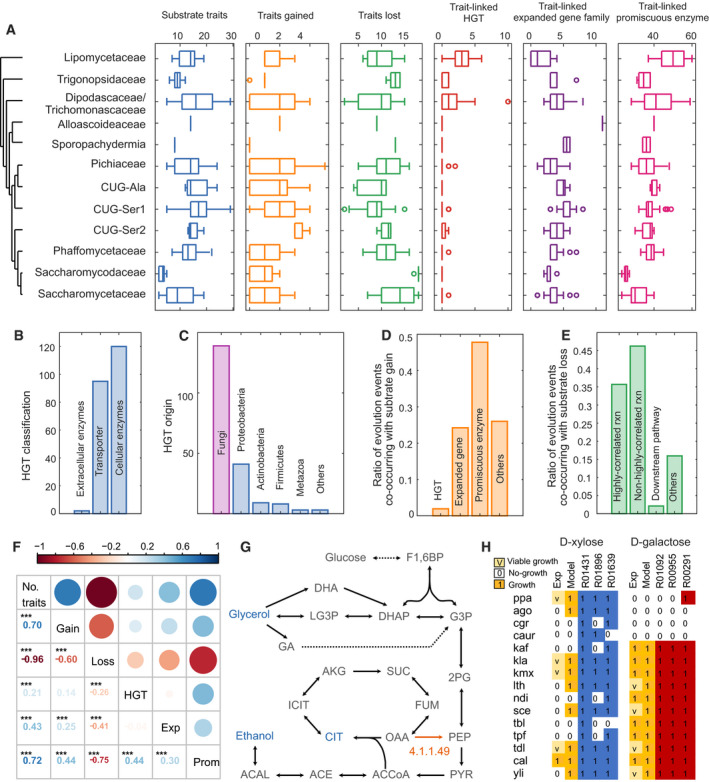
Probing evolutionary mechanisms underlying the trait diversity in substrate utilization for 332 yeast species, through model simulation and systematic evolution analysis ANumbers of traits, gain and loss of function in substrate utilization, in relation to the budding yeast common ancestor (BYCA), in addition to numbers of horizontal gene transfer (HGT) events, expansions of existing gene families and promiscuous enzymes, all related to substrate utilization traits across 12 main clades of the yeast subphylum. In each boxplot, the central band and boxes represent the median and interquartile values, respectively, and the whiskers extend up to 1.5 times the interquartile range beyond the box range. During calculation, each group takes the maximal number of yeast species it covers, i.e. larger than three different species except for clade of *Alloascoideaceae* (one species) and clade of *Sporopachydermia* (two species). Each point represents a distinct species. Outliers beyond the whisker are plotted as single points.BClassification of genes gained through HGT related to substrate utilization based on their function and compartmental annotation.COrigin of HGT genes (i.e. donor organisms) as identified from 332 yeast species.DRatios of different evolutionary events related to gain of function in substrate utilization.ERatios of evolutionary events related to loss of function in substrate utilization. Downstream pathway is defined here as cases where all enzymes and reactions from the direct substrate utilization pathway are present in the organism, but specific reactions in distantly related pathway (i.e. “downstream pathway”) are absent, thereby preventing successful substrate utilization.FCorrelation analysis among number of total traits (No. of traits), gain of traits (Gain), loss of traits (Loss), HGT events (HGT), expanded gene families (Exp) and promiscuous enzyme (Prom). *** means *P* value < 0.001 in the correlation *t*‐test analysis.GExample of a loss of substrate utilization trait caused by a defect in a downstream pathway. The reaction catalysed by EC4.1.1.49 (phosphoenolpyruvate carboxykinase, in orange) is essential to utilization of ethanol and citrate as substrates, even though it is not directly involved in their degradation. Meanwhile, utilization of glycerol would be unaffected by the absence of EC4.1.1.49. All potential substrates are shown in blue, dotted arrows indicate multiple reaction steps, and arrowhead indicates reaction direction and reversibility.HExample of highly correlated and non‐highly correlated reactions, defined as those reaction that do, or do not, exist together with the substrate utilization phenotype. Highly correlated reactions are defined as consistency > 0.83, sensitivity > 0.92 (Materials and Methods). Reactions R01431, R01896 and R01639 are catalysed by D‐xylose reductase, D‐xylulose reductase and xylulokinase, respectively, while these enzymes might also be involved in utilization of other sugars, e.g. arabinose. The presence of these reactions in the model does not correlate well with the xylose utilization phenotype and is not highly correlated. While *S*. *cerevisiae* has all three genes, it cannot grow on xylose. Meanwhile, galactose utilization is highly correlated to reactions R01092, R00955 and R00291, catalysed by galactokinase, galactose‐1‐phosphate uridylyltransferase and UDP‐glucose 4‐epimerase, respectively. Reaction identifiers are from KEGG. Exp stands for the experimental phenotype, and Model stands for model simulated phenotype. ppa: *Komagataella pastoris*; ago: *Eremothecium gossypii*; cgr: *Candida glabrata;* caur: *Candida auris;* kaf: *Kazachstania africana;* kla: *Kluyveromyces lactis*; kmx: *Kluyveromyces marxianus*; lth: *Lachancea thermotoleran*s; ndi: Naumovozyma dairenensis; sce: *Saccharomyces cerevisiae;* tbl: *Tetrapisispora blattae*; tpf: *Tetrapisispora phaffii*; tdl: *Torulaspora delbrueckii*; cal: *Candida albicans*; yli: *Yarrowia lipolytica*. Source data are available online for this figure.

As ssGEMs were able to associate metabolic traits with genomic and site‐specific diversity, we could further leverage these models to investigate how the trait diversity of yeast has arisen, by mapping different evolutionary events onto catabolic metabolic pathways from ssGEMs. Firstly, we compared the substrate utilization of each species to the inferred traits of the budding yeast common ancestor (BYCA) (Shen *et al*, 2018), and catalogued the gains and losses of these metabolic traits (Fig 5A). We next conducted more detailed evolution analysis at gene levels, i.e. gene family expansion (contraction) and horizontal gene transfer (HGT) analyses. Then, for each change in substrate utilization relative to the BYCA, we determined whether this was brought about by expansion of an existing gene family (Fig 5A, Appendix Fig S7A and B), HGT (Fig 5B and C, Appendix Fig S7C, Dataset EV5) or a promiscuous enzyme that changed its substrate specificity (Materials and Methods). The data suggested that HGT contributes relatively little to trait gains or losses (Fig 5A); however, many genes evolving from HGT events were transporters or extracellular substrate degradation enzymes (Fig 5B), which likely plays a role in enlarging the substrate utility of yeast. The dominant source of HGT is from other fungi (Fig 5C) suggesting a frequent gene flow among the fungal species. There is obvious variability in HGT events related to substrate degradation among various clades (Fig 5A). For the *Wickerhamiella*/*Starmerella* (W/S clade) and its close relatives, e.g. *Lipomycetaceae*, *Trigonopsidaceae*, *Dipodascaceae*/ *Trichomonascaceae*, there are more HGT events due to the fact that the large majority of species in these clades are ecologically associated with other fungal species or eukaryotes (Goncalves *et al*, 2020), while there are very few or zero HGT events related to substrate degradation in the CUG group and its relatives (e.g. CUG‐Ser1, CUG‐Ser2, *Phaffomycetaceae*), supporting that genetic code alteration can act as a barrier to HGT (Richards *et al*, 2011).

For trait gains, expansion of existing gene families and increased substrate specificity of promiscuous enzymes appear to be the main driving factors (Fig 5D and F). It initially hints that the *Saccharomycetaceae* clade has a higher number of expanded genes and promiscuous enzymes, which can be associated with a wider substrate utilization profile compared with the *Saccharomycodaceae* clade. The duplications of promiscuous enzymes following divergent sequence evolution are frequently observed in yeast. As one typical example, maltase is able to degrade maltose, turanose, maltotriose and sucrose (Brown *et al*, 2010). We found that the responsible gene had at least one duplication in 77 yeast species, among which 74 could utilize maltose and 69 utilize sucrose (Dataset EV6). With the enzyme functional annotation from *S*. *cerevisiae* S288c, it shows that three key residue sites (active site: D214, E276; site: D349) of maltase are highly conserved in each duplication, maintaining the same in about 96% of all ortholog gene members. In some species, divergent sequence evolution at key residue sites can still be observed. For instance, in *Nadsonia fulvescens var. elongata*, one of these key residue sites was mutated in one duplication (from D349 to E349) while remaining unchanged in another duplication. Such a divergent sequence evolution in promiscuous enzyme may determine the specificity of enzyme in catalysing various substrates. More interestingly, in six yeast species with only one duplication of maltase, gaps were found at these three key residue sites, coincidently with the trait loss in maltose utilization in these species (Dataset EV6).

We further simulated substrate utilization using ssGEMs to examine these trait loss events at a holistic level. In simulations, we firstly defined those reactions that always coexist with specific traits as highly correlated reactions (consistency > 0.83, sensitivity > 0.92) (Fig 5H, Materials and Methods). Such highly correlated reactions could be identified for the utilization of 14 substrates out of 32 substrates tested, and loss of these highly correlated reactions plays a large role in trait loss (Fig 5E). The random loss of non‐highly correlated reactions has a similar effect, which indicates that loss of metabolic traits is not always linked with loss of the same reactions in different yeast species. Our model simulations also identified a small number of cases where, although all enzymes and reactions are present in the specific pathway responsible for substrate assimilation, an enzyme/reaction in a distantly related pathway (here defined as “downstream pathway”) is lost, resulting in loss of substrate utilization (Fig 5E). In Fig 5G, we present ethanol utilization as an example: several species in the genus *Hanseniaspora* seemingly contain all enzymes necessary for ethanol utilization (from ethanol to acetyl‐CoA to oxaloacetate; Fig 5G), yet these species cannot catabolize ethanol. We propose that this arises from a missing reaction in gluconeogenesis, which converts oxaloacetate to phosphoenolpyruvate, catalysed by the enzyme oxaloacetate carboxylase (EC4.1.1.49), which is absent in these specific *Hanseniaspora* species (Fig 5G, orange arrow and text). For the same reason, the model simulations show that these species are not able to utilize succinate or citrate as substrates, while ability to utilize glycerol utilization is unaffected (Fig 5G). These *in silico* predictions are in good agreement with the experimentally determined substrate utilization capabilities (Dataset EV1) (Kurtzman *et al*, 2011).

### Evolution of complex phenotypes, Crabtree effect and thermotolerance

While substrate utilization is often a relatively simple trait that can readily be understood from evolution of the required catabolic pathways, it is more challenging to elucidate the evolutionary mechanisms underlying complex traits such as the Crabtree effect and thermotolerance (Caspeta *et al*, 2014) in yeast. Here, integrative analysis from metabolic simulation and gene selection analysis were conducted to find consistent clues underlying the emergence of complex traits in yeast. In our gene selection analysis, the so‐called “branch‐site” model that can deduce whether the positive selection happened on a proportion of species or branches with specific traits (Appendix Fig S5A, Materials and Methods) was used.

**Figure 6:**
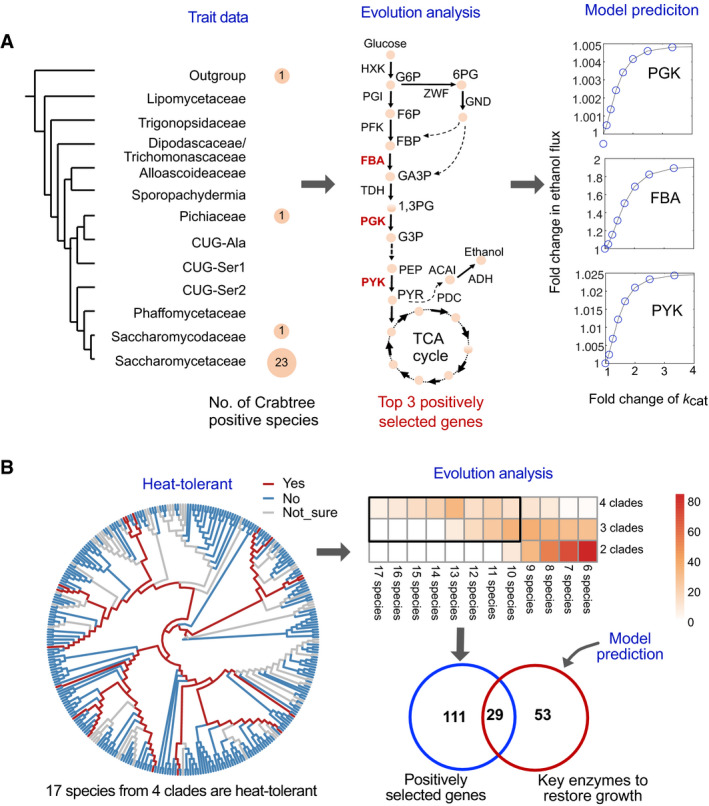
Mechanisms underlying the formation of complex traits revealed by integrative evolution with aid of metabolic simulations AEvolution mechanisms of the Crabtree effect based on the integrated evolution analysis and model simulation. Crabtree is distributed across clades, but dominant in *Saccharomycetaceae* (left). Three top positively selected genes (middle), fructose‐bisphosphate aldolase (FBA), phosphoglycerate kinase (PGK) and pyruvate kinase (PYK) were identified from the intersection of two independent calculations using the “branch‐site” model. Potential correlation between the increased *k*_cat_ of enzymes encoded by the three selected genes and ethanol secretion was evaluated based on a reference metabolic model from Chen and Nielsen (2019) (right). Abbreviations: G6P, glucose 6‐phosphate; 6PG, 6‐phosphogluconate; F6P, fructose 6‐phosphate; FBP, fructose‐1,6‐bisphosphate; GA3P, glyceraldehyde‐3‐phosphate; 1,3PG, 1,3‐bisphosphoglycerate; G3P, glycerate 3‐phosphate; PEP, phosphoenolpyruvate; PYR, pyruvate; ACAI, acetaldehyde; TCA cycle, tricarboxylic acid cycle.BGlobal cellular response to heat tolerance revealed by “branch‐site” model analysis and metabolic model simulation. The detailed phylogenetic tree represents the distribution of thermotolerant species. The heatmap displays the number of positively selected ortholog genes when using different number of clades and species as the cut‐offs (Materials and Methods). The top positively selected genes in the blue circle are the intersection of two independent calculations using the “branch‐site” model. The key enzymes in the red circle that could restore the cellular normal growth were obtained from Li *et al* (2021). Source data are available online for this figure.

As first case of complex traits, we examined the Crabtree effect to test our procedure. Here, we found that there is a combined positive selection for three genes in the EMP pathway (FBA1, PGK, PYK, *P* value < 0.05), and one gene in oxidative phosphorylation (ATP1, *P* value < 0.05), in four independent lineages of Crabtree‐positive yeasts with and without whole‐genome duplication (WGD) having the Crabtree effect (Fig 6A). The positive selection of PGK and PYK in Crabtree‐positive yeast species has been reported in two *Dekkera* yeasts (Guo *et al*, 2016) without WGD by comparing them with five closely Crabtree‐negative species, increasing the confidence in the accuracy of our analysis. Interestingly, it has recently been reported that increased PYK activity through one missense mutant shifts *S. pombe* from respiration towards fermentation (Kamrad *et al*, 2020), while attenuation of PYK activity was important for *S. cerevisiae* to acquire a Crabtree‐negative phenotype (Yu, Zhou, *et al*, 2018). Additionally, simulations of a simplified core metabolic model (Chen & Nielsen, 2019) and the ecGEM of *S. cerevisiae* both allude that increased efficiencies of FBA1, PGK and PYK potentially play a role in the redistribution of fluxes towards fermentation (Conant & Wolfe, 2007); thus, the divergent sequence evolution in these genes may be relevant for the Crabtree effect in some yeast species (Fig 6A, Appendix Fig S8). By comparison, we found no evidence of positive selection for any transcriptional factors (TFs) from these four independent lineages of Crabtree‐positive species.

As the second example, the polygenetic and multiscale features relevant for thermotolerance formation were explored in a similar way. In our work, thermotolerance is mainly observed in four independent clades: *Dipodascaceae/Trichomonascaceae*; *Pichiaceae*; CUG‐Ser1; and *Saccharomycetaceae* (Dataset EV1), although the distribution across species within these four clades is not uniform (Fig 6B). We identified 141 genes to be positively selected in relation to thermotolerance, occurring in at least three independent clades and at least 10 species in each clade (*P* value < 0.05) (Fig 6B). The 141 genes are more conserved across yeast species among the tested OGs, and 140 genes have orthologs in *S*. *cerevisia*e (Appendix Fig S9A). Pathway enrichment analysis shows these genes are enriched in several cellular processes that have previously been shown to contribute to heat tolerance (Puig‐Castellvi *et al*, 2018; Muhlhofer *et al*, 2019), including fatty acid synthesis (*P* value = 0.011), biosynthesis of amino acids (*P* value < 0.0001) and TCA cycle (*P* value < 0.0001) (Appendix Fig S9B, Dataset EV7). These genes were also significantly enriched for GO terms including “translation” (*P* value < 0.0001), “ribosome” (*P* value = 0.019) and “protein folding” (*P* value = 0.028). Furthermore, 42 of the positively selected genes were differentially expressed (up‐ or downregulated, *P* value < 0.05) when comparing their protein levels in *S*. *cerevisia*e cultivated at 30°C and 38°C in chemostat experiments (Lahtvee *et al*, 2017) (Appendix Fig S9C). More importantly, a recent experiment by transferring genes from a thermotolerant yeast species—*Ogataea polymorpha—*to *S. cerevisiae* verified that numerous genes (about 60 genes were tested) can contribute to thermotolerance of yeast and these genes are significantly enriched in GO term of “translation” and “ribosome” (Seike *et al*, 2021), partially consistent with the result here.

Additionally, we identified 35 specific mutations from the above 22 positively selected gene that mainly exist in thermotolerant species (Appendix Fig S9D, Materials and Methods). Mapping these mutations onto reference protein 3D structures revealed that the largest proportion of the mutations occurs in alpha‐helices and interface of 3D structures (Appendix Fig S9D), which might be a kind of convergent evolution for growth at high temperatures. As an example, most thermotolerant yeast species contain two mutations in the protein disulphide isomerase (PDI1, YCL043C), at positions 351 and 355; both are confined by alpha‐helices of the thioredoxin‐like fold of the protein near the active site. However, the functionalities of these mutations and their correlations with thermotolerance need more in‐depth molecular studies.

We further examined the role of enzymes in thermotolerance using the *S. cerevisiae* metabolic model etcYeast7.6, which includes the optimum and melting temperatures for each enzyme (Li *et al*, 2021). Simulations with this model revealed that, at a prohibitory growth temperature of > 40°C, growth can be restored by modifying a combination of 82 enzymes, indicating that the optimum or melting temperatures of these enzymes are not optimized for thermotolerance in *S. cerevisiae*. Of those 82 enzymes, 29 were identified as positively selected in thermotolerant yeast species in our evolution analysis (Fig 6B), further lending confidence to our analyses. These enzymes are significantly enriched in aminoacyl‐tRNA biosynthesis (*P* value = 0.013), biosynthesis of amino acids (*P* value < 0.0001) and EMP pathway (*P* value = 0.0057).

## Discussion

Cellular fitness originates from the integrative effect of multidimensional evolution and optimization (Conrad *et al*, 2010), while the metabolic network structure and the prediction from genome‐scale metabolic models can recapitulate the evolutionary relationships between different species in a system level. Here, for the first time, we reconstructed the ssGEMs for 332 yeast species in large scale (Fig 1), which could systematically characterize the evolution of diverse metabolic traits in yeast (Fig 2). It illustrates that the iterative update in yeast ssGEM could facilitate genome annotations and identify previously unclear metabolic pathways for specific substrate utilization in several species (Appendix Fig S2C), which thus provides a solid basis to explore the multiscale evolution of metabolic traits in the yeast subphylum. Further, the evolution rate calculations at both gene‐ and residue‐site levels reveal that the species from yeast subphylum exhibit negative selection on the gene level (Fig 3A), whereas the positive selection can occur on specific residue sites to shape the enzyme evolution. Combining systems‐level metabolic simulations and gene‐level evolution rate calculations, we discover that the evolution rates of enzymes are modulated by both metabolic function and gene essentiality. Interestingly, the 3D protein structure mapping shows that these positively and fast‐evolving amino acid residues are spatially distant from functionally important sites such as active sites or substrate binding sites within an enzyme (Fig 4F). These findings demonstrate that though the evolution is multidimensional and dynamic, the metabolic functions reflected by ssGEMs and protein 3D structures still potentially shape the evolution at both gene and amino acid residue level.

Metabolic model simulation could enhance evolution analysis in delineating the evolutionary mechanisms underlying the metabolic trait diversity. As for the substrate utilization by yeast, previous studies of small numbers of yeast species have implicated gene duplication events, enzyme promiscuity and/or potential HGT events (Goncalves *et al*, 2018; Milner *et al*, 2019) in the evolution of fungal metabolic diversity. Here, using the largest number of yeast metabolic networks to date, our findings suggest that metabolic trait gains are primarily driven by gene family expansions and enzyme promiscuity (Fig 5F). This indicates an inherent flexibility in yeast metabolism, which has allowed yeast species to evolve new traits to adapt to changes in their niche. Our findings also indicate that HGT events have contributed in only a few cases to expand substrate utilization in certain yeast species (Fig 5D), which is in accordance with some previous studies that HGT has a low frequency in budding yeasts (Marcet‐Houben & Gabaldón, 2010; Goncalves *et al*, 2018; Shen *et al*, 2018). This is in direct contrast to studies in *E. coli*, where HGT was found to be the major event driving metabolic innovations (Pang & Lercher, 2019). Nonetheless, the transporters gained from HGT events in certain yeast species could leverage existing metabolic networks to expand the utilization of additional substrates. Consistent with reductive evolution in genome, loss of function is another decisive factor to determine trait diversity of yeast species, which is highly coupled with the loss in crucial reactions (Fig 5E). On the contrary, metabolic trait loss can be also due to the internal structure of metabolic networks including gaps in downstream pathways in specific yeast lineages (Fig 5G). To the best of our knowledge, this is the first work to investigate the yeast metabolic innovations in large scale via combining genome‐scale metabolic models (GEMs) and evolution analysis. Further studies are needed to fully explore the detailed mechanisms by which reductive evolution has shaped the evolution of metabolic traits in yeast.

Besides substrate utilizations in yeast, we used an integrative evolution analysis with metabolic model simulation to provide a holistic examination of the evolution of complex traits. Our result hints that the emergence of the Crabtree effect seems to be accompanied by positive selection (or at least sequence divergence) at genes from the EMP pathway in specific yeast lineages (Fig 6A), consistent with several previous studies in *Dekkera* yeasts (Guo *et al*, 2016), *S. pombe* (Kamrad *et al*, 2020) and *S. cerevisiae* (Yu, Zhou, *et al*, 2018). However, from current evidences, it is not determined whether the specific gene selection contributes to the emergence of Crabtree effect or the evolution of Crabtree effect could accelerate the related gene selection, and, to some extent, these two events may possibly intertwine with each other during the long‐term evolution. Note that other evolutionary events are correlated to the Crabtree effect, such as rewiring of the transcriptional regulation network (Ata *et al*, 2018) and the loss of complex I (Dashko *et al*, 2014), indicating that multiple evolutionary events intertwined along the formation (or fitness) of complex traits in yeasts.

As reported, there exist multiple evolutionary trajectories for fungi to acquire the fitness of growth under high temperature (Mattoon *et al*, 2021). With integrative analysis of large‐scale genomics and trait data in yeast, we could infer amounts of potential gene features relevant for the formation of thermotolerance at a systems level. As a result, 141 positively selected genes were found to be important for the evolution of thermotolerance of yeast (Fig 6B), which is partially in agreement with experimental and *in silico* evidence of thermotolerance in *S. cerevisiae* (Li *et al*, 2021; Seike *et al*, 2021). Function enrichment analysis of these positively selected genes could help to find some interesting subpathways or GO terms correlated with thermotolerance. Combining experimental data by transferring genes from thermotolerant yeast to non‐thermotolerant yeast (Seike *et al*, 2021) initially showed that evolution of genes from subpathways underlying amino acid and protein synthesis may be a consistent clue underlying thermotolerance in yeast from comparative analysis of those multilayer datasets, whereas, to date, systematic experimental evidences are still lacking to verify the mechanistic correlation between the top positively selected genes (or unique mutations) and growth fitness under higher temperature. Thus, it is anticipated that a similar gene transfer between yeasts with distinct fitness can be designed for these top positively selected gene in future studies to further evaluate the polygenetic and/or pathway‐level evolution for the emergence of thermotolerance in yeast.

In summary, we have used comprehensive evolution analysis combined with ssGEM simulation to examine the evolution of diverse metabolic traits in yeast. We envision that this strategy can be widely applied in future studies to investigate the evolutionary mechanisms of additional fungal traits, such as pathogenicity (Román *et al*, 2007).

## Materials and Methods

### Reagents and Tools table

**Table:** 

Reagent/Resource	Reference or Source	Identifier or Catalog Number
**Experimental Models**		
*343 fungal species*	https://doi.org/10.1016/j.cell.2018.10.023 (Shen *et al*, 2018)	N/A
**Software**		
MAFFT v7.455	https://mafft.cbrc.jp/alignment/software/ (Katoh *et al*, 2005)	N/A
trimAl v1.2	http://trimal.cgenomics.org/ (Capella‐Gutiérrez *et al*, 2009)	N/A
BLAST+	ftp://ftp.ncbi.nlm.nih.gov/blast/executables/blast+/LATEST	N/A
Biopython	https://biopython.org/	N/A
Pfam v32.0	ftp://ftp.ebi.ac.uk/pub/databases/Pfam/releases/Pfam32.0	N/A
RAVEN v2	https://github.com/SysBioChalmers/RAVEN (Wang *et al*, 2018)	N/A
COBRA v3.0	https://github.com/opencobra/cobratoolbox/ (Heirendt *et al*, 2019)	N/A
TBLASTN	ftp://ftp.ncbi.nlm.nih.gov/blast/executables/blast+/LATEST	N/A
gapseq v1.1	https://github.com/jotech/gapseq (Zimmermann *et al*, 2021)	N/A
GECKO v2.0	https://github.com/SysBioChalmers/GECKO (Sanchez *et al*, 2017)	N/A
MACSE v2.03	https://bioweb.supagro.inra.fr/macse/ (Ranwez *et al*, 2011)	N/A
PhyloTreePruner v1.0	https://sourceforge.net/projects/phylotreepruner (Kocot *et al*, 2013)	N/A
FastTree v2.1	http://www.microbesonline.org/fasttree/ (Price *et al*, 2010)	N/A
GUIDANCE v2.0	http://guidance.tau.ac.il/ (Sela *et al*, 2015)	N/A
PAML v4.7	http://abacus.gene.ucl.ac.uk/software/paml.html (Yang, 2007)	N/A
HyPhy v2.5	https://github.com/veg/hyphy (Kosakovsky Pond *et al*, 2020)	N/A
ETE v3	http://etetoolkit.org/ (Huerta‐Cepas *et al*, 2016)	N/A
IQ‐TREE v1.6.12	http://www.iqtree.org/ (Nguyen *et al*, 2015)	N/A
CAFÉ v4.2.1	https://github.com/hahnlab/CAFE (Han *et al*, 2013)	N/A
scikit‐learn v0.22.1	https://scikit‐learn.org/stable/whats_new/v0.22.html	N/A

### Methods and Protocols

#### Module 1. GEM reconstruction and analysis

##### Genomic and phenotypic data collection for yeast species

Firstly, 332 sequenced genomes from the yeast subphylum and their related annotation were obtained from a previous study (Shen *et al*, 2018). Meanwhile, 11 fungal species (*Arthrobotrys oligospora, Aspergillus nidulans, Botrytis cinereal, Coccidioides immitis, Fusarium graminearum, Neurospora crassa, Saitoella complicate, Sclerotinia sclerotiorum, Stagonospora nodorum, Xylona heveae* and *Schizosaccharomyces pombe*) were selected as outgroup, and their genomes and related annotation were obtained from the JGI database according to the reported species ID from Shen *et al* (2018). All genes from these 343 fungal species were clustered into about 220,000 ortholog groups using OrthoMCL v2.0 (Li *et al*, 2003; Shen *et al*, 2018). The orthologs, coding sequences (CDS) and protein identifiers provided in the various datasets were mapped using custom Python scripts to gene identifiers and their respective protein sequences, while inconsistencies in identifier mapping were resolved by querying their corresponding sequences using reciprocal best BLAST hit (Moreno‐Hagelsieb & Latimer, 2008).

The phylogenetic information of all yeast species studied in this work is obtained from a previous study (Shen *et al*, 2018), where all species were divided into 12 main clades according to their phylogenetic distance. Their respective phenome data were systematically curated from literature in this work (Dataset EV1). Firstly, four main key phenotypes, i.e. “oleaginous”, “ethanol‐producing”, “pathogenic” and “thermotolerant”, were assigned for each species, by searching for literature containing the species name and phenotype‐related keywords, e.g. “lipid”, “oil”, “oleaginous” (Dataset EV2). Secondly, the substrate utilization profiles for 32 different substrates by 329 out of 332 yeast species were collected and evaluated from Kurtzman *et al* (2011) and Shen *et al* (2018).

##### Representative gene sequence for each ortholog group

To annotate the function of each of the 220,000 ortholog groups (OGs) defined from the 343 fungal genomes, representative genes were carefully selected from each OG according to two criteria: (i) if an OG contained *S. cerevisiae* sequences, then the *S. cerevisiae* sequence was selected as representative due to the *S. cerevisiae* genome annotation is the most mature and of high quality among all yeast species (if the OG contained multiple *S. cerevisiae* sequences, then only one *S. cerevisiae* sequence was selected as representative); and (ii) if an OG does not contain *S. cerevisiae* sequences, then we selected the longest sequence that has no unspecified (“X”) amino acid. Resultingly, 5,096 OGs have a member gene from *S. cerevisiae* S288c as representative sequence. The genes belonging to the same OG were assumed to have the shared function as the selected representative gene.

##### Reconstruction of pan‐GEM as the template model for all studied species

All modelling procedures were performed using COBRA toolbox v 3.0 (Heirendt *et al*, 2019) in MATLAB, unless noted otherwise. The KEGG web tool (Kanehisa & Goto, 2000), EggNOG web tool (Huerta‐Cepas *et al*, 2019) and RAVEN 2 toolbox (Wang *et al*, 2018) were used to annotate the collection of representative genes (from the pan‐genome) and identify reactions that did not already exist in the template model Yeast8 (https://github.com/SysBioChalmers/yeast‐GEM) (Lu *et al*, 2019). The protein FASTA file of representative genes was used to query the KEGG and EggNOG web services. In KEGG, the SBH (single‐directional best hit) method with default parameters was used, while for EggNOG, the HMMER method with the default parameters was used. In EggNOG, each protein was mapped onto KEGG Ontology (KO) identifiers and BiGG reaction identifiers, while in KEGG, each protein was given a unique KO identifier. Following this, new KO identifiers were mapped to KEGG reactions based on KO‐reaction identifier mapping. Lastly, the pan‐genome was annotated by reconstruction of a draft pan‐model from RAVEN using both the KEGG‐ and MetaCyc‐based functions (getKEGGModelForOrganism and getMetaCycModelForOrganism). The protein FASTA file of representative genes was used as input, while for KEGG, the pretrained HMM collection “euk90_kegg87” was used, and for MetaCyc, the pidentity was set at 55% and bitscore was set at 110.

All reactions annotated from the four approaches—KEGG web (Kanehisa & Goto, 2000), EggNOG (Huerta‐Cepas *et al*, 2019), RAVEN‐KEGG and RAVEN‐MetaCyc (Wang *et al*, 2018)—mentioned above were combined and compared with the reactions from template model Yeast8 (Appendix Fig S1I). Three criteria were applied for evaluating reactions to be included in the pan‐GEM: (i) unbalanced, reactions with generic reactants (e.g. “sugar”) and reactions containing “n” in the stoichiometry were discarded; (ii) new reactions that occurred in a previously reported pan‐fungal GEM (Correia & Mahadevan, 2020) were included; and (iii) reactions with more than 2 dead‐end metabolites were initially filtered out. For the new reactions, HMM‐based gene associations from KEGG, EggNOG and RAVEN‐KEGG were prioritized in comparison with the approach of homology search used by RAVEN‐MetaCyc. After collecting 562 new reactions together with their gene associations, they were added to Yeast8 to generate the pan‐GEM, now containing reactions from all studied species.

It is feasible that two different OGs are reflecting enzymes with the same catalytic activity, but due to sufficient sequence dissimilarity, they were divided into separate OGs. If one of the OGs has a *S. cerevisiae* member, then members of that OG will have already been added to the pan‐GEM. To also capture the other non‐*S*. *cerevisiae* groups of OGs, homologous pairs between representative OG genes and all genes from Yeast8 were searched using reciprocal best BLAST with pidentity as 70% (Appendix Fig S1G). 1,201 homologous pairs were determined and were updated in the gene associations of the pan‐GEM according to the following rule: homolog gene C would be updated to all gene associations of its reference gene A: (i) the original gene association “A or B” would be changed to “A or B or C”, and (ii) the gene association “A and B” would be changed to “(A and B) or (C and B)”.

##### Generation of yeast species‐specific GEMs (ssGEMs)

For each yeast species, a copy of pan‐GEM was created and then manipulated by removing non‐existing genes and reactions using the pan‐gene existence matrix that was generated from the ortholog group annotation by OrthoMCL. Based on gene existence information, if more than 50% subunits of an enzyme complex were present, then the complex was considered to be active in that species, and the corresponding reaction was therefore kept in the ssGEM, while its gene association was updated to remove the missing subunits. For reactions associated with isozymes, if one enzyme among them is missing, then the reaction was also kept in the ssGEM, while its gene association was again updated to remove the missing gene. Generally, reactions without gene association rules in the pan‐GEM, such as spontaneous reactions and exchange reactions derived from Yeast8, were kept in each ssGEM. In these initial pan‐GEM derived ssGEM, the representative gene identifiers were updated to the species‐specific gene identifiers, while the representative identifiers were saved in the SBML file of each model to facilitate further analysis. In a species, multiple homolog genes may exist for the same representative gene. The rule for updating homologs in the ssGEMs is the same as for the homolog update for pan‐GEM mentioned above. Homologs would be updated as “or” relation in the GPR rule expression. As for complexes with multiple subunits, the complex would be copied and updated.

##### Biomass definition for ssGEMs

The various individual components (e.g. all amino acids, nucleotides, ribonucleotides) that make up biomass in pan‐GEM were assumed to be comparable to *S. cerevisiae*, and therefore, the biomass definition was borrowed from Yeast8, while cofactors were removed as the detailed gene annotation for cofactor synthesis pathways in most non‐model yeast species is not complete. In the pipeline of ssGEM generation, the biomass pseudoreactions from pan‐GEM were transferred with adjustments. Since all yeast and fungal species in this work were classified into four main phenotype groups (i.e. normal, heat‐tolerant, oleaginous and pathogenic), macrobiomass compositions in aspects of lipid, carbon, protein, DNA and RNA for those four groups were referred to the biomass compositions in representative species (with published GEMs, Dataset EV2) from the above four main phenotype groups. Also, proportions of macrobiomass components such as protein, lipid, DNA and RNA were scaled accordingly.

##### Gap‐filling

RAVEN was used to construct draft models for each individual species. With the species protein FASTA file as input, two draft models were generated for each species, based on KEGG and MetaCyc, respectively, and served as reference for later gap‐filling of the ssGEMs if needed. These models are referred to as RAVEN draft GEM (KEGG or MetaCyc) to distinguish from the ssGEMs. Next, gap‐filling was adopted to guarantee that each ssGEM could support cellular growth in minimal growth media (free uptake of ammonia, oxygen, phosphate, sulphate and a constrained uptake of glucose). For auxotrophic species, including *Eremothecium coryli* and *Tetrapisispora blattae* (Hagman *et al*, 2013), additional auxotrophic compounds were added to the minimal media during simulation (L‐lysine for *E. coryli* and complex media for *T. blattae*). For ssGEMs that could not achieve growth under above conditions, gap‐filling was utilized to identify and include missing reactions.

In detail, to include reactions that are essential for biomass synthesis, it was first checked which of the biomass components could not be synthesized, and canonical pathways from MetaCyc were used to identify the missing reactions. The single‐species RAVEN draft GEMs (KEGG and MetaCyc) were subsequently queried, and if both RAVEN draft GEMs contained the missing reactions, then these reactions and their related gene associations would be added into the ssGEM. This approach ensured that those enzymes with gene sequences too deviant from the representative genes in the OGs could still be captured and included in the model. The pan‐GEM was furthermore updated accordingly to encompass the new changes. Only gaps that could be filled with gene‐associated reactions from the RAVEN draft GEMs were resolved with this approach. Complementarily, automatic gap‐filling was performed for the remaining gaps to enable growth. The “fillGaps” function from RAVEN toolbox (Wang *et al*, 2018) was used to determine the missing reactions where the pan‐GEM acted as a universal database of possible reactions. For this, “useModelConstraints” was set as true and the lower bound of the biomass synthesis reaction was set at > 0. This method detects the minimal number of reactions that should be added from the pan‐GEM to satisfy the *in silico* growth in each ssGEM. After these two gap‐filling steps (Appendix Fig S1K), all ssGEMs were able to predict growth.

##### Model quality improvement based on substrate utilization evidence

Experimental evidence on substrate utilization by yeast species was used to evaluate model predictions and iteratively improve model quality. When testing whether a yeast species could utilize specific substrate *in silico*, the ssGEMs were constrained under minimal media and replacing with the corresponding carbon or nitrogen source. Growth on different carbon and nitrogen substrates was simulated by allowing exchange of the corresponding substrate with a rate of −10 mmol g_DW_^−1^ h^−1^, and threshold of growth rate as 10^−6^ h^−1^. Literature‐based candidate reactions to enable substrate utilization were manually collected for gap‐filling (Dataset EV5). TBLASTN was used to predict the existence of the corresponding enzymes (and the associated reactions) in 332 yeast genomes based on the existing protein sequences in KEGG, which are connected with those candidate reactions. In TBLASTN analysis, the strict cut‐off was set as: bitscore > 50, *E*‐values < 1e‐10, coverage > 70% and identity > 30%. In order to increase accuracy, the protein sequences from fungal species were prioritized; otherwise, 30 randomly selected sequences were used as reference for each enzyme. TBLASTN was further used to conduct gene mining to determine which pathways exist in related fungi. For example, erythritol degradation has two alternative pathways. Most sequences for enzymes in erythritol degradation I were retrieved from KEGG, except for sequence “Q2YIQ3” (*Brucella abortus* strain 2308) for eryC (EC 5.1.3.38), which was extracted from UniProt based on annotation in MetaCyc due to the missing KO annotation for eryC in KEGG. Due to the missing reactions for erythritol degradation II in KEGG and MetaCyc, the reported proteins “YALI0F01606g” and “YALI0F01650p” from *Y. lipolytica* (Carly & Fickers, 2018) were used as query sequences, which were obtained from UniProt. As for lysine degradation pathway, gap‐filling reactions were directly added to the corresponding ssGEMs and pan‐GEM based on literature (Zabriskie & Jackson, 2000), due to missing KO and gene annotation in KEGG and MetaCyc. After the above curation, substrate utilization prediction accuracy (equation 1) was calculated for each species according to:

(1) $$Accuracy=\;\frac{TP\;+\;TN}{TP+TN+FP+FN}$$

where *TP*, *TN*, *FP* and *FN* denote true positive, true negative, false positive and false negative, respectively.

##### Model validation with reported essential gene and key evolution traits

To further evaluate model quality, the functional characteristics of ssGEMs were investigated by gene essentiality analysis. Experimental evidence on essential genes for five yeast species, *P. pastoris*, *S. cerevisiae*, *S. pombe*, *Y. lipolytica* and *C. albicans*, was collected from literature and the Database of Essential Genes (DEG) (Luo *et al*, 2014) (Dataset EV2). In silico gene essentiality was predicted by enumerating gene knockout for each ssGEM and simulating growth using flux balance analysis (FBA) (Orth *et al*, 2010), where a simulated maximal growth rate < 10^−6^ h^−1^ was classified as no growth and the corresponding gene can be regarded as an essential gene (Heavner & Price, 2015). The ssGEMs could further be used to characterize the loss of respiratory chain complex I (Hagman *et al*, 2013) (Appendix Fig S2B) in corresponding species by examining the existence of corresponding reactions in ssGEMs.

##### Definition of high‐correlated reactions contributed to substrate utilization

Correlation between enzyme/reaction existence and substrate uptake was quantified as accuracy and sensitivity (equations 2 and 3). As it is widely known that the ability of yeast species to utilize galactose is highly correlated with the existence of GAL1, GAL7 and GAL10 (Opulente *et al*, 2018) in their genomes, we repurposed the corresponding accuracy (0.83) and sensitivity (0.92) for Gal1 (or Gal7/Gal10) and used the similar approaches to define high‐correlated reactions contributing to the normal utilization of the remaining substrates used in this work.

(2) $$Accuracy=\;\frac{TP\;+\;TN}{TP+TN+FP+FN}$$

(3) $$Sensitivity=\;\frac{TP\;}{TP+FN}$$

##### Definition of gain of new traits in substrate utilization occurred in each yeast species

Phenotypes (e.g. substrates utilization) for each species were compared with their ancestral budding yeast common ancestor (BYCA) phenotype to calculate the number of gain/loss events in the substrate utilization. The posterior probability of ancestral state in BYCA for each metabolic trait was collected from a previous study (Shen *et al*, 2018). Here, a posterior probability of 0.85 was used as a cut‐off for the existence of a phenotype in BYCA, while a probability lower than 0.15 was interpreted as non‐existence. Through this, among 32 traits in substrate utilization which could be connected with the metabolites in ssGEMs, 5 traits were classified as gain of new function in utilizing carbon (2‐Keto‐D‐gluconate, D‐arabinose, D‐ribose, methanol) and nitrogen (nitrite) sources (Fig 5A).

##### Transporter annotation for substrate utilization

Annotation of transporter reactions were conducted for each species by combining the pan‐GEM reconstruction‐derived annotations and additional direct BLAST search for each species. For the latter, BLAST was performed using the function—“gapseq find‐transport” in gapseq (Zimmermann *et al*, 2021) for protein sequences of each species against the Transporter Classification Database (Saier *et al*, 2016) with default parameters (bitscore ≥ 200 and coverage ≥ 75%). Among the 32 substrates identified in the previous section, 18 were annotated to the potential transporters in this pipeline. Those annotations were later used to identify genes acquired via horizontal gene transfer (HGT).

##### Model similarity analysis for ssGEMs

To compare similarity between all ssGEMs, the “compareMultipleModel” function from RAVEN toolbox was used. Binary reaction existence matrix was collected to calculate Hamming distance. Three‐dimensional t‐Distributed Stochastic Neighbour Embedding (t‐SNE) was used to visualize the classification of ssGEMs (Fig 2D).

##### ecModels reconstruction and flux control coefficient analysis

In order to incorporate enzyme constraints into ssGEMs, the GECKO toolbox v1.3.5 was used to generate ecModels in accord with the procedure in our previous work (Sanchez *et al*, 2017). Maximum growth rates for each species were collected from literature (Hagman *et al*, 2013) (Dataset EV2) and were used to curate growth‐limiting *k*_cat_ parameters in an iterative procedure until the ecModels reached the provided experimental values. Additionally, experimental ethanol production rates from the literature were incorporated as constraints prior to the iterative automated curation of *k*_cat_ values, in order to obtain kinetic profiles that reflect the observed phenotypes for each of these species.

In order to investigate the limitations imposed by individual enzyme activities on a given reaction flux, flux control coefficients can be calculated by inducing small perturbations for each enzyme in the model according to the following definition (Nilsson & Nielsen, 2016):

(4) $${\mathrm{FCC}}_{i}=\left(\frac{k_{\mathrm{cat}}^{\mathrm{ij}}}{v_{j}^{*}}\right)*\left(\frac{v_{j}^{*}‐v_{j}}{1.001k_{\mathrm{cat}}^{\mathrm{ij}}‐k_{\mathrm{cat}}^{\mathrm{ij}}}\right)$$

where *v*_j_ represents the original flux for reaction *j* in a reference flux distribution; $k_{\mathrm{cat}}^{\mathrm{ij}}$ is the turnover number for the enzyme *i* in reaction *j*; and $v_{j}^{*}$ is the resulting flux for reaction *j* after inducing a perturbation of 0.1% in the activity of enzyme *i*. For the calculation of flux control coefficients over the growth rate of ecModels, the biomass exchange pseudoreaction (cellular growth) was taken as *v*_j_, assuming YPD medium with D‐glucose as a carbon source for all cases.

##### *In silico* evaluating the influences of enzyme parameters on the Crabtree effect

The effects of selected enzyme activities on phenotypic traits related to the Crabtree effect in *S. cerevisiae* (biomass yield, ethanol production yield and critical dilution rate) were quantitatively assessed using ecYeast8 (Lu *et al*, 2019). The biomass yield on fermentative conditions and ethanol production yield were both estimated from enzyme‐constrained parsimonious flux balance analysis (ec‐pFBA) (Österberg *et al*, 2021), using maximization of biomass production as an initial objective function, following by minimization of the total protein pool utilization, subject to maximum biomass production, in order to obtain biologically meaningful fluxes. Both yields were normalized by gram of consumed glucose. This procedure was repeated for several values of perturbed *k*_cat_ values for the selected enzymes, ranging from onefold to 10‐fold of the original value (Appendix Fig S8F). Additionally, a reduced proteome‐constrained model that contains reactions of energy metabolism of *S. cerevisiae* (Chen & Nielsen, 2019) was also used to explore how the perturbed *k*_cat_ values of the selected enzymes influence exchange fluxes. To do so, the default model was employed to predict exchange fluxes at maximal growth as a control, and then, the protein cost of the related reaction was decreased at a time from the default value (100%) to 10% (it means the corresponding *k*_cat_ increased from onefold to 10‐fold). The resulting exchange fluxes were compared with those of the control.

#### Module 2. Evolution analysis at gene level

##### Ortholog quality analysis

For the pan‐GEM and ssGEM reconstructions, the original ortholog groups (OG) were used directly. Contrastingly, for the following evolution analysis, the OGs firstly underwent systematic quality analysis to remove those OGs with too many paralog genes or containing too few yeast species, as such cases would impede further analysis. Quality analysis was performed through the following steps.

1. For OGs with at least seven species, the relevant CDS sequences were collected in a new FASTA file, thereby keeping 15,461 OGs as most groups contained less than seven species.
2. All CDS within an OG were translated into protein sequences using translateNT2AA from MACSE v2.03 (Ranwez *et al*, 2011) and subsequently aligned to the other sequences within the OG using MAFFT v7.455 (Katoh *et al*, 2005) in auto mode.
3. Gene trees were built by firstly trimming the aligned protein sequences using trimAl v1.2 (Capella‐Gutiérrez *et al*, 2009) with the gap cut‐off set to 0.7. Proteins that were trimmed to less than 50% of the aligned length were filtered out. Spurious sequences were removed by again using trimAl, now with resoverlap at 0.75 and seqoverlap at 0.75. After the above initial filtration, 13,652 OGs with species number ≥ 7, paralogs per single species ≤ 5, ratio of sequences over species ≤ 1.5 and remaining residues for each protein ≥ 30 were retained. This collection of 13,652 OGs was either used directly in the following evolution analysis, while for particular analysis (e.g. gene‐level dN/dS calculation), the OGs were further reduced to remove paralog genes, yielding single‐copy OGs. For this, a tree pruner strategy was followed where gene trees for the 13,652 OGs were built using FastTree v2.1 (Price *et al*, 2010) with the Le‐Gascuel 2008 model, and the gene trees were subsequently pruned using PhyloTreePruner v1.0 (Kocot *et al*, 2013) with bootstrap cut‐off at 0.95 and picking the longest sequence for a given out, retaining 13,220 single‐copy OGs.
4. Protein alignments within each OG were used to align the corresponding CDS sequences using reportGapsAA2NT from MACSE and GUIDANCE v2.0 (Sela *et al*, 2015). The CDS alignment and quality analyses from GUIDANCE were used in the following site model and “branch‐site” model analyses, and the CDS alignments from MACSE were used to connect the site‐wise dN/dS with the coordinates of residues from protein 3D structures. Occasional stop codons found in the aligned CDS sequences obtained from MACSE were replaced with gaps.

##### dN/dS calculation

The gene‐level dN/dS of paired gene sequences from the same OGs were calculated with yn00 from PAML v4.7 (Yang, 2007), using the 13,220 single‐copy OGs as input. Median values were used for comparison between different OGs (Fig 3A), while any codon with gaps across species was removed automatically by yn00. The gene‐level dN/dS values were extracted from the PAML output file. To reduce bias in the statistical analysis, the dN/dS for gene pairs with dS ≥ 3 or dS ≤ 0.005 were removed. Note that though with higher efficiency, the yn00 is of lower quality and accuracy than the M models.

Positively selected sites were identified in the aligned CDS sequences across yeast species using HyPhy v2.5 (Kosakovsky Pond *et al*, 2020) and PAML v4.7 (Yang, 2007) (Appendix Fig S5A), where for the former, both the FEL (Kosakovsky Pond & Frost, 2005) and FUBAR (Murrell *et al*, 2013) (Materials and Methods) were used with their default parameters. As calculations using the site model of PAML take much longer, especially for large OGs, primarily results from HyPhy were considered in this work. HyPhy output was processed with phyphy v0.4.3 (https://github.com/sjspielman/phyphy) to extract the site information. Sites with dN/dS > 1 and posterior probabilities either larger than 0.9 in FUBAR or with corrected *P* value smaller than 0.1 in FEL were regarded as under positive selection.

##### Branch‐site model for selected gene analysis

To connect the positively selected genes with traits in specific lineages of yeast species, aBSREL (Smith *et al*, 2015) (adaptive Branch‐Site Random Effects Likelihood) from HyPhy was adopted to conduct positive selection analysis using the “branch‐site” model (Appendix Fig S5A). To detect positively selected genes, species with specific traits, i.e. Crabtree effect and heat tolerance, and monophyletic groups of the related species (nodes) (Weber *et al*, 2020) were labelled as the “Foreground” in each gene tree, while the remaining were labelled as the “Background”. The gene tree approach is used in this work as only a subset of OGs have at least one corresponding sequence for each of the species from the species tree. To improve calculation efficiency, six species were randomly selected from each main clade that do not have the specific trait. If a main clade had over six species with the specific trait, then six species were randomly selected from that clade. Also, if a main clade contained species with and without the specific trait at the same time, then the species without the specific trait was removed from the “branch‐site” model analysis. If multiple paralog genes were found for the same species, then the gene with longest protein after quality analysis was selected as representative in the analysis. Using such a unified procedure, nearly all species with the “heat tolerance” trait could be labelled as “Foreground” (test) branch. Through computation and comparison, it was found that the taxonomy sampling strategy used here, to some extent, could balance the accuracy and efficiency in large‐scale evolution analysis as this is very computation‐intensive. For the “Crabtree effect” trait, only OGs from the core metabolic pathways and transcript factors were used in the calculation as they are expected to be highly related to the emergence of the Crabtree effect. In all “branch‐site” model analysis, OGs with at least three species with the trait and at least three species without the trait were used in the calculation. Meanwhile, to reduce the bias from random sampling, for each trait studied in this work, two independent calculations were carried out, while the intersection of the positively selected genes from the two calculations was used in the final analysis. Additional calculation was conducted as the null hypothesis analysis to test the above procedure. In this null hypothesis analysis, 25 species were randomly selected from the 76 Crabtree‐negative species as the foreground branches in the “branch‐site” model analysis. It initially shows that the positive selection based on the normal “branch‐site” model analysis is obviously different from the result with the null hypothesis analysis (Dataset EV7).

##### Gene tree reconstruction in positive gene (site) selection analysis

For each OG after quality analysis (see Ortholog quality analysis), gene trees were reconstructed using FastTree v2.1 (Price *et al*, 2010) with the Le‐Gascuel 2008 model for the above site model and “branch‐site” model analysis. As a whole, over 13,000 gene trees were reconstructed, which can be further visualized using iTOL v5 (Letunic & Bork, 2019) and ggtree v1.14.6 (Yu, Lam, *et al*, 2018). Note that though with higher efficiency, FastTree v2.1 (Price *et al*, 2010) is of lower accuracy in gene tree inference.

##### Horizontal gene transfer analysis

To determine horizontal gene transfer (HGT) events from non‐fungal organisms to yeast, BLASTP was run against the NCBI non‐redundant (nr) protein sequence database by taking a collection of proteins related to substrate utilization and transporters as input (Camacho *et al*, 2009). BLAST hits were parsed to retrieve associated taxonomic information using ETE v3 (Huerta‐Cepas *et al*, 2016). Alien Index (AI) scores (Gladyshev *et al*, 2008) were calculated to predict horizontal gene transfer events with the formula

(5) $$AI=\text{ln}\left(bbhG+1*10^{‐200}\;\right)‐\text{ln}\left(bbhO+1*10^{‐200}\right)$$

where bbhG is the *E*‐value of the best BLAST hit to a species within the group lineage (fungi) but outside of the subphylum (*Saccharomycotina*), and bbhO is the *E*‐value of the best BLAST hit to a species outside of the group lineage (fungi).

To further remove contamination results of HGT, the percentage of species from outside of the fungi lineage (out_pct) was determined according to the BLAST hits. As a result, those 32 and 33 genes with AI ≥ 45 and out_pct ≥ 90% were grouped as potential HGT candidates from outside of fungi in terms of substrates utilization and transporters, respectively (Gladyshev *et al*, 2008; Marcet‐Houben & Gabaldón, 2010) (Dataset EV5).

To identify HGT events from closely related organisms (fungi) to yeast, a combination of BLAST similarity searches and comparative similarity index (HGT index) was investigated to predict HGT events (Crisp *et al*, 2015). Firstly, for each gene associated with substrate utilization, BLASTP against NCBI nr protein database and taxonomy determination were conducted as described above. Then, several steps were carried out to screen potential genes acquired via HGT from fungi as follows:

1. Genes with a best hit in another fungi lineage (excluding the recipient *Saccharomycotina*) and a bitscore ≥ 100 were defined by the first round of preliminary screening;
2. The percentage of species from other fungi (inside of fungi, outside of Ascomycota) was determined, if this was ≥ 90%, then the gene was retained;
3. HGT index was calculated as the bitscore of the best hit in other fungi divided by the bitscore of the best hit in recipient (*Saccharomycotina*), where all genes with HGT index ≥ 50% were retained, as this indicated that these genes match well to other fungi genes. These parameter thresholds were based on previous reports (Marcet‐Houben & Gabaldón, 2010; Crisp *et al*, 2015; Shen *et al*, 2018). Finally, 153 and 78 genes were found as putative genes obtained by HGT from fungi in association with substrate utilization and transporters, respectively.

To ensure that the high‐throughput HGT identification approach yielded accurate predictions, a phylogenetic analysis strategy was adopted for further manual inspection. For each candidate gene, homologs were selected according to the top 300 BLAST hits to each query sequence. These homologs were then aligned with MAFFT v7.310 (Katoh *et al*, 2005) using default settings for multiple sequence alignment. Poorly aligned regions were removed with trimAl (Capella‐Gutiérrez *et al*, 2009) using the “‐automated1” option. Subsequently, phylogenetic trees were built using IQ‐TREE v1.6.12 (Nguyen *et al*, 2015) with 1,000 ultrafast bootstrapping replicates (Minh *et al*, 2013). Each tree was rooted at the midpoint using a customized script by combining R packages ape v5.4.1 and phangorn v2.5.5. Finally, the resulting phylogenies were visualized using iTOL v5 (Letunic & Bork, 2019), through manually checking them one by one to assess the mode of transmission of each gene, 102 and 61 potential gene hits were identified in relation to substrate utilization and transporters, respectively (Dataset EV5).

##### Gene family expansion and contraction analysis

Gene family expansion and contraction across yeast species were investigated using CAFÉ v4.2.1 (Han *et al*, 2013) with default parameters. The software CAFÉ uses a birth and death process to model the evolution of gene family sizes by a phylogenetic tree, in which gene family sizes were obtained by a customized script based on OG. For each gene family, CAFÉ generated a family‐wide *P* value along specific species or branches, with a significant *P* value (< 0.05) indicating a possible gene family expansion and contraction event. More gene families are prone to contraction compared with expansion across species (Appendix Fig S7A and B), consistent with an earlier report that reductive evolution is the major mode causing evolutionary diversification (Shen *et al*, 2018).

##### Conservation score calculation for each residue site of proteins

Residue‐site conservation scores were calculated using a reported method based on the Jensen–Shannon divergence (JSD) (Capra & Singh, 2007), by which the gap cut‐off is set at 0.3 and js_divergence is used as a conservation estimation method. To compare the residue conservation score from different algorithms, the ConSurf Server (Ashkenazy *et al*, 2016) was adopted for several examples. A linear correlation was found between the JSD and ConSurf results (*R* ^2^ = 0.83), and the JSD method was selected for conservation score calculation for all ortholog proteins.

##### Functional enrichment analysis of positively selected genes

Once genes with at least one positively selected residue site were obtained, KO annotations of the representative genes were used to connect all positively selected genes with KEGG subpathways (Kanehisa & Goto, 2000). Subsequently, frequencies of subpathways of all positively selected genes were calculated to obtain the top subpathways with relatively more positively selected genes (Appendix Fig S5C). For positively selected genes from “branch‐site” model analysis relevant for the trait of heat tolerance, as 140 of 141 OGs have a member gene from *S. cerevisiae* S288c, the gene IDs from *S. cerevisiae* S288c were directly used in the function enrichment analysis with aid of DAVID (Huang *et al*, 2007).

##### Unique mutation analysis related to heat tolerance

Potential unique mutations related to heat tolerance were identified by selecting and aligning protein sequences from yeast species with and without heat tolerance. From this, the distribution of residues at each coordinate from the two groups of yeast species was calculated. At the first glance, taking the strict definition that unique mutations are defined as a residue that only occurred in yeast species with heat tolerance, no related residues could be identified. To widen the search, specific residues that occurred in over 80% of yeast species with heat tolerance were defined as conserved and highly correlated with heat tolerance. Subsequently, all residues that occurred in over 20% of the yeast species without heat tolerance were defined as the reference residue set. In the last step, if a conserved residue occurred in yeast species with heat tolerance but not within the reference residues set, then this residue was regarded as a potential unique mutation, which could possibly contribute to the formation of heat tolerance. In order to map the unique mutation onto protein 3D structure, only the mutation having the corresponding reference from *S. cerevisiae* S288c was selected in all the relevant analysis.

#### Module 3. Evolution analysis at residue‐site level with aid of protein 3D structure

##### Protein 3D structure collection and quality analysis

For all proteins from *S. cerevisiae* S288c, the homology‐modelled protein structure files (PDB) built by the SWISS‐model database (Waterhouse *et al*, 2018) and the experimentally determined protein structures stored in PDB files at RCSB protein data bank (Rose *et al*, 2017) were collected. In quality analysis, the homology PDBs with QMEAN < −4 for proteins without experimental PDB files were filtered out according to the description from SWISS‐model database (Waterhouse *et al*, 2018). In total, 3,567 *S. cerevisiae* proteins have the related homology PDB files after the quality analysis during this step (Appendix Fig S6A–C). For the experimental (instead of modelled) PDBs, the correct chain in the 3D structure was extracted using SIFTS (Velankar *et al*, 2013). Next, BLAST was used to align the protein sequences in the PDB files with the reference protein sequences from *S. cerevisiae* S288c, and PDB files with pidentity at 100% and no gaps were kept. As a result, experimental PDB files for 918 proteins can be used in the following analysis, while, if all experimental PDBs for one protein were filtered out, the homology PDB files will be used. After all the quality analysis, the custom python scripts were applied to calculate the distance matrix of paired C‐atoms for all the PDB files (Meyer *et al*, 2016), which will be used as the following cluster analysis based on the protein structure information.

##### Functional annotation of residues sites with UniProt

Annotations of functional site for *S. cerevisiae* S288c were collected from UniProt (The UniProt Consortium, 2017) (https://www.uniprot.org/help/sequence_annotation) and used as reference to study how functional sites evolve. The protein secondary structures were predicted using SCRATCH (Cheng *et al*, 2005). The detailed phosphorylation sites and interface prediction of protein 3D structures were acquired from Lanz *et al* (2021). This involved annotating amino acid residues with sites of various types including active sites, metal binding sites, other binding sites, interfaces of protein complex, secondary structure and phosphorylation site to be used in further analysis (Fig 4D).

##### CLUMPS analysis of fast‐evolved sites and positively selected sites for a protein

Positively selected sites were clustered based on protein 3D structures. Firstly, the positively selected site for one (unpruned) OG from FUBAR (posterior probability > 0.9) was used, and the relative coordinates of these positively selected sites on the representative proteins were obtained through coordinate mapping. With the relative coordinates of the positively selected site and the structure distance matrix, it was calculated whether these positively selected sites were significantly enriched in specific zones within the protein 3D structure using CLUMPS analysis (Kamburov *et al*, 2015). Briefly, in original CLUMPS analysis, a *P* value is calculated based on a null distribution by randomly distributing a mutated residue within protein 3D structures. In this work, the mutated residue is replaced by the positively selected site.

Some of the OGs have less than 2 strong positively selected sites, which is not enough for the above enrichment analysis based on protein 3D structures, and would thereby omit many fast‐evolved sites (dN/dS > 1). To prevent this, the fast‐evolved sites were used as input for the CLUMPS analysis, to further explore how fast‐evolved sites distributed spatially within the protein 3D structures (Fig 4A).

#### Module 4. Machine learning for the improved essential gene prediction in genome scale

##### Sequence data collection used for machine learning

Reported essential gene datasets from five yeast species (*P. pastoris*, *S. cerevisiae*, *S. pombe*, *Y. lipolytica* and *C. albicans*) were collected and used to build machine learning models for the prediction of essential genes (Dataset EV2). The gene and protein sequence FASTA files used for *S. cerevisiae*, *C. albicans* and *S. pombe* were acquired from SGD (Cherry *et al*, 2012), CGD (Skrzypek *et al*, 2018) and PomBase (Lock *et al*, 2019) database, respectively, while the gene and protein data for *P. pastoris* and *Y. lipolytica* were all obtained from the NCBI RefSeq database (Pruitt *et al*, 2007).

##### Feature calculation for prediction of essential gene using machine learning model

Gene essentiality can be predicted by machine learning based on sequence‐derived properties (Ning *et al*, 2014). For sequence features, Dinucleotide composition (DNC) and codon frequency have been recognized as important sequence features for essential gene prediction (Ning *et al*, 2014; Lin *et al*, 2019), where Kmer is characterized as the codon frequencies can be represented as Kmer of k neighbouring nucleic acids for a specific DNA sequence (Chen *et al*, 2020). Therefore, for the collected essential gene datasets from five yeast species, the sequence features here were characterized by DNC and Kmer (*k* = 3), which can be calculated by:

(6) $$DNC\left(r,\;s\right)=\frac{N_{\mathrm{rs}}}{N‐1}\;r,\;s\;\in \left\{A,\;C,\;G,\;T\right\}$$

(7) $$Kmer\left(t\right)=\frac{N\;(t)}{N}\;t\;\in \left\{AAA,\;AAC,\;AAG,\;\cdots ,\;TTT\right\}$$

where *N_rs_* is the number of combinations of any two nucleic acid *r* and *s*, *N* (*t*) is the number of type *t*, and *N* is the length of a nucleotide sequence.

In addition to the above features that were directly extracted from gene sequences, evolution‐based features, including protein conservation score, dN/dS, number of gene occurrence across species and average paralog number, were calculated for each gene based on its ortholog information (Appendix Fig S4A). The protein conservation score is defined as the average conservation scores of all residues for one OG. The average paralog number is defined as the number of sequences contained in one OG divided by the total number of unique species in that OG.

##### Machine learning (ML) workflow for the prediction of essential gene

To establish the ML models, different approaches were adopted to divide all curated datasets into training datasets and testing datasets. In the first approach, 80 and 20% of all genes were randomly selected as training and testing dataset. In the second approach, four yeast species were chosen as training dataset, with the remaining species as testing dataset. In order to improve the performance of our machine learning predictor, the datasets were balanced by oversampling the minority class instances (Lanera *et al*, 2019). To investigate whether the evolution‐based features would help to enhance the prediction of essential genes using ML, both the support vector machine (SVM) and random forest (RF) algorithms were applied. All ML models were implemented in scikit‐learn v0.22.1.

##### ML prediction performance evaluation

Several standard evaluation metrics comprising recall (or sensitivity), specificity, accuracy, precision and F1 score were adopted to assess the prediction performance of the ML models. The evaluation metrics were calculated as follows:

(8) $$True\;Positive\;Rate\;\left(or\;Recall\;or\;Sensitivity\right)=\frac{\mathrm{TP}}{TP+FN}$$

(9) $$Specificity\;=\frac{\mathrm{TN}}{TN+FP}$$

(10) $$Accuracy\;=\frac{TP+TN}{TP+TN+FP+FN}$$

(11) $$Precision\;=\frac{\mathrm{TP}}{TP+FP}$$

(12) FalsePositiveRate=1‐Specificity

(13) $$F1\;score=\frac{2*Precision*Recall}{Precision+Recall}$$

where *TP*, *TN*, *FP* and *FN* denote true positive, true negative, false positive and false negative, respectively.

To evaluate the performance of different ML models, the receiver operating characteristic (ROC) curve was utilized through fivefold cross‐validation. The ROC curve was plotted with false positive rate (FPR) on the *x*‐axis and true positive rate (TPR) on the *y*‐axis. The higher the area under the ROC curve (AUC) value, the better performance the machine learning model has in prediction. When comparing gene essentiality prediction by using sequence features alone and by combining sequence features with evolution‐based features, it could be found that the AUC values for essential gene prediction on testing dataset with SVM and RF algorithm were improved from 0.65 to 0.81 and 0.65 to 0.80, respectively (Appendix Fig S4C and D). For features' contribution analysis, all of the features were set as the input in the ML, and chi‐square test (Chen *et al*, 2020) was used to rank features according to their contribution to the ML prediction.

#### Quantification and statistical analysis

For two group comparisons in this work, a two‐tailed Wilcoxon rank sum test was calculated.

## Author contributions

HL and JN designed the project. HL contributed to model reconstruction and the evolution analysis in the paper. FL contributed to model reconstruction and analysis in the paper. LY contributed to model reconstruction, the horizontal gene transfer analysis and the machine learning of essential genes in the paper. ID contributed to ecGEM reconstruction and prediction. GL contributed to the gene family analysis. YC contributed to the simulation of Crabtree effect. BJ contributed to the evolution analysis. EJK contributed to the design and guidance of the project. RY and HW gave comments about the content in paper. HL, FL, LY, EJK and JN prepared the manuscript and figures.

## Conflict of interest

The authors declare that they have no conflict of interest.

## Supporting information



Appendix

Dataset EV1

Dataset EV2

Dataset EV3

Dataset EV4

Dataset EV5

Dataset EV6

Dataset EV7

Source Data for Figure 2

Source Data for Figure 3

Source Data for Figure 4

Source Data for Figure 5

Source Data for Figure 6

## Data Availability

More detailed results in this study are available on https://figshare.com/articles/dataset/Comprehensive_evolution_analysis_with_genome_scale_metabolic_models_reveals_diverse_mechanisms_in_metabolic_innovations_across_332_yeast_species/14473776. GEMs for 343 yeast/fungi species are available in the BioModels database (www.ebi.ac.uk/biomodels) with accession numbers MODEL2109130002, MODEL2109130004‐MODEL2109130011, MODEL2109130013, MODEL2109130014, MODEL2109240001 and MODEL2109240002. All scripts are recorded using a version control system, Git, and hosted in three GitHub repositories: metabolic model reconstruction in https://github.com/SysBioChalmers/Yeast‐Species‐GEMs; evolution analysis in https://github.com/SysBioChalmers/Multi_scale_evolution; and essential gene prediction in https://github.com/SysBioChalmers/MLEssential.
