# Supplementary material for: Yeast metabolic innovations emerged via expanded metabolic network and gene positive selection
Source: Mol Syst Biol. 2021 Oct 22;17(10):e10427. doi: 10.15252/msb.202110427 (PMC8532513; doi:10.15252/msb.202110427)
Supplement: Supplementary file 1 — Appendix [file MSB-17-e10427-s014.pdf]

Appendix Figures

Appendix Figure S1 Genome evolution and annotation for 343 fungal species. 2

Appendix Figure S2 Evaluation of reconstructed ssGEMs in pathway mining and metabolic coverage. 4

Appendix Figure S3 Prediction of essential genes and physiological data using ssGEMs and ecGEMs. 6

Appendix Figure S4 Improved essential gene prediction guided by multi-dimensional evolutionary parameters. 8

Appendix Figure S5 Evolution analysis in codon (amino acid residue) level. 10

Appendix Figure S6 Statistical analysis of protein 3D structure for S. cerevisiae S288c used in this work. 11

Appendix Figure S7 Gene family expansion and contraction analysis together with horizontal gene transfer analysis to illustrate the mechanism in the metabolic innovation occurred in yeasts. 12

Appendix Figure S8 The mechanism for the generation of Crabtree effect in yeasts is further illustrated by the genetic data, physiological data and ecGEM simulation. 14

Appendix Figure S9 The mechanism for the generation of heat-tolerance in yeasts is further illustrated by the genetic data and proteomic data. 16

Reference 18

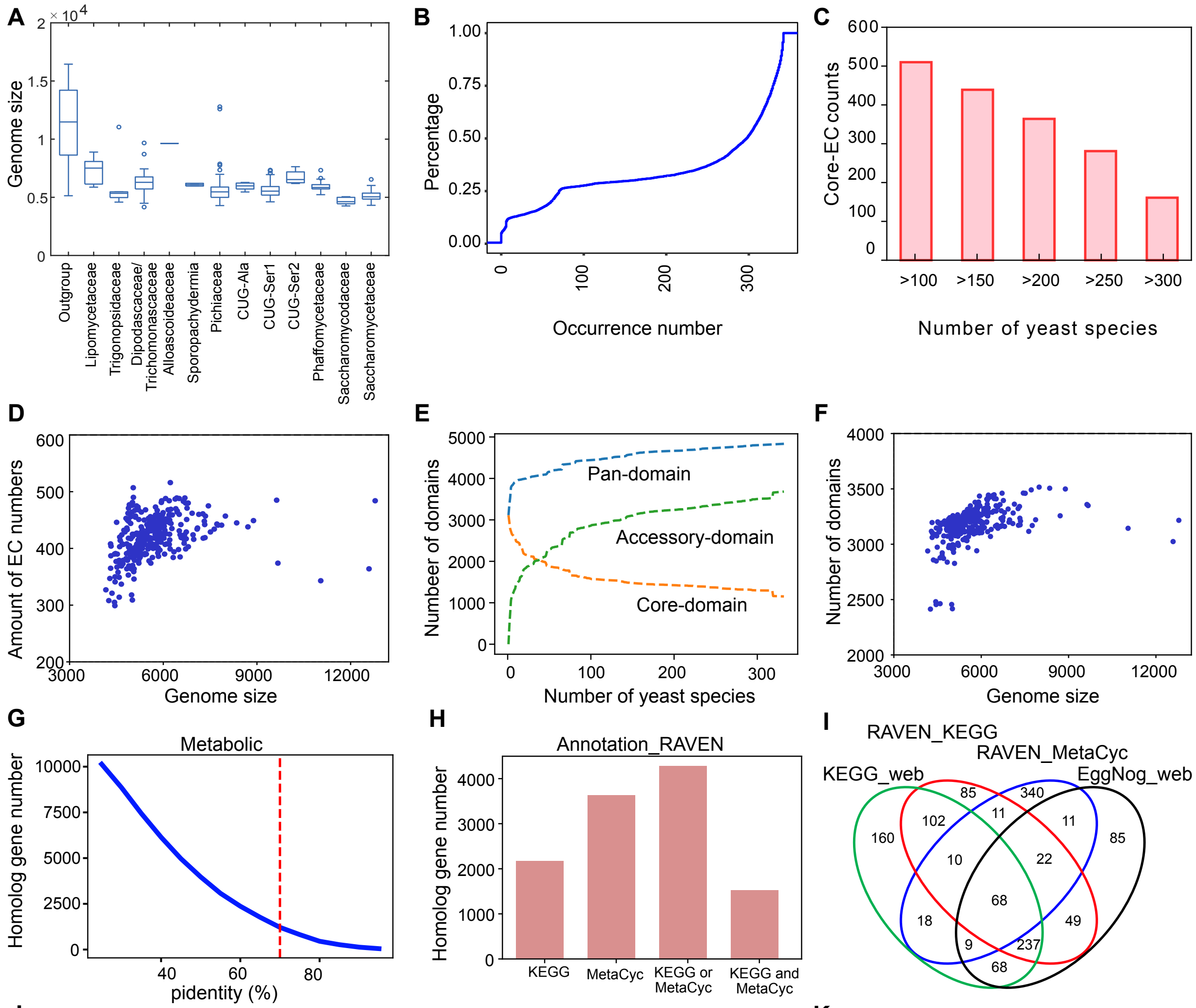

J

Items	pan-GEM for 343 species of this work	pan-GEM for 33 fungal species
Rxns	4599	2766
Metabolite with compartment	3135	1404
Orthologs	3751	1547
Compartment	14	10

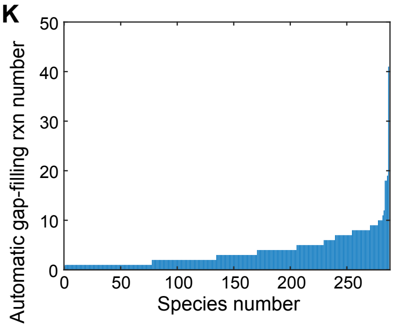

Appendix Figure S1 Genome evolution and annotation for 343 fungal species.

(A) Average genome size for each clade.

(B) Conservation analysis of *S. cerevisiae* genes across yeast subphylum.

(C) Core EC number analysis across yeast species. High-throughput prediction of enzyme commission (EC) numbers for all protein sequences from the 332 yeast species was implemented by DeepEC[1].

(D) Correlation between the genome size and predicted EC number for all yeast species.

(E) Domain analysis of all yeast species. Domain information was annotated for all proteins from the 332 yeast species, using the Pfam v32.0 database[2] (https://pfam.xfam.org) and Hidden Markov Models (HMMs)[3]. Pan-domains, core-domains (existing in all species) and accessory-domains (existing in part of species) were calculated respectively based on the predicted domains.

(F) Correlation between the genome size and domain number for all yeast species.

(G) Homolog gene numbers found by reciprocal best BLAST with pidenty as 70% using the *S. cerevisiae* S288c' genome as the reference.

(H) Homolog gene numbers based on the reaction annotation evidence from RAVEN2[4]. Here RAVEN_KEGG and RAVEN_MetaCyc represent new reactions obtained based on the methods defined in RAVEN2, which can obtain new reactions based on KEGG and MetaCyc databases respectively.

(I) Venn graph of new reactions from different annotation methods and there are 690 reactions from at least two methods.

(J) Comparison between pan-GEM in this work with a reported one[5].

(K) Statistical analysis of gap-filling reactions for all ssGEMs in this work.

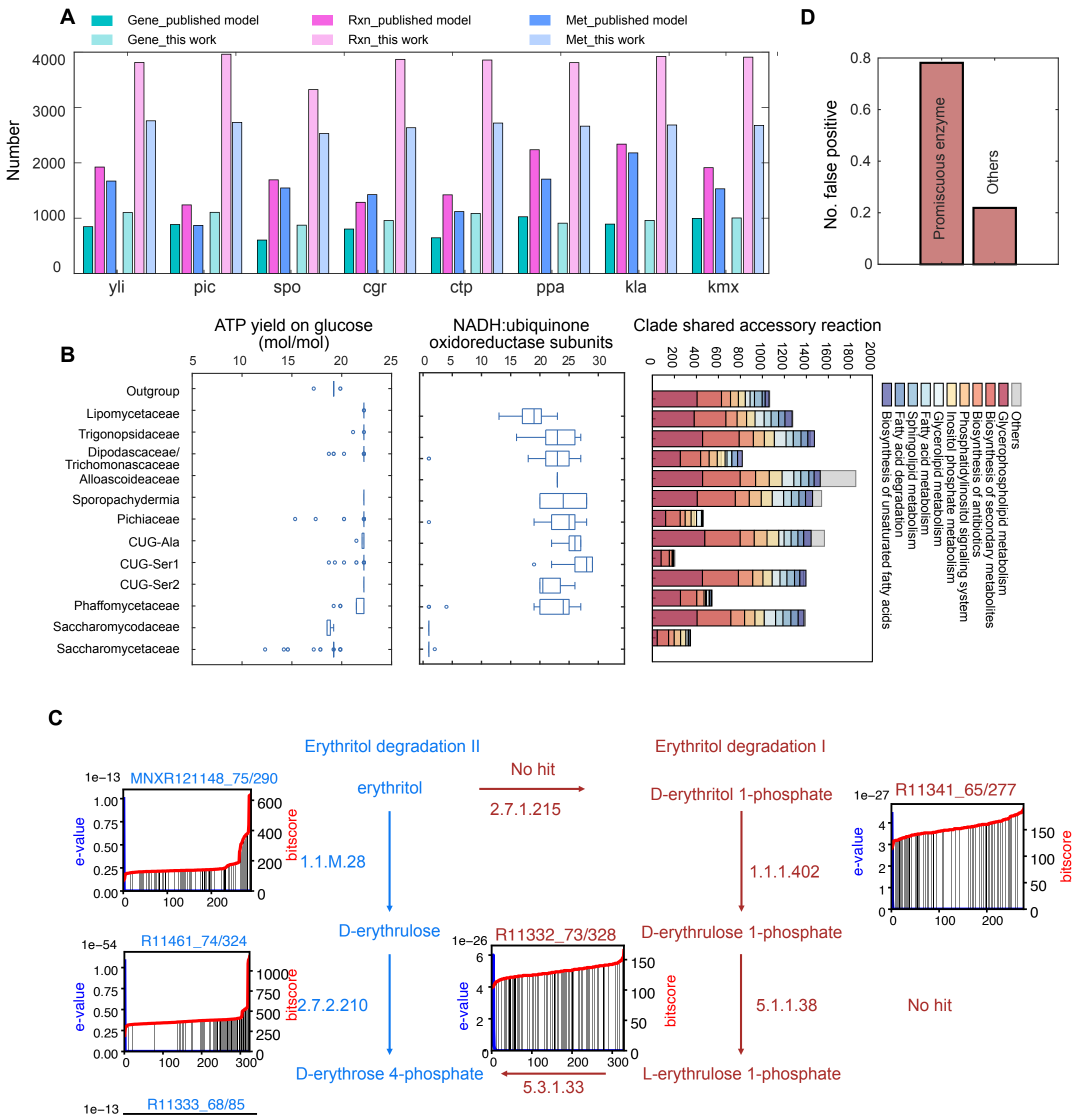

Appendix Figure S2 Evaluation of reconstructed ssGEMs in pathway mining and metabolic coverage.

(A) Comparison in metabolic coverage between ssGEMs from this study and published models. iYali4 for *Yarrowia lipolytica* (yli)[6], iNX804 for *Candida glabrata* (cgr)[7], iSM996 for *Kluyveromyces marxianus* (kmx)[8], iTL885 for *Scheffersomyces stipites* (pic)[9], SpoMBEL1693 for *Schizosaccharomyces pombe* (spo)[10], iCT646 for *Candida tropicalis* (ctp)[11], iMT1026.v3 for *Komagataella pastoris* (ppa)[12], iOD907 for *Kluyveromyces lactis* (kla)[13].

(B) Prediction of ATP yields with glucose as substrate; number of Complex I subunits present; and summary of pathways represented as accessory reactions in each clade.

(C) Gene mining for erythritol utilization. The red profiles represent the bitscore and the blue profiles represent the e-value during the BLAST analysis with sequences of known function as the references. Solid lines represent the yeast species used in this work with erythritol utilization phenotypes. The number in X axis represents the number of species with sequences identified by the BLAST analysis. The title for each figure stands for the reaction ID and the ratio stands for the overlap species number of experimental phenotype existence and species with presence of this reaction from the tblastn result / the number of species with presence of this reaction from the tblastn result.

(D) Possible factors affecting inconsistencies (false positive) between model simulation and experimental data. In case a model was able to catabolize a substrate in contrast to the experimental evidence, we first identified in the model which reactions and enzymes were responsible for the degradation of the particular substrate. If one of the enzymes involved was also annotated to other metabolic reactions in the model (i.e., promiscuous enzyme), then enzyme promiscuity was tallied as responsible for the false positive. Inclusion of reactions in ssGEMs are foremost guided by the presence of enzymes associated to those reactions. If a particular enzyme is promiscuous in pan-GEM (i.e., associated to multiple reactions with different substrates), then all these associated reactions will end up in ssGEMs that contain the enzyme. However, substrate selectivity might have changed during evolution of selected strains, such that the enzyme in specific strains might have lost or never gained the promiscuous activity that are otherwise assigned to it in pan-GEM, which likely results in inconsistence between the prediction and measured traits.

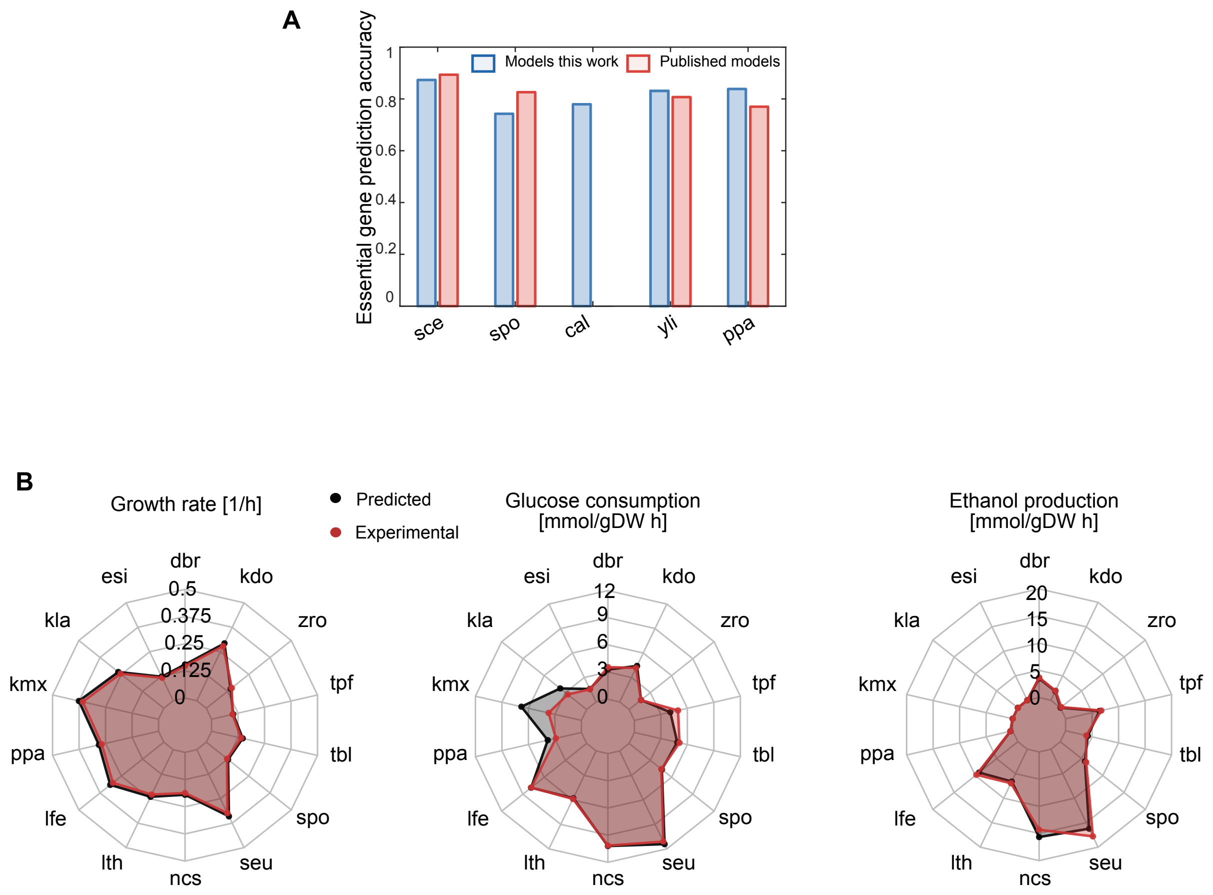

Appendix Figure S3 Prediction of essential genes and physiological data using ssGEMs and ecGEMs.

(A) Comparison in essential gene prediction accuracy between ssGEMs in this work and published GEMs. The prediction accuracies from published GEMs were determined in this work, except for the SpoMBEL1693 model of *S. pombe* which was instead as reported in[10], as we were unable to simulate the published model. No published model for *C. albicans* exists to date. sce: *S. cerevisiae*, spo: *S. pombe*, cal: *C. albicans*, yli: *Y. lipolytica*, ppa: *K. pastoris*.

(B) Phenotype prediction using ecGEMs for 14 yeast species after manual calibration. dbr: *Dekkera bruxellensis*; esi: *Eremothecium sinecaudum*; kla: *Kluyveromyces lactis*; kmx: *Kluyveromyces marxianus*; ppa: *Komagataella pastoris*; lfe: *Lachancea fermentati*; lth: *Lachancea thermotolerans*; ncs: *Naumovozyma castellii*; seu: *Saccharomyces eubayanus*; spo: *Schizosaccharomyces pombe*; tbl: *Tetrapisispora blattae*; tpf: *Tetrapisispora phaffii*; zro: *Zygosaccharomyces rouxii*; kdo: *yHMPu5000034710 Kluyveromyces dobzhanskii*.

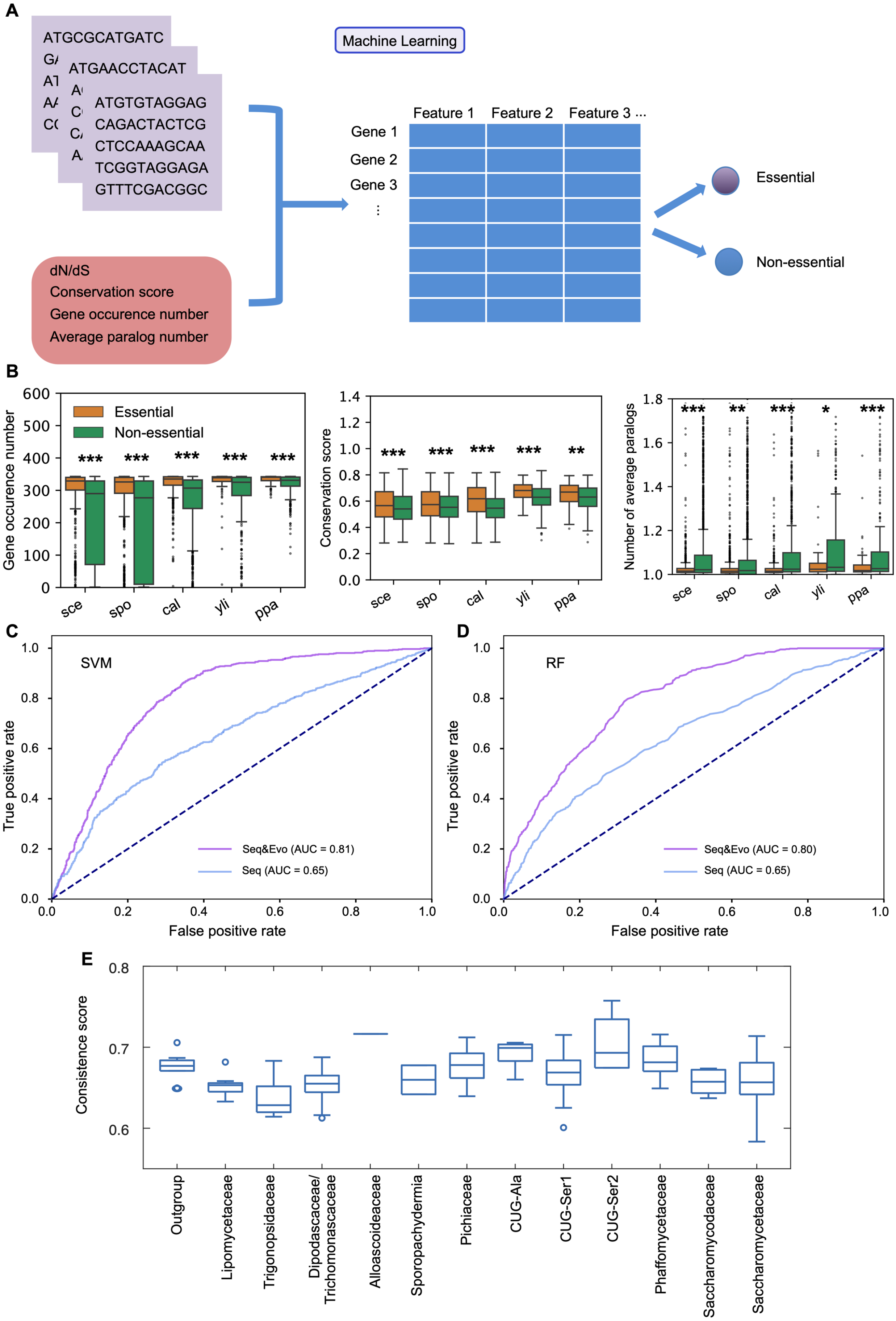
A
ATGCGCATGATC
ATGAACCTACAT
ATGTGTAGGAG
CAGACTACTCG
CTCCAAAGCAA
TCGGTAGGAGA
GTTTCGACGGC
dN/dS
Conservation score
Gene occurence number
Average paralog number
Machine Learning
Feature 1
Feature 2
Feature 3 ...
Gene 1
Gene 2
Gene 3
Essential
Non-essential
B
Gene occurence number
Essential
Non-essential
Conservation score
Number of average paralogs
sce
spo
cal
yli
ppa
C
SVM
True positive rate
False positive rate
Seq&Evo (AUC = 0.81)
Seq (AUC = 0.65)
D
RF
Seq&Evo (AUC = 0.80)
Seq (AUC = 0.65)
E
Consistence score
Outgroup
Lipomycetaceae
Trigonopsidaceae
Dipodascaceae/ Trichomonascaceae
Alloascoideaceae
Sporopachydermia
Pichiaceae
CUG-Ala
CUG-Ser1
CUG-Ser2
Phaffomycetaceae
Saccharomycodaceae
Saccharomycetaceae

Appendix Figure S4 Improved essential gene prediction guided by multi-dimensional evolutionary parameters.

(A) Schematic workflow for yeast essential gene prediction using machine learning methods.

(B) Evolution-based feature analysis between essential genes and non-essential genes across various yeast species. Symbols were used to indicate statistical significance, ∗: *P* value <= 0.05, ∗∗: *P* value <= 0.01, ∗∗∗: *P* value <= 0.001. sce: *S. cerevisiae*, spo: *S. pombe*, cal: *C. albicans*, yli: *Y. lipolytica*, ppa: *K. pastoris*. For the number of average paralogs, only the values in range of 1-1.8 were shown.

(C) Improved AUC value of the ROC curve for essential gene prediction on testing dataset using support vector machine (SVM) algorithm by adding evolutionary parameters.

(D) Improved AUC value of the ROC curve for essential gene prediction on testing dataset using random forest (RF) algorithm by adding evolutionary parameters.

(E) Comparison in essential gene prediction by ssGEMs and SVM for 338 yeast species without experimental evidence.

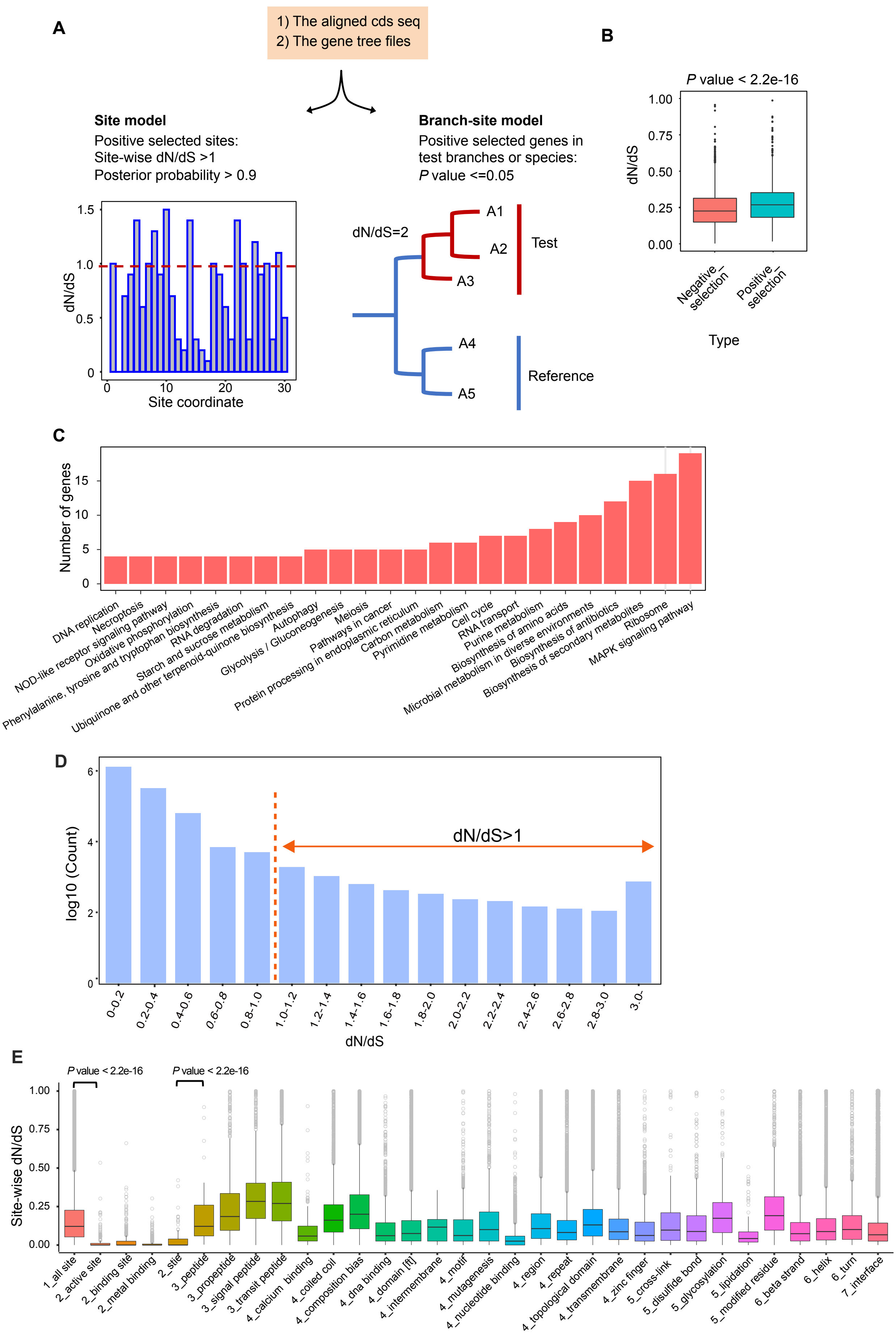

A
1) The aligned cds seq
2) The gene tree files
Site model
Positive selected sites:
Site-wise dN/dS >1
Posterior probability > 0.9
Branch-site model
Positive selected genes in test branches or species:
P value <=0.05
dN/dS
Site coordinate
dN/dS=2
A1
A2
A3
A4
A5
Test
Reference
B
P value < 2.2e-16
dN/dS
Negative_selection
Positive_selection
Type
C
Number of genes
DNA replication
Necroptosis
NOD-like receptor signaling pathway
Oxidative phosphorylation
Phenylalanine, tyrosine and tryptophan biosynthesis
RNA degradation
Starch and sucrose metabolism
Ubiquinone and other terpenoid-quinone biosynthesis
Autophagy
Glycolysis / Gluconeogenesis
Meiosis
Pathways in cancer
Protein processing in endoplasmic reticulum
Carbon metabolism
Pyrimidine metabolism
Cell cycle
RNA transport
Purine metabolism
Biosynthesis of amino acids
Microbial metabolism in diverse environments
Biosynthesis of antibiotics
Biosynthesis of secondary metabolites
Ribosome
MAPK signaling pathway
D
log10 (Count)
dN/dS>1
dN/dS
E
P value < 2.2e-16
P value < 2.2e-16
Site-wise dN/dS
1_all site
2_active site
2_binding site
2_metal binding
2_stie
3_peptide
3_propeptide
3_signal peptide
3_transit peptide
4_calcium binding
4_coiled coil
4_composition bias
4_dna binding
4_domain [ft]
4_intermembrane
4_motif
4_mutagenesis
4_nucleotide binding
4_region
4_repeat
4_topological domain
4_transmembrane
4_zinc finger
5_cross-link
5_disulfide bond
5_glycosylation
5_lipidation
5_modified residue
6_beta strand
6_helix
6_turn
7_interface

Appendix Figure S5 Evolution analysis in codon (amino acid residue) level.

(A) The procedures to do site model and "branch-site" model calculation in this work. The site model can identify which amino acid sites are positively selected across species while the "branch-site" model can identify which genes are significantly selected in specific lineages or branches of species.

(B) Comparison in gene level dN/dS for gene families (or OGs) with no positive selection and with at least one positive selected site.

(C) Pathway analysis of all gene families with positive selected sites.

(D) Distribution of all site-wise dN/dS.

(E) Statistical analysis of site-wise dN/dS based on the functional categories of residue sites defined in the UniProt database[14]. The grey dot represents the outlier points from each site category (here only the dN/dS between 0-1 was shown for comparison).

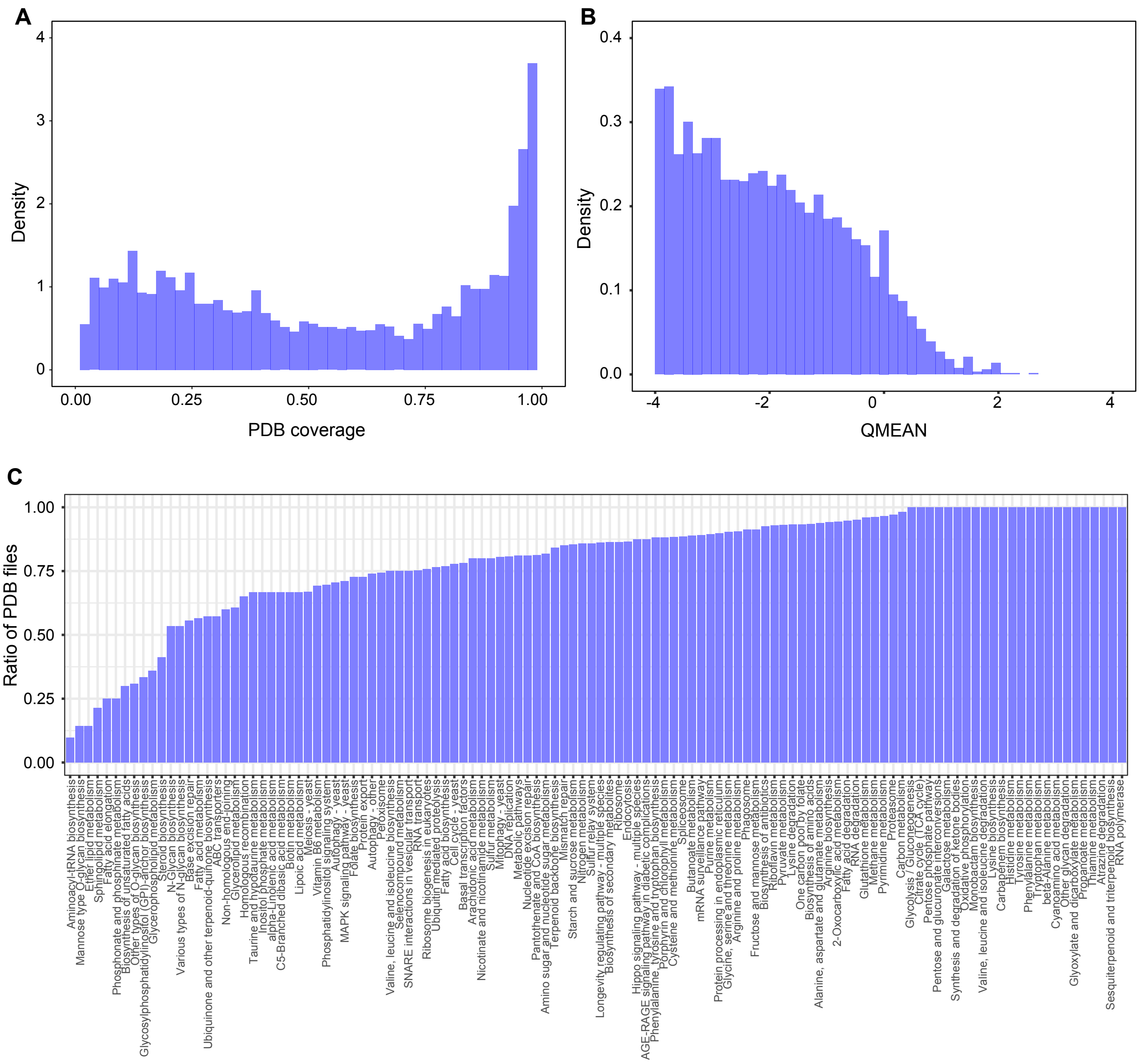

Appendix Figure S6 Statistical analysis of protein 3D structure for *S. cerevisiae* S288c used in this work.

(A) Statistical analysis of all PDB files in protein coverage percentages used in this work.

(B) Statistical analysis of homology PDB files with QMEAN $\geq$ -4.

(C) Ratios of proteins with 3D structures for *S. cerevisiae* from each sub-pathway.

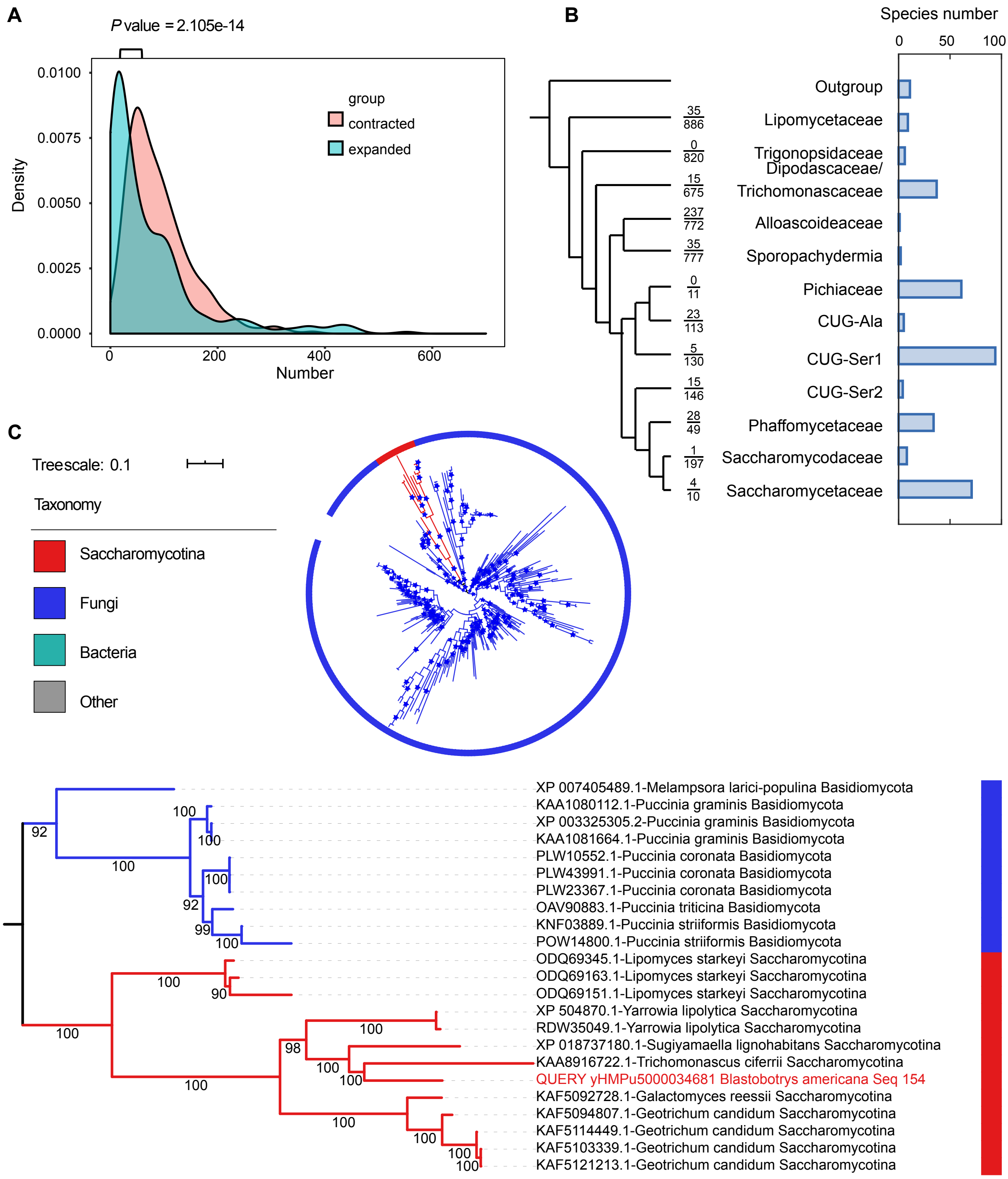

Appendix Figure S7 Gene family expansion and contraction analysis together with horizontal gene transfer analysis to illustrate the mechanism in the metabolic innovation occurred in yeasts.

(A) Comparison in numbers of expanded and contracted gene families in the species level.

(B) Numbers of expanded (upper number) and contracted gene families (bottom number) from each clade.

(C) An example of an HGT event from fungi to yeast. ML phylogeny of a protein - yHMPu5000034681_Blastobotrys_americana@Seq_154, which is related to D-arabinose utilization. Branches with bootstrap support higher than 80% are shown by star. The bottom phylogenetic tree represents pruned ML phylogenies depicting the phylogenetic relationship between this protein from the *Saccharomycotina* subphylum and their close relatives from other fungi.

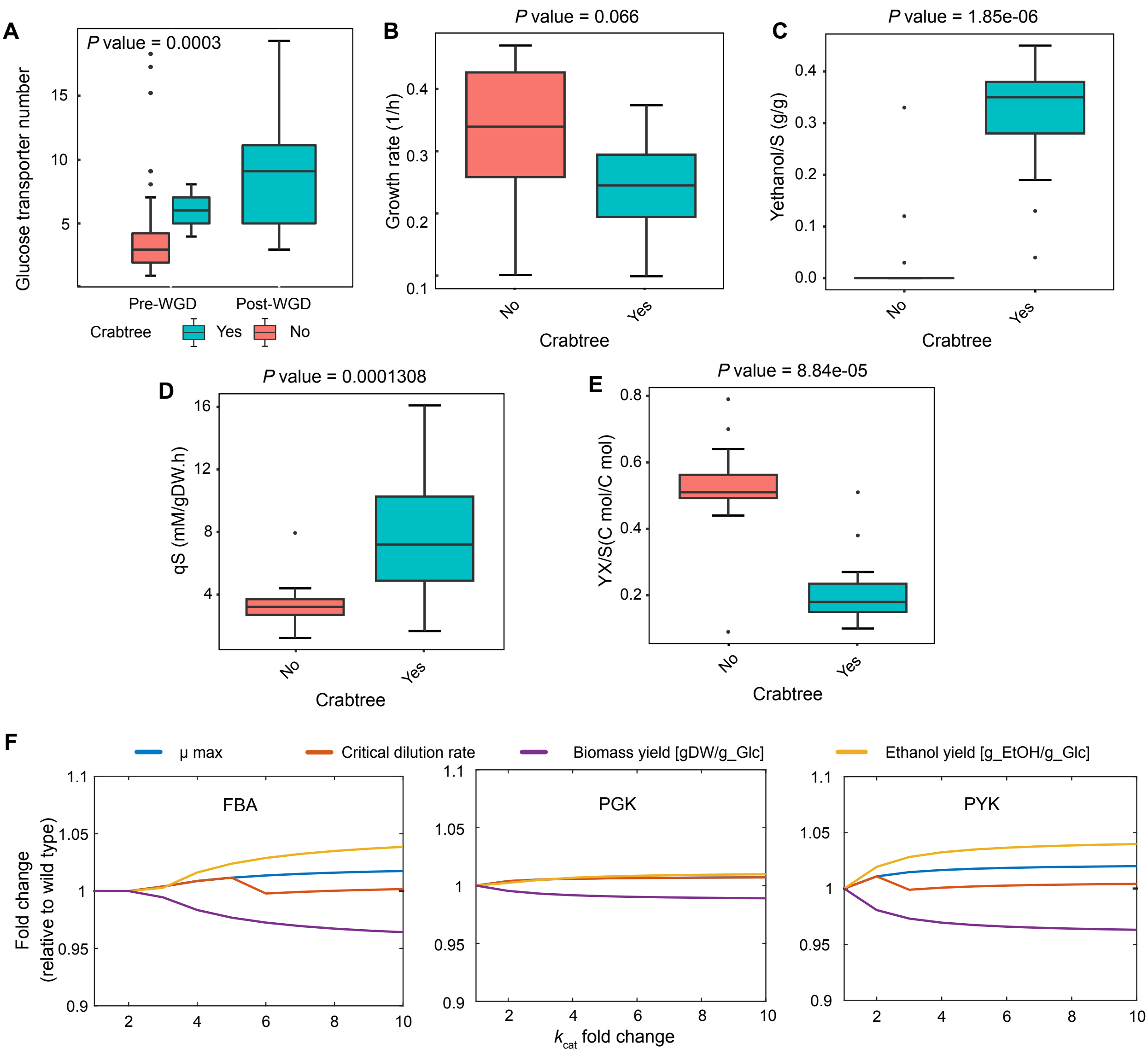

Appendix Figure S8 The mechanism for the generation of Crabtree effect in yeasts is further illustrated by the genetic data, physiological data and ecGEM simulation.

(A) The hexose transporter (HXT) copy numbers are significantly larger in Crabtree positive species than that in Crabtree negative ones.

(B) Comparison in specific growth rate between the Crabtree positive and negative species from literatures (summarized in Dataset EV2).

(C) Comparison in yield of ethanol relative to the glucose uptake rate between the Crabtree positive and negative species.

(D) Comparison in specific glucose uptake rate between the Crabtree positive and negative species.

(E) Comparison in yield of biomass relative to the glucose uptake rate between the Crabtree positive and negative species.

(F) Effects of fold changes in enzyme activity encoded by positively selected genes on Crabtree effect in *S. cerevisiae* using ecGEMs. From the simulation, it showed that increased k_{cat} in protein PGK, PYK and FBA could increase yield of ethanol and decrease yield of biomass.

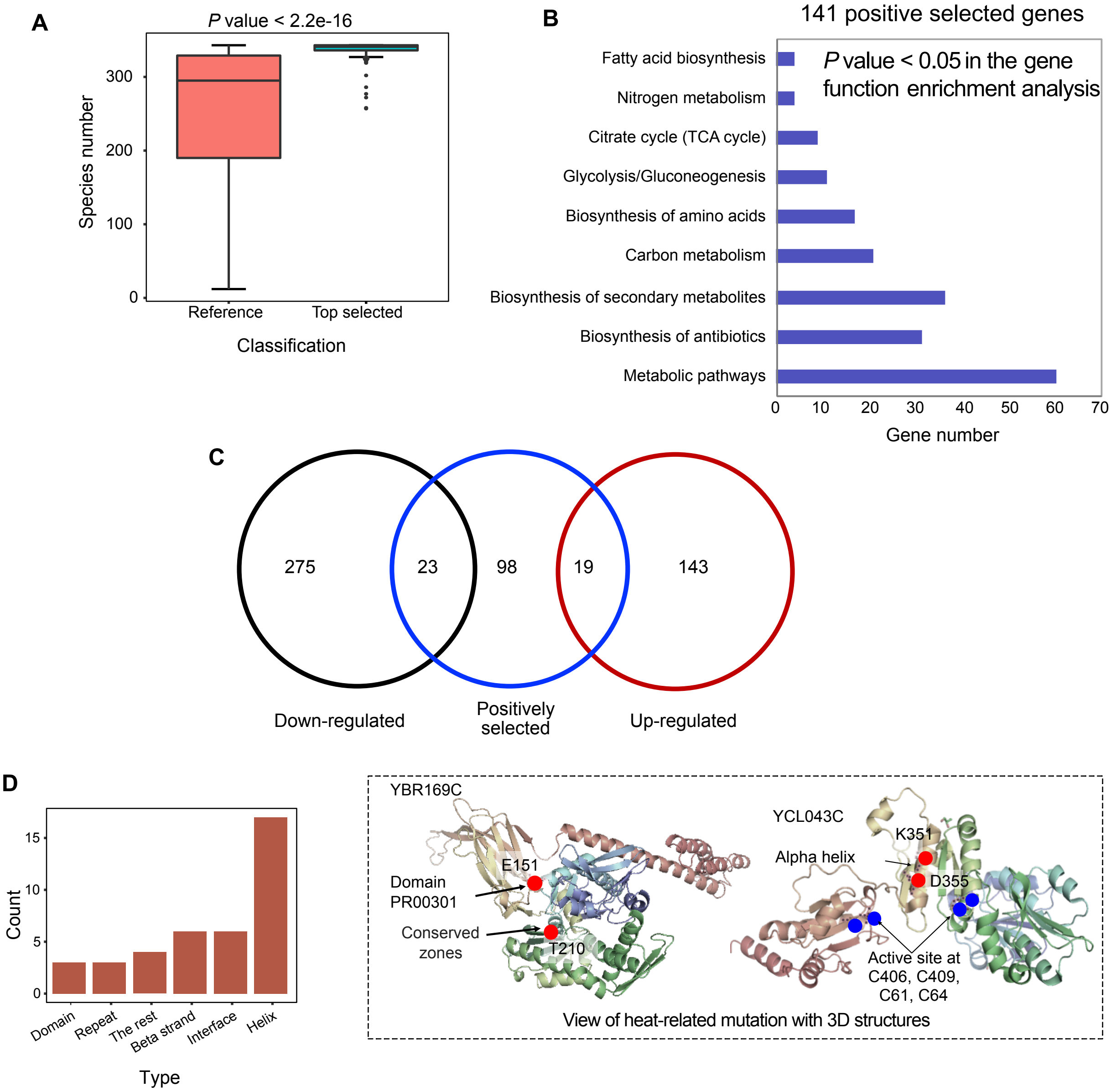

Appendix Figure S9 The mechanism for the generation of heat-tolerance in yeasts is further illustrated by the genetic data and proteomic data.

(A) Conservation analysis of the top positively selected genes and the reference genes across yeast species from 4812 ortholog groups used for branch-site model analysis related to the trait of "heat-tolerance".

(B) Distribution of positively selected genes related to the heat-tolerance in main sub-pathways.

(C) Comparison of up-regulated and down-regulated genes from proteomics study under higher temperature (38 °C vs 30 °C)[15] with the positively selected genes related to heat-tolerance in this work.

(D) The left bottom bar plot represents the statistical analysis in the functional annotation of the unique mutations contained in the above positively selected genes with the proteome annotation from *S. cerevisiae* S288c as the reference. In the right two protein 3D structures, the red circles represent the unique mutation related to the heat-tolerance while the blue circles represent the functional active site defined in *S. cerevisiae* S288c proteome from the UniProt database[14]. The 3D structure data was downloaded from the SWISS-model database[16].

Reference

1. Ryu, J.Y., Kim, H.U. & Lee, S.Y. Deep learning enables high-quality and high-throughput prediction of enzyme commission numbers. *Proc Natl Acad Sci U S A* **116**, 13996-14001 (2019).
2. El-Gebali, S. et al. The Pfam protein families database in 2019. *Nucleic Acids Res* **47**, D427-d432 (2019).
3. Shukla, S., Shamsi, Z., Moffett, A.S., Selvam, B. & Shukla, D. Application of Hidden Markov Models in Biomolecular Simulations. *Methods Mol Biol* **1552**, 29-41 (2017).
4. Wang, H. et al. RAVEN 2.0: A versatile toolbox for metabolic network reconstruction and a case study on *Streptomyces coelicolor*. *PLoS Comput Biol* **14**, e1006541 (2018).
5. Correia, K. & Mahadevan, R. Pan-Genome-Scale Network Reconstruction: Harnessing Phylogenomics Increases the Quantity and Quality of Metabolic Models. *Biotechnol. J.* **15**, 1900519 (2020).
6. Kerkhoven, E.J., Pomraning, K.R., Baker, S.E. & Nielsen, J. Regulation of amino-acid metabolism controls flux to lipid accumulation in *Yarrowia lipolytica*. *npj Systems Biology and Applications* **2**, 16005 (2016).
7. Xu, N. et al. Reconstruction and analysis of the genome-scale metabolic network of Candida glabrata. *Mol Biosyst* **9**, 205-216 (2013).
8. Marcišauskas, S., Ji, B. & Nielsen, J. Reconstruction and analysis of a *Kluyveromyces marxianus* genome-scale metabolic model. *BMC Bioinformatics* **20**, 551 (2019).
9. Liu, T., Zou, W., Liu, L. & Chen, J. A constraint-based model of *Scheffersomyces stipitis* for improved ethanol production. *Biotechnology for Biofuels* **5**, 72 (2012).
10. Sohn, S.B., Kim, T.Y., Lee, J.H. & Lee, S.Y. Genome-scale metabolic model of the fission yeast *Schizosaccharomyces pombe* and the reconciliation of in silico/in vivo mutant growth. *BMC Systems Biology* **6**, 49 (2012).
11. Mishra, P. et al. Genome-scale metabolic modeling and in silico analysis of lipid accumulating yeast *Candida tropicalis* for dicarboxylic acid production. *Biotechnology and Bioengineering* **113**, 1993-2004 (2016).
12. Tomàs-Gamisans, M., Ferrer, P. & Albiol, J. Fine-tuning the *P. pastoris* iMT1026 genome-scale metabolic model for improved prediction of growth on methanol or glycerol as sole carbon sources. *Microbial Biotechnology* **11**, 224-237 (2018).
13. Dias, O., Pereira, R., Gombert, A.K., Ferreira, E.C. & Rocha, I. iOD907, the first genome-scale metabolic model for the milk yeast *Kluyveromyces lactis*. *Biotechnology Journal* **9**, 776-790 (2014).
14. The UniProt Consortium UniProt: the universal protein knowledgebase. *Nucleic Acids Research* **45**, 158-169 (2017).
15. Lahtvee, P.J. et al. Absolute Quantification of Protein and mRNA Abundances Demonstrate Variability in Gene-Specific Translation Efficiency in Yeast. *Cell Syst* **4**, 495-504 e495 (2017).
16. Waterhouse, A. et al. SWISS-MODEL: homology modelling of protein structures and complexes. *Nucleic Acids Res.* **46**, 296-303 (2018).
