# Supplementary material for: Yeast metabolic innovations emerged via expanded metabolic network and gene positive selection
Source: Mol Syst Biol. 2021 Oct 22;17(10):e10427. doi: 10.15252/msb.202110427 (PMC8532513; doi:10.15252/msb.202110427)
Supplement: Supplementary file 14 [file MSB-17-e10427-s012.pdf]

molecular systems biology

Yeast metabolic innovations emerged via expanded metabolic network and gene positive selection

Hongzhong Lu, Feiran Li, Le Yuan, Iván Domenzain, Rosemary Yu, Hao Wang, Gang Li, Yu Chen, Boyang Ji, Eduard J Kerkhoven, and Jens Nielsen
DOI: 10.15252/msb.202110427

Corresponding author(s): Jens Nielsen (nielsenj@chalmers.se)

Review Timeline:

Submission Date:	1st May 21
Editorial Decision:	11th Jun 21
Revision Received:	9th Sep 21
Editorial Decision:	29th Sep 21
Revision Received:	2nd Oct 21
Accepted:	4th Oct 21

Editor: Maria Polychronidou

Transaction Report:

(Note: With the exception of the correction of typographical or spelling errors that could be a source of ambiguity, letters and reports are not edited. Depending on transfer agreements, referee reports obtained elsewhere may or may not be included in this compilation. Referee reports are anonymous unless the Referee chooses to sign their reports.)

1st Editorial Decision 11th Jun 2021

Thank you again for submitting your work to Molecular Systems Biology. We have now heard back from the three referees who agreed to evaluate your study. As you will see below, the reviewers raise a series of concerns, which preclude the publication of your study in its current form.

Overall, the reviewers think that as it stands the study presents rather limited novel insights and seems somewhat preliminary. However, as the reviewers acknowledge that the presented genome-scale models and data may be relevant for the field, we have decided to offer you the chance to address the issues raised in a major revision. Without repeating all the points listed below, some of the more fundamental issues that need to be addressed are the following:

- Follow up experiments need to be performed to validate new predictions generated by the models. Reviewer #2 makes specific recommendations regarding this point.

- The conclusion that ssGEMs can improve genome annotations and refine existing metabolic models needs to be better supported.

- Several conclusions and evolutionary claims, including the proposed link between MAPKs and adaptation, the involvement of genes in the Crabtree effect, the link between PDI1 and thermotolerance, need to be better supported by additional analyses.

- The analyses revealing new biology (which need to be strengthened as mentioned above) should be clearly distinguished from those yielding results in agreement with existing model and therefore confirming the validity of the approaches used.

- The work needs to be better placed in the context of the existing literature.

All issues raised by the reviewers need to be satisfactorily addressed. As you may already know, our editorial policy allows in principle a single round of major revision. It is therefore essential to provide responses to the reviewers' comments that are as complete as possible. I understand that the required revisions are quite substantive. Feel free to contact me in case you would like to discuss in further detail any of the issues raised or if you would like to share your revision plan with me.

On a more editorial level, we would ask you to address the following points:

REFEREE REPORTS

Reviewer #1:

The study by Lu et all combines genome-scale metabolic models and evolutionary analyses of gene family and gene sequence evolution to understand the evolution of yeast metabolism. Although the study uses previously published genomic data and traits, it is impressive in its scale and scope. The key take-home message is that evolution of yeast metabolism involved gene duplication, enzyme promiscuity, and gene sequence changes (driven by positive selection), which is not particularly surprising. The authors make several interesting observations but don't focus on any particular one, making it hard to fully evaluate the quality of their inferences. In any case, the genome-scale metabolic models and data generated by the study will likely be very useful to the yeast community but most of the study's findings seem confirmatory of what is already shown in several previous studies.

The authors frequently overstate the case (a few of the many examples are provided in the minor comments) or make conflicting statements - for example, in the abstract one reads "positive selection is the main driving force underlying the metabolic innovations in yeast subphylum", whereas the Discussion section is much more nuanced and suggests that gene duplication plays a major role. The authors seem to imply that these processes (gene duplication, enzyme promiscuity, gene sequence evolution, etc.) act independently of each other, but of course enzyme promiscuity can potentially favor the retention of gene duplicates and gene sequence evolution can influence the evolution of paralogs with different functions.

The methods used by the authors (at least the evolutionary ones) are ones that give results quickly (understandably given the size of the dataset) but are also known to have reduced accuracy (e.g., FastTree is used for tree-building but its low accuracy is well known, the pairwise yn00 model is used for dn/ds analysis but is of lower quality and accuracy than the M models, branch-site tests for dn/ds which are notorious for their false positive rates, etc.). I would have preferred fewer analyses with more robust methods, but at least a statement that these methods are not always accurate is warranted here.

I also think that the authors presentation of the results does not give sufficient credit to previous work. I provide several examples in the minor comments.

In summary, while i am impressed by the authors versatility of analyses, i struggled with evaluating their quality or figuring out what the main focus of the study is.

Minor comments:

line 22: yeasts certainly have diversity in their metabolic traits, but this diversity is found at various taxonomic levels. Seems strange to specifically single out "species-specific metabolic traits"

line 23: "how these metabolic traits have evolved remains elusive" - well, there have certainly been several studies (e.g., Riley et al 2016 PNAS, Shen et al 2018 Cell) that have specifically focused on this question.

lines 25-28: i don't understand this sentence and what you are trying to say - please revise.

Abstract: you use the word "multi-dimensional" but this is never defined.

line 50: there are MANY studies that have moved well beyond correlation and have validated the connection between genes and metabolic traits (e.g., the work of the Goncalves lab in Portugal and the work of the Hittinger lab in the US come to mind, not to mention the classic work by Sandy Johnson's group). Couple of example papers: https://pubmed.ncbi.nlm.nih.gov/32061177/and https://pubmed.ncbi.nlm.nih.gov/30297402.

line 100: reference 13 is based on work on prokaryotes and not on eukaryotes (as implied from the sentence).

line 234: what do you mean by "multiple levels"? You've only shown positive selection on some metabolic genes so far

line 281: "to gain these fitness phenotypes". This is unclear

lines 385-403: the inference that HGT is low in budding yeasts has previously shown in several studies (e.g., https://pubmed.ncbi.nlm.nih.gov/19969385/, https://pubmed.ncbi.nlm.nih.gov/25474404/, https://pubmed.ncbi.nlm.nih.gov/29648535/, https://pubmed.ncbi.nlm.nih.gov/30415838/), although some lineages (particularly the W/S clade) have much higher rates and other lineages (particularly the clades where the CUG codon has been reassigned) have very low rates.

Reviewer #2:

In the current study, Lu et al use genome-scale metabolic models coupled with information about protein function and yeast physiology to explore the evolution of metabolic innovation across yeasts. Main findings include identification of genes under positive selection across yeasts; development of methods to integrate evolutionary information to identify essential genes; identification of the spatial distribution of positively selected amino acid residues within proteins; discovery of the relatively small contribution of horizontal gene transfer (HGT) made to yeast substrate evolution; and unveiling of proteins potentially contributing to complex metabolic traits such as the Crabtree effect and thermotolerance. Overall, the study contains an impressive amount of data and is large in scope. The study is well-conducted and presents intriguing methods with implications for yeast biology, evolution, and metabolic modeling.

However, the study would benefit from further revisions to both writing and experimentation. In particular, the authors make several claims to discover new phenomena using in silico results that are not supported by experimental data, whereas other sections supported by existing data do not reveal significantly novel insights (rather, they serve to confirm the reliability of the authors' methods). Thus we recommend performing additional experiments where possible, and/or a revision of the language to more properly reflect the degree of novelty and certainty of some findings; specific examples are provided below. Altogether, these revisions would help the reader better understand this massive study and also provide critical support for claims about organismal biology made in the paper.

Line 102-110:
1. The authors state that ssGEM can improve genome annotations and refine existing metabolic models - where is the evidence for this? Please elaborate. If the improved annotation and refinement of existing metabolic models is truly a main takeaway of this paper, the authors should present and discuss evidence for this in more detail.

2. What does "under-determined" mean? It is unclear what this means, where the data are to support this, and how erythriol metabolism fits into this.

3. Regarding erythriol metabolism, in 68 out of 85 species where erythriol pathway II was detected there was known utilization. But clearly 17 species have the pathway but do not use Erythriol. Is this due to a lack of experimental evidence? Explain the discrepancy. Also, it is clear from Supplementary Figure 2a, that the identity between sequences of interest and known Erythriol-degrading enzymes is highly variable between species, how do you determine presence/absence? A % ID below 50% may well mean that the homolog does not metabolize the molecule of interest.

Fig 2a, 2c, LINE 127: the authors describe predictions in metabolic flux distribution as well as in silico calculations of biomass, ATP, and amino acid yields. However, if the authors wish to make claims such as "here we discovered that the Saccharomycodaceae and Saccharomycetaceae clades have a lower biomass and ATP yield", we recommend testing and validating some of these yield calculations experimentally in order to further demonstrate the reliability of these in silico calculations and predictions.
LINE 147: Similar to the above comment. While the accuracy of ecGEM predictions was verified with existing data on 14 yeast species, it is still worth revising the language to emphasize that the FCC values were calculated in silico. For example in LINE 171, "high predicted FCCs".

Line 137. The authors mention, referring to Figure 2d, that there is a phylogenetically based clustering of the clades based on their metabolic models. However, the authors use tSNE to represent these data, which does not present phylogenetic information. The authors should consider how to visually represent the clustering based on the metabolic modeling together with phylogenetic information, such as a phylogenetic tree - can the authors show visualization of real phylogeny vs metabolic modeling clustering in some way?

Figure 3b: There appears to be a statistically significant comparison between the dN/dS boxplots for "species number 250-300, accessory metabolic" and "species number 300-343, accessory metabolic". This does not seem to be something that can be appropriately compared, given that the two boxplots fall under different "species number" categories. Furthermore, the observation that the core, more conserved metabolic pathway genes, evolve more slowly and have lower dN/dS, than the accessories pathway genes. This does not seem like a novel observation, but serves to validate the methods applied.
Line 204-207. The authors mention that a number of genes within PSSs belong to the MAPK signaling pathway. The authors state that this indicates that positive selection could be beneficial to yeast in adapting to environment. This is a dubious claim with no clear evidence. If the authors want to establish a link between the presence of MAPK within PSSs and adaptation, they need to consider alternatives (eg a null hypothesis). For example, are MAPK overrepresented among PSSs, compared to other gene families? Are particular MAPK genes overrepresented among PSSs compared to non-PSSs?
Line 213: same as with Figure 3b, it does not seem very novel that the active and binding sites of enzymes have a lower dN/dS compared to the rest of the enzyme. While the observation that most positively selected or fast-evolved sites are generally >10 angstroms away from active or binding sites and that such sites don't cluster is a little more interesting, these results are still unsurprising. It is expected that such important sites of enzymes are highly conserved. As the authors conclude, these results primarily "confirm" that residues are subject to different pressures depending on function and localization.

Figure 4c, it will be interesting/important to compare the exact same categories here as in Figure 3c. Both investigate similar concepts, but for some reason categories are left out of 4C/extra categories are included in 3C.

Line 242, also relevant to Figure 5: there is clear variability in HGT between clades. Could the authors discuss this? What explains this variability? Most clades have almost zero HGT, while a few have visibly more.

Line 274 - when discussing ethanol utilization in Hansensiaspora, the authors state that their prediction is in good agreement with experimentally determined substrate utilization in these species. However, there is no reference. Could the authors identify a reference and expand on this statement?

Line 300, Figure 6. URA1 has been duplicated many times in the outgroup of fungi, but only one is Crabtree positive. How do the authors explain this in light of their claim that URA1 is important for emergence of Crabtree effect?

Line 305-309: The authors mention that there was positive selection for genes in the EMP pathway among four independent lineages of Crabtree-positive yeasts. To establish the significance of this positive selection, the authors need to compare to a null hypothesis. Does this positive selection also occur in the outgroup of non-Crabtree fungi? Does it occur at all in non-Crabtree yeasts? The presence alone of positive selection does not prove involvement of these genes in the Crabtree effect.

Line 311-315. The authors invoke circumstantial evidence about FBA1, PGK and PYK, to explain the evolution of the Crabtree effect across clades. This lacks experimental evidence. The authors should consider removing these claims or providing experimental evidence. At the very least, similar to above, does this positive selection also occur in the outgroup of non-Crabtree fungi? Does it occur at all in non-Crabtree yeasts?

Line 333-337: The authors describe that some positively selected genes were upregulated in previous experiments in Baker's yeast. However, just because 42 of positively selected genes (related to thermotolerance) were upregulated at 38C vs 30C in yeast does not mean that they contribute to thermotolerance; if the authors want to use this argument, they also need to consider how many of not positively selected genes were upregulated at 38C. These positively selected proteins could reflect a general stress response rather than actually contributing directly to the phenotype. The evidence presented in lines 338-348 is more intriguing, although it also presents circumstantial evidence without experimental verification.

Overall, regarding the discussion of the Crabtree effect and Thermotolerance, many claims are made about the factors that may contribute to these phenotypes across yeasts. However, the claims about organismal biology are not supported by the data of the current study. The authors present an intriguing overview of the distribution of these phenotypes across yeasts, and also present a survey of interesting candidate genes/traits that correlate with these phenotypes. Yet, given the results of the study, it still remains unknown whether the methods presented by the authors can truly predict the evolution of complex metabolic traits. If the authors want to make claims about mechanisms and involvement of specific players, more experimental validation is needed. For example, regarding thermotolerance, in Figure 6, the authors could experimentally validate prediction of thermotolerance in the disulfide isomerase presented as a specific example (does this protein have altered Tm if the residues of interest are changed?). Scaling back the claims in this section would not compromise the novelty or contribution of the overall study. In fact, given the vast amount of data and the large scope of the study, the authors may be best served scaling back (or relegating to the SI) the data/discussion of Figure 6, which would provide a more succinct and well-supported message to the reader.

A general comment: many figure captions lack information, making the figures difficult to understand. The authors should provide more experimental information and explanation for figures throughout the paper. We provide examples below with the understanding that the authors should look the entire paper and consider changes:

Figure 1a caption needs more information/explanation as it is unclear what various panels represent. This is especially important as this Figure sets up the methods and concepts used throughout the paper.

Supplementary Figure 2C - how do the authors determine the cause of inconsistencies? How do they determine promiscuous enzymes?

Supplementary Figure 3a. Unclear what abbreviations mean. This comment is relevant throughout figures where abbreviations for species names are used.

Figure 2e - what are the horizontal labels on the heatmap?

Figure 3b - why are species grouped in the following way by number? The analysis is unclear. The significance of the statistical test of the species grouping is unclear.

Figure 4c. Box plot is unclear. Unclear from caption what points and scale represent.

Figure 6g and 6h. It is completely unclear what these figures represent and their relevance to the current study.

Reviewer #3:

In this study, Lu and coworkers study the evolution and divergence of yeast metabolic networks. In short, single-species metabolic networks, as well as one pan-genomic network are generated and analyzed. Perhaps not surprisingly, the results show strong conservation of core metabolism and active sites, and more evolutionary divergence for the peripheral networks and peripheral residues, with promiscuous enzymes playing a central role in evolutionary innovation. The strength of this study then perhaps does not lie in the generation of novel evolutionary concepts, but rather it providing an unprecedented bird's eye view on yeast metabolic networks and their evolutionary divergence. As such, it is a very interesting piece of work that provides a great overview and which will serve as a resource for future research.

Overall, the study is solid and the text is very well written and clear. My main remark is that some evolutionary claims are perhaps not fully supported by data. As such, I only have a few relatively minor comments and suggestions to add some nuances.

1. A lot of attention is given to dN/dS analysis to find signs of positive and negative selection. However, this is a rather rough estimate of selection that has several limitations. It would be good to evaluate the results more critically. In particular, I wonder whether many of the higher dN/dS figures found for peripheral residues are reflecting a lack of negative (purifying) selection, rather than true positive selection? In that sense, focusing on residues that are not neutral and bound to have an effect (such as those located in an active site) and looking for smaller deviations in dN/dS could provide more valuable insight; and/or finding examples of parallel evolution. In addition, it would be good to dive a bit deeper into some examples, and to be a bit more cautious with drawing overly strong conclusions (eg lines 379).
2. Some aspects are perhaps a bit underdeveloped, such as for example the role of promiscuous enzymes. Is it possible to provide more in-depth analyses there? The archetypal evolutionary model is that evolutionary innovation happens through duplication and subsequent divergence of promiscuous enzymes, allowing a side-activity to develop further and become much higher in one of the paralogs. For example, the residues that provide specificity or promiscuity in maltose-degrading enzymes have been relatively well studied (and there must be more examples), and this information could be mapped onto the networks to link it to copy numbers, residues and phenotypes.
3. Whereas I do see the correlation between selection for certain EMP genes and emergence of the Crabtree effect; but I am less convinced that you can write that the emergence of the Crabtree effect benefitted from selection on the genes; it seems difficult to establish a direct causal relationship here (did the flux first change, stimulating the Crabtree effect, or did the Crabtree effect evolve and then cause more positive selecton on the EMP, or -perhaps most likely- are both events simply intertwined, likely also with many other events? I think that this is also the theory of the authors, but some of the sentences (eg line 406-407) miss that nuance.
4. I must admit being confused about the proposed link between the EMP and temperature tolerance. While the link between the EMP and the Crabtree effect is also not crystal clear (see above), the link is quite believable and obvious. As far as I can judge, this is less the case for EMP and temperature tolerance; so I would again advise to either provide more evidence (which could entail making mutations in some of the enzymes and studying their stability and activity at various temperatures; as well as studying the effect of expressing combinations of alleles on the temperature tolerance of a given yeast species), or soften the claims a bit.

Typo line 414 (,,)

best wishes-
Kevin Verstrepen

REFEREE REPORTS

Reviewer #1:

The study by Lu et all combines genome-scale metabolic models and evolutionary analyses of gene family and gene sequence evolution to understand the evolution of yeast metabolism. Although the study uses previously published genomic data and traits, it is impressive in its scale and scope. The key take-home message is that evolution of yeast metabolism involved gene duplication, enzyme promiscuity, and gene sequence changes (driven by positive selection), which is not particularly surprising. The authors make several interesting observations but don't focus on any particular one, making it hard to fully evaluate the quality of their inferences. In any case, the genome-scale metabolic models and data generated by the study will likely be very useful to the yeast community but most of the study's findings seem confirmatory of what is already shown in several previous studies.
Response: We thanks the reviewer for their kind and constructive comments. In the revised manuscript we have improved the focus by, among other things, removing some content, rephrasing text and clarifying the figure captions. We are further more explicit on what existing experimental data and findings are supporting our analyses

We believe that our study represents progress over previous studies, according to the following points. 1) We took numerous efforts to build and test genome-scale metabolic models (GEMs) for 332 yeast species (plus 11 fungal species) and these models itself provide new chances to explore the complex relationships between genotype and phenotype, which will be a valuable resource for the yeast community. We think that it will be quite difficult to do some analysis without model information. For example, the gene loss in the distant pathways will also result in the trait loss. The integrative analysis on top of metabolic model is actually one key point of our work. 2) We tried to combine metabolic model simulation with traditional evolution analysis. With such an integrative analysis, we want to reveal

some consistent clues (supported by gene selection analysis, model prediction and experimental evidences) underlying the formation of complex traits (here we mainly focused on thermotolerance). To our best, we think this is the first time of large-scale systematic evolution analysis with aid of metabolic model reconstruction and simulation to find polygenetic and multiscale features relevant for the generation of important traits across 332 different yeast species. We also agree with the idea of Review 3# that our work will be a good resource for more in-depth analysis by the research community as it shows how our integrative procedure could provide some interesting findings to uncover the correlation between genotype and cellular fitness of yeast.

The authors frequently overstate the case (a few of the many examples are provided in the minor comments) or make conflicting statements - for example, in the abstract one reads "positive selection is the main driving force underlying the metabolic innovations in yeast subphylum", whereas the Discussion section is much more nuanced and suggests that gene duplication plays a major role. The authors seem to imply that these processes (gene duplication, enzyme promiscuity, gene sequence evolution, etc.) act independently of each other, but of course enzyme promiscuity can potentially favor the retention of gene duplicates and gene sequence evolution can influence the evolution of paralogs with different functions.
Response: Thanks for your nice comments. Now we removed the sentence "positive selection is the main driving force underlying the metabolic innovations in yeast subphylum" and rephased the summary at the end of our introduction part.

The methods used by the authors (at least the evolutionary ones) are ones that give results quickly (understandably given the size of the dataset) but are also known to have reduced accuracy (e.g., FastTree is used for tree-building but its low accuracy is well known, the pairwise yn00 model is used for dn/ds analysis but is of lower quality and accuracy than the M models, branch-site tests for dn/ds which are notorious for their false positive rates, etc.). I would have preferred fewer analyses with more robust methods, but at least a statement that these methods are not always accurate is warranted here.
Response: We agree with your comments that the toolboxes we pursue might sacrifice some quality. To balance the speed and accuracy, different strategies were used in our analysis. For instance, in the tree reconstruction, FastTree is one of the optimal tools[1] and thus used, while in validation of horizontal gene transfer (HGT) analysis, phylogenetic trees were built using IQ-TREE v1.6.12[2] with 1000 ultrafast bootstrapping replicates[3]. It should be noted that in nearly all the analysis, we usually integrate different layers of dataset together to do the integrative analysis to reduce potential bias in our conclusion. We also verified our analysis with past work from the literature as much as possible. In some cases, to reduce bias in the evolution analysis, we employed different toolboxes (like the calculation in the site model tests), as well as the duplicated calculations (like the calculation in the 'branch-site' model tests). To address this we added a statement that these methods are not always accurate in the method part.

I also think that the authors presentation of the results does not give sufficient credit to previous work. I provide several examples in the minor comments.
Response: We have added more citation and relevant discussion based on your nice suggestion to the introduction.

In summary, while i am impressed by the authors versatility of analyses, i struggled with evaluating their quality or figuring out what the main focus of the study is.
Response: Here, we tried to make our story more focused by redesigning Figure 1, 3, 4 & 6. For example, in the analysis of the Crabtree effect, we mainly focus on our analysis in the gene positive selection and model simulation while we remove the other analysis which can be found in previous studies. Meanwhile, we increased the content in showing how GEM reconstructions help to refine gene function annotation and how evolution at residue site level can be associated with trait variance. We also added more experimental evidences to verify the result from model simulation and evolution analysis of complex traits based on comments from Reviewer 2. Our focus is thus: “With integrated analysis from modelling simulation and traditional evolution analysis, we could in-depth uncover how evolution shape the trait diversity of yeast”. To summarize, we believe that we provide three main contributions. 1) We build GEMs for 332 yeast species, which will be a good start to reconstruct GEMs of high-quality for various non-model yeast species. 2) With GEMs, we explore the mechanism underlying the trait diversity in substrate utilization. 3) With integrative analysis from model prediction and systematic evolution analysis, we show that polygenetic and multiscale features are relevant for the emergence of complex traits (focusing on thermotolerance) in yeast. We hope that the refined storyline and the new figure design in the revised version will make our work more focused, clearer and easier to be understood.

Minor comments:

line 22: yeasts certainly have diversity in their metabolic traits, but this diversity is found at various taxonomic levels. Seems strange to specifically single out "species-specific metabolic traits"
Response: We have removed “species-specific”.

line 23: "how these metabolic traits have evolved remains elusive" - well, there have certainly been several studies (e.g., Riley et al 2016 PNAS, Shen et al 2018 Cell) that have specifically focused on this question.
Response: We appreciate the extensive and valuable work that has been done on this topic and, accordingly, we have therefore rephrased this sentence to put forward the unresolved scientific issue we want to explore.

lines 25-28: i don't understand this sentence and what you are trying to say - please revise.
Response: We have revised this sentence as - “These GEMs were able to comprehensively characterize trait diversity and predict enzyme functionality, thereby signifying that sequence level evolution has shaped reaction networks towards new metabolic functions”.

Abstract: you use the word "multi-dimensional" but this is never defined.
Response: The word “multi-dimensional” is now defined in the first paragraph of the result section. In the new version of the abstract, we remove this word and rephrased it as “We performed integrative evolution analysis to investigate how genomic evolution determines trait generation by reconstructing genome-scale metabolic models (GEMs) for 332 yeasts.”

line 50: there are MANY studies that have moved well beyond correlation and have validated the connection between genes and metabolic traits (e.g., the work of the Goncalves lab in Portugal and the work of the Hittinger lab in the US come to mind, not to mention the classic work by Sandy Johnson's group). Couple of example papers: https://pubmed.ncbi.nlm.nih.gov/32061177/ and https://pubmed.ncbi.nlm.nih.gov/30297402.
Response: We have extended this section of the introduction by explicitly exemplifying studies where genome evolution has been mechanistically linked to metabolic traits.

line 100: reference 13 is based on work on prokaryotes and not on eukaryotes (as implied from the sentence).
Response: Thanks for pointing this out. We have changed the reference to [4], which shows adaptation of yeasts to their fruit habitat.

line 234: what do you mean by "multiple levels"? You've only shown positive selection on some metabolic genes so far
Response: With "multiple levels" we implied evolution not only on the level of SNPs, but also gene duplication, HGT, and even the effect this has on sub-pathway functioning, which is only addressed in the following section, so the previous phrasing was indeed unclear. "As ssGEMs were able to associate metabolic traits with genomic and site-specific diversity, we could further leverage these models to investigate *how* the trait diversity of yeast has arisen, by mapping evolutionary events onto metabolic pathways."

line 279: "to gain these fitness phenotypes". This is unclear
Response: We have simplified the sentence in the revised manuscript. Previously, it means" gene positive selection could increase the cellular fitness of one metabolic trait under specific niches".

lines 385-403: the inference that HGT is low in budding yeasts has previously shown in several studies (e.g., https://pubmed.ncbi.nlm.nih.gov/19969385/, https://pubmed.ncbi.nlm.nih.gov/25474404/, https://pubmed.ncbi.nlm.nih.gov/29648535/, https://pubmed.ncbi.nlm.nih.gov/30415838/), although some lineages (particularly the W/S clade) have much higher rates and other lineages (particularly the clades where the CUG codon has been reassigned) have very low rates.
Response: We appreciate this comment supporting our finding related to low HGT levels and we have now cited this work in the discussion. Nonetheless, our study goes further than concluding that HGT is low in budding yeasts, as we particularly implicate the role of different evolutionary mechanisms (including gene family expression, HGT and promiscuity) to substrate utilization diversity.

Reviewer #2:

In the current study, Lu et al use genome-scale metabolic models coupled with information about protein function and yeast physiology to explore the evolution of metabolic innovation across yeasts. Main findings include identification of genes under positive

selection across yeasts; development of methods to integrate evolutionary information to identify essential genes; identification of the spatial distribution of positively selected amino acid residues within proteins; discovery of the relatively small contribution of horizontal gene transfer (HGT) made to yeast substrate evolution; and unveiling of proteins potentially contributing to complex metabolic traits such as the Crabtree effect and thermotolerance. Overall, the study contains an impressive amount of data and is large in scope. The study is well-conducted and presents intriguing methods with implications for yeast biology, evolution, and metabolic modeling.

However, the study would benefit from further revisions to both writing and experimentation. In particular, the authors make several claims to discover new phenomena using in silico results that are not supported by experimental data, whereas other sections supported by existing data do not reveal significantly novel insights (rather, they serve to confirm the reliability of the authors' methods). Thus we recommend performing additional experiments where possible, and/or a revision of the language to more properly reflect the degree of novelty and certainty of some findings; specific examples are provided below. Altogether, these revisions would help the reader better understand this massive study and also provide critical support for claims about organismal biology made in the paper.
Response: We appreciate the constructive comments, which we have addressed by changes throughout the manuscript. In particular, we have improved the story line and focus by scaling back some of the claims and descriptions, especially related to Figure 6.

Line 102-110:
1. The authors state that ssGEM can improve genome annotations and refine existing metabolic models - where is the evidence for this? Please elaborate. If the improved annotation and refinement of existing metabolic models is truly a main takeaway of this paper, the authors should present and discuss evidence for this in more detail.
Response: We now realize that we indeed did not explicitly elaborate on this. For less-studied or newly sequenced species that are phylogenetically related to the pan-GEM, the ss-GEM reconstruction pipeline can quickly annotate enzymes in their genomes by assigning reactions and thereby reconstructing GEMs for those species. As an example of for GEMs of well-studied species, even while the *S. cerevisiae* GEM has been curated since 2003, it still contains reactions without associated genes. During our analyses we identified several genes associated to those reactions. For example, the gene CAB4 (YGR277C) should be associated with reaction r_1997, which is the intermediate step in the synthesis of CoA from pantothenate. We added gene associations for 14 reactions and expanded the gene coverage by adding 15 new genes, which improved the quality of *S. cerevisiae* GEM (Dataset EV2). We added more description on this in the revised paper.

2. What does "under-determined" mean? It is unclear what this means, where the data are to support this, and how erythriol metabolism fits into this.
Response: We have now extensively rephrased this section, to clarify that with “under-determined” we meant that the existence of particular pathways have previously not been fully elucidated for most yeast species. In particular, erythritol catabolism has two proposed pathways in MetaCyc and our ssGEM reconstruction showed that erythritol-catabolizing

yeasts have pathway II, as also shown in Fig S2 where no hits were found for two essential reactions of pathway I.

Erythritol catabolism has been mainly characterized in bacteria. As for yeast species, the reported data is poor. To the best of our knowledge, studies have only been conducted on two yeast species: *L. starkeyi*[5] and *Y. lipolytica*[6]. Erythritol degradation pathways I and II (Appendix Figure 2) have both been proposed as erythritol catabolic pathways in the Metacyc database. Here during the reconstruction of ssGEMs, we identified that erythritol degradation pathway II is more likely existing in yeast species by checking the existence of key enzymes which are correlated with the erythritol utilization phenotype.

3. Regarding erythriol metabolism, in 68 out of 85 species where erythriol pathway II was detected there was known utilization. But clearly 17 species have the pathway but do not use Erythriol. Is this due to a lack of experimental evidence? Explain the discrepancy. Also, it is clear from Supplementary Figure 2a, that the identity between sequences of interest and known Erythriol-degrading enzymes is highly variable between species, how do you determine presence/absence? A % ID below 50% may well mean that the homolog does not metabolize the molecule of interest.
Response: Of the remaining 17 species, no growth data was available for 11 species, while the remaining 6 species were experimentally shown not to be able to grow on erythritol. This discrepancy between presence of the pathway but inability to catabolize may be caused by other reasons such as regulation and low enzymatic activity.

To determine presence/absence, we previously used the same method used in the literature when they determined the existence of the enzyme for sugar utilization in yeast species, uses E-value as the cutoff. To ensure robustness of our results, we reran the analysis using bitscore >50, E-value <1e-10, coverage >70% and identity >30% as cutoffs, and only homologs satisfying all these four conditions were selected as present. The E-value cutoff is from this reference [7]. The identity and bitscore cutoff are from this reference[8]. We updated Appendix Figure 2C (we have adjusted the order of subfigures from Appendix Figure 2) based on this and added the above content in the revised manuscript.

Fig 2a, 2c, LINE 127: the authors describe predictions in metabolic flux distribution as well as in silico calculations of biomass, ATP, and amino acid yields. However, if the authors wish to make claims such as "here we discovered that the Saccharomycodaceae and Saccharomycetaceae clades have a lower biomass and ATP yield", we recommend testing and validating some of these yield calculations experimentally in order to further demonstrate the reliability of these in silico calculations and predictions.
Response: In order to further support the simulation results, we have decided to focus on the most divergent outcomes, i.e. the lower ATP and biomass yields of selected clades. As shown in the manuscript (Fig S2B), lower ATP and biomass yields coincide with an absence of complex I (NADH:ubiquinone oxidoreductase) of the electron transport chain. We have gathered experimental chemostat data from growth on glucose for various yeast species (Dataset EV2) and indeed found that presence of complex I gives relatively higher biomass yields (average 0.520 *vs.* 0.481, P value = 8.6271e-04, Wilcoxon rank sum test). Note that this includes only data at low growth rates at full respiration, to avoid the bias of fermentation pathways in Crabtree positive yeasts at high growth rate.

Further to this it has been reported that knock-out of complex I in *O. parapolymorpha* decrease biomass yield in chemostat cultivations [9]. These experimental data are consistent with our simulations and all these original datasets are now collected in Dataset EV2. However, note that the biomass yield is also influenced by the ATP requirement for growth such as the energy required for diverse biomass compositions and growth-associated maintenance energy, which may eliminate the advantage of the ATP production contributed by the complex I. Based on the above analysis, we revised the description in the manuscript accordingly.

LINE 147: Similar to the above comment. While the accuracy of ecGEM predictions was verified with existing data on 14 yeast species, it is still worth revising the language to emphasize that the FCC values were calculated in silico. For example in LINE 171, "high predicted FCCs".
Response: Thanks for the nice comment. We have revised the description and pointed out that the FCCs are predicted as suggested.

Line 137. The authors mention, referring to Figure 2d, that there is a phylogenetically based clustering of the clades based on their metabolic models. However, the authors use tSNE to represent these data, which does not present phylogenetic information. The authors should consider how to visually represent the clustering based on the metabolic modeling together with phylogenetic information, such as a phylogenetic tree - can the authors show visualization of real phylogeny vs metabolic modeling clustering in some way?
Response: The reviewer is correct that tSNE does not present phylogenetic information, and the clustering of clade members is merely showing that metabolic networks are more similar within clades. We have modified the text to clarify this. Meanwhile, the reviewer posed us a nice challenge to visualize the metabolic models in a phylogenetic tree.

We have attempted this based on the reaction existence matrix. From this matrix we can calculate the Jaccard distance matrix that characterizes the dissimilarity of two species in terms of reaction existence. From the Jaccard distances, we can draw a phylogenetic tree (see below), but unfortunately this did not perform well. We also tried to remove the core reactions existing in all the species and repeated the analysis, but we still got a similar result. From this we draw the conclusion that the models may be more similar and conserved than gene sequence information, which is not ideal to classify species in high-resolution. In addition, sequence similarity allows for more gradual changes (as indicated by the scores in BLOSUM matrices for sequence alignment), in contrast to the binary absence/presence of reactions from metabolic networks. However, this result will not affect our original conclusion. Regardless, we find that tSNE clustering analysis remains valuable as it visualizes which models are more similar in relation to reaction existence, and our results convincingly show conservation of network structure within clades.

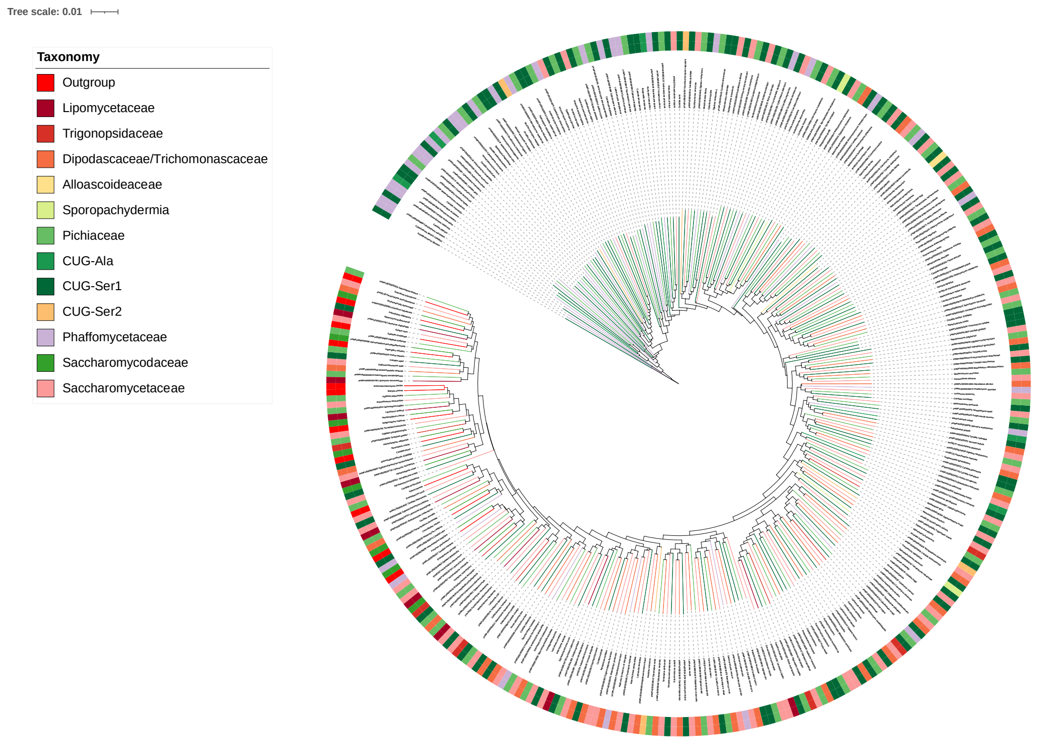

Figure 1 Phylogenetic tree for 343 yeast/fungi species based on the reaction existence matrix from metabolic models

Figure 3b: There appears to be a statistically significant comparison between the dN/dS boxplots for "species number 250-300, accessory metabolic" and "species number 300-343, accessory metabolic". This does not seem to be something that can be appropriately compared, given that the two boxplots fall under different "species number" categories. Furthermore, the observation that the core, more conserved metabolic pathway genes, evolve more slowly and have lower dN/dS, than the accessories pathway genes. This does not seem like a novel observation, but serves to validate the methods applied.
Response: Thanks for the nice comments. Previously, we mainly want to compare the group - “species number 300-343, core metabolic reaction” vs “species number 300-343, accessory metabolic reaction”. We agree that the main conclusion from Figure 3b is not novel. Also, in original Figure 3c, we show that genes from core metabolic pathways are more conserved. Based on your nice comments, we decided to remove the original panel b from Figure 3 to keep it concise.

Line 204-207. The authors mention that a number of genes within PSSs belong to the MAPK signaling pathway. The authors state that this indicates that positive selection could be beneficial to yeast in adapting to environment. This is a dubious claim with no clear evidence. If the authors want to establish a link between the presence of MAPK within PSSs and adaptation, they need to consider alternatives (eg a null hypothesis). For example, are MAPK overrepresented among PSSs, compared to other gene families? Are particular MAPK genes overrepresented among PSSs compared to non-PSSs?
Response: We performed a hypergeometric test for enrichment and found a *P* value of 0.1208 for MAPK pathway genes. Based on this, we removed the claim about the potential relation between presence of MAPK with PSSs and adaptation from the text.

Line 213: same as with Figure 3b, it does not seem very novel that the active and binding sites of enzymes have a lower dN/dS compared to the rest of the enzyme. While the observation that most positively selected or fast-evolved sites are generally >10 angstroms away from active or binding sites and that such sites don't cluster is a little more interesting, these results are still unsurprising. It is expected that such important sites of enzymes are highly conserved. As the authors conclude, these results primarily "confirm" that residues are subject to different pressures depending on function and localization.
Response: While we appreciate that lower dN/dS for active and binding sites would be anticipated, we believe that there is value in our analysis where we assign each residue to 32 functional categories. Interestingly, we also displayed the potential correlation between the evolution of key residues sites and the trait existence in the section of “Evolutionary mechanisms underlying substrate utilization diversity in yeast”.

In the original Figure 4D, actually, we collected the sites annotation from multiple sources. We think this is first time to summarize the evolutionary trend at site level for yeast. In the new version of Figure 4D, we reduced the types of sites for more in-depth analysis (also based on comments from Reviewer 3#). Focusing on the evolutionary trend at these important sites (e.g., Binding sites, Active site or Site, annotated in UniProt), we found that most of these functionally important sites are extremely conserved as 2440 out of 3370 residues are kept the same across species in 1364 proteins with reference one from *S. cerevisiae*. For example, all 5 functionally important sites (H9, R60, E87, K98, H182) of phosphoglycerate mutase 1 (YKL152C) are the same across all yeast species. Also, we could find divergent evolution at some important sites, for example, for the putative 6-phosphofructo-2-kinase (YLR345W), the active site at D173 in 23 of 337 species from *Metschnikowia* genus (these 23 species actually are close with each other in the phylogenetic tree according to[10]) was substituted in by glutamic acid, while the original and alternative residues are both belong to ionic amino acids. We have updated Figure 4 and the main text based on this new analysis. The content in original Figure 4D is reduced and the full description can be found in Appendix Figure 5. We also added a new Figure 4E to illustrate in-depth analysis about the evolution of these key residue sites.

Figure 4c, it will be interesting/important to compare the exact same categories here as in Figure 3c. Both investigate similar concepts, but for some reason categories are left out of 4C/extra categories are included in 3C.
Response: A very good idea. We have updated both Figure 3c and Figure 4c based on your suggestion.

Line 242, also relevant to Figure 5: there is clear variability in HGT between clades. Could the authors discuss this? What explains this variability? Most clades have almost zero HGT, while a few have visibly more.
Response: Indeed, there is clear variability in HGT across clades, and while we cannot fully explain this variability, we have now included a discussion on this observation. One thing to consider is that Figure 5A is only numerating the HGT events that are related to enzymes involved in metabolic trait diversity, so non-enzymatic HGT events are not included. Nonetheless, interestingly clades with higher levels of HGT, i.e., Lipomycetaceae, Trigonopsidaceae and Dipodascaceae/Trichomonascaceae, are closely related to the

Wickerhamiella/Starmerella (W/S) clade that has shown to have acquired large numbers of genes from bacteria[11]. Why the W/S clade has been so permittable to HGT is however unknown, although it might be related with the large majority of W/S yeasts being ecologically associated with floricolous insects and flowers. Natural habitats might therefore also be behind our observed higher levels of HGT in selected clades (Fig 5A)

Contrastingly, low or absent HGT in selected clades might be confounded by alterations in the genetic code, such as the CUG triplet coding for serine or alanine instead of leucine[12] that would act as a barrier to HGT.

Line 274 - when discussing ethanol utilization in Hansensiaspora, the authors state that their prediction is in good agreement with experimentally determined substrate utilization in these species. However, there is no reference. Could the authors identify a reference and expand on this statement?
Response: The ability to grow on glycerol but not citrate, succinate and ethanol was experimentally shown in the collected phenotype data for all 332 yeast species (Dataset EV1), wherethe ethanol data in specific is from[13]. We have clarified this in the manuscript.

Line 300, Figure 6. URA1 has been duplicated many times in the outgroup of fungi, but only one is Crabtree positive. How do the authors explain this in light of their claim that URA1 is important for emergence of Crabtree effect?
Response: About the link between URA1 and Crabtree effect has previously been stated that "one could still speculate that the time of origin of the short-term Crabtree effect and the time of origin of the long-term Crabtree effect seem to be very close to each other and may even overlap, coinciding with the horizontal transfer of URA1 and the ability to proliferate anaerobically"[14]. While we do observe some correlation between URA1 HGT and emergence of the Crabtree effect, it is not fully determinative as the reviewer highlighted. Moreover, the role of URA1 in emergence of the Crabtree effect is entirely unclear to the best of our knowledge. Therefore, and in an effort to improve the focus in the manuscript, we have decided to no longer present these results. The text and Fig 6 have been modified accordingly.

Line 305-309: The authors mention that there was positive selection for genes in the EMP pathway among four independent lineages of Crabtree-positive yeasts. To establish the significance of this positive selection, the authors need to compare to a null hypothesis. Does this positive selection also occur in the outgroup of non-Crabtree fungi? Does it occur at all in non-Crabtree yeasts? The presence alone of positive selection does not prove involvement of these genes in the Crabtree effect.
Response: We performed a null hypothesis analysis, where we randomly selected 25 species from the 76 experimentally confirmed Crabtree negative species as the foreground (or test)branches in the "branch-site" model analysis. We found that only a relatively small ratio of species (24-33%) in the null hypothesis analysis showed positive selection in the four tested genes, while in our original two independent calculations, the three genes from EMP pathways belonged to the top selected genes occurred in over 85% of tested species distributed in the four major clades containing Crabtree-positive species while one gene in oxidative phosphorylation (ATP1) showed positive selection occurred in over 55% of test

species. It is obvious some genes only show positive selection in part of Crabtree positive yeast species from less than four major clades. To reduce bias, we mainly focus on those three genes from EMP pathways (they could be regarded as top selected genes among all the tested). Considering the result from the null hypothesis analysis, it is feasible that some Crabtree negative species that are evolutionarily close to Crabtree positive species can also exhibit some positive selection in these interesting genes without the Crabtree effect emerging. We have included the results of this analysis in Dataset EV7 and while the procedure was added to the Method section.

As for the non-Crabtree fungi, we have two problems with performing further analysis. Firstly, the fungi as out-group are evolutionarily far away from other Crabtree positive species (especially from major clades of *Saccharomycetaceae*) based on the phylogeny tree from[10]. The null hypothesis evidence from non-Crabtree fungi may not well support the current evolution analysis in which mainly the Crabtree positive yeast species as test group and Crabtree negative yeast species as reference group. The second and main issue is that for the 11 fungi selected as out-group used in this work, except for *S. pombe*, we do not collect enough evidence to determine whether they are Crabtree positive or negative from literature data, hindering further analysis.

In the revised paper, we put Crabtree effect as the first example to testify our pipeline and we removed the previous claim about the contribution of these three genes to the Crabtree effect except indicating their potential correlation. As it has recently been reported that increased PYK activity through one missense mutant shifts *S. pombe* from respiration towards fermentation[15] while attenuation of PYK activity was important for *S. cerevisiae* to acquire a Crabtree-negative phenotype[16], we thus think that the sequence evolution of these three genes from the EMP pathway is likely relevant for the Crabtree effect while the detailed causality still needs more in-depth molecular experiment. We also added some discussion at the end of our manuscript according to the suggestion from Reviewer 3#.

Line 311-315. The authors invoke circumstantial evidence about FBA1, PGK and PYK, to explain the evolution of the Crabtree effect across clades. This lacks experimental evidence. The authors should consider removing these claims or providing experimental evidence. At the very least, similar to above, does this positive selection also occur in the outgroup of non-Crabtree fungi? Does it occur at all in non-Crabtree yeasts?
Response: Yes. It is indeed difficult to make the conclusion about whether the gene selection results in the emergence of the Crabtree effect or the Crabtree effect could affect the gene selection (also mentioned by Reviewer 3#). But it is likely these two events are intertwined with each other as other multiple evolutionary events (like gene duplication, HGT) are all relevant to the Crabtree effect. So here we mainly rephased our description in the result and discussion to make the conclusion more reasonable and accurate. Although some experimental data supported that the protein (or gene) sequence divergence could strengthen or weaken Crabtree effect[7,16], it will need more in-depth analysis to further test how gene position selection affects the evolution of Crabtree effect, especially the emergence of Crabtree effect, which we think it will need the efforts from our yeast community. As suggested by reviewer, we removed additional claims both in the result and discussion to make the conclusion more concise.
We also added more calculation based on your nice suggestion (see above).

Line 333-337: The authors describe that some positively selected genes were upregulated in previous experiments in Baker's yeast. However, just because 42 of positively selected genes (related to thermotolerance) were upregulated at 38C vs 30C in yeast does not mean that they contribute to thermotolerance; if the authors want to use this argument, they also need to consider how many of not positively selected genes were upregulated at 38C. These positively selected proteins could reflect a general stress response rather than actually contributing directly to the phenotype. The evidence presented in lines 338-348 is more intriguing, although it also presents circumstantial evidence without experimental verification.
Response: This is a good point. Appendix Figure 9C shows that more genes were upregulated under higher temperature, which were not among the positive selected genes in our analysis. We agree with the idea that upregulation of positively selected genes did not mean they could contribute to thermotolerance. Here we just display how the expression of these selected genes changed under 38C vs 30C for the model organism – *S. cerevisiae*. Besides the evidence from model simulation, we found a recent paper that verifies that the genes we found could help to increase the fitness of growth under higher temperature. More details are provided in response to the following comment.

Overall, regarding the discussion of the Crabtree effect and Thermotolerance, many claims are made about the factors that may contribute to these phenotypes across yeasts. However, the claims about organismal biology are not supported by the data of the current study. The authors present an intriguing overview of the distribution of these phenotypes across yeasts, and also present a survey of interesting candidate genes/traits that correlate with these phenotypes. Yet, given the results of the study, it still remains unknown whether the methods presented by the authors can truly predict the evolution of complex metabolic traits. If the authors want to make claims about mechanisms and involvement of specific players, more experimental validation is needed. For example, regarding thermotolerance, in Figure 6, the authors could experimentally validate prediction of thermotolerance in the disulfide isomerase presented as a specific example (does this protein have altered Tm if the residues of interest are changed?). Scaling back the claims in this section would not compromise the novelty or contribution of the overall study. In fact, given the vast amount of data and the large scope of the study, the authors may be best served scaling back (or relegating to the SI) the data/discussion of Figure 6, which would provide a more succinct and well-supported message to the reader.
Response: Thanks for your nice suggestion! We now added a new experimental result that support our findings. "More importantly, a recent experiment by transferring the genes from a thermotolerant yeast species - *Ogataea polymorpha* to *S. cerevisiae* verified that numerous genes (about 60 gene were tested) can contribute to thermotolerance of yeast and these genes are significantly enriched in GO-term of "translation" and "ribosome"[17], partially consistent with the result here." Actually 6 positive selected genes can be verified by this study even only a limited of genes were tested. Accordingly, we added the discussion as "However, to date, the systematic experimental evidences are still lack to testify the mechanical correlation between the top positively selected genes (or unique mutations) and the growth fitness under higher temperature. Thus, it is anticipated that a similar gene transfer experiment between yeasts with distinct fitness can be designed for these top

positively selected gene in future studies to further evaluate the polygenetic and/or pathway-level evolutionary mechanism for the emergence of thermotolerance in yeast."

While considering multiple factors affecting the evolution of complex traits, i.e., the Crabtree effect and thermotolerance, we reduced the content in Figure 6. In the new Figure 6, we now focus more on the results from gene positive selection analysis and metabolic model prediction.

A general comment: many figure captions lack information, making the figures difficult to understand. The authors should provide more experimental information and explanation for figures throughout the paper. We provide examples below with the understanding that the authors should look the entire paper and consider changes:
Response: Thanks for your suggestion. We now refined the figures and legend throughout the whole paper based on the following detailed comments.

Figure 1a caption needs more information/explanation as it is unclear what various panels represent. This is especially important as this Figure sets up the methods and concepts used throughout the paper.
Response: We have extensively redesigned Figure 1A and reformulated its caption to improve clarity.

Supplementary Figure 2C - how do the authors determine the cause of inconsistencies? How do they determine promiscuous enzymes?
Response: In case a model was able to catabolize a substrate in contrast to the experimental evidence, we first identified in the model which reactions and enzymes were responsible for the degradation of the particular substrate. If one of the enzymes involved was also annotated to other metabolic reactions in the model (i.e., promiscuous enzyme), then enzyme promiscuity was tallied as responsible for the false positive. How did this inconsistency then arise? Inclusion of reactions in ssGEMs are foremost guided by the presence of enzymes associated to those reactions. If a particular enzyme is promiscuous in pan-GEM (i.e., associated to multiple reactions with different substrates), then all these associated reactions will end up in ssGEMs that contain the enzyme. However, substrate selectivity might have changed during evolution of selected strains, such that the enzyme in specific strains might have lost or never gained the promiscuous activity that are otherwise assigned to it in pan-GEM, which likely results in inconsistence between the prediction and measured traits. We have now clarified this in the text and figure captions.

Supplementary Figure 3a. Unclear what abbreviations mean. This comment is relevant throughout figures where abbreviations for species names are used.
Response: We have added notes for those abbreviations in the figure legend and also for other figures (Figure 2, Figure 3E, Appendix Figure 4B).

Figure 2e - what are the horizontal labels on the heatmap?
Response: The x-axis indicates proteins, while the y-axis indicates the strain-specific ecGEMs. We have clarified this in the figure caption.

Figure 3b - why are species grouped in the following way by number? The analysis is unclear. The significance of the statistical test of the species grouping is unclear.
Response: In the original Figure 3B, the number in the x-axis represents the groups of OGs classified by number of species with ortholog gene from this OG. The high number means the OGs are more conserved across yeast species. The significance analysis is used to evaluate how the types of reactions (core or accessory) and the conservation of OG affect the gene-level evolution rate. It should be noted that with your kind comments in above, we decided to remove Figure 3B, which make the full figure more concise.

Figure 4c. Box plot is unclear. Unclear from caption what points and scale represent.
Response: In the original right boxplot from Figure 4C, each circle represents one of the genes with positively selected sites and the values on in the y-axis is are the corresponding number of species containing each gene. We think this boxplot increases complexity for readers to understand this Figure, thus we removed the boxplot while updated the Figure 4C based on your nice comments above.

Figure 6g and 6h (it should be 5g and 5h?). It is completely unclear what these figures represent and their relevance to the current study.
Response: We have thoroughly edited the figure captions.

Reviewer #3:

In this study, Lu and coworkers study the evolution and divergence of yeast metabolic networks. In short, single-species metabolic networks, as well as one pan-genomic network are generated and analyzed. Perhaps not surprisingly, the results show strong conservation of core metabolism and active sites, and more evolutionary divergence for the peripheral networks and peripheral residues, with promiscuous enzymes playing a central role in evolutionary innovation. The strength of this study then perhaps does not lie in the generation of novel evolutionary concepts, but rather it providing an unprecedented bird's eye view on yeast metabolic networks and their evolutionary divergence. As such, it is a very interesting piece of work that provides a great overview and which will serve as a resource for future research.

Overall, the study is solid and the text is very well written and clear. My main remark is that some evolutionary claims are perhaps not fully supported by data. As such, I only have a few relatively minor comments and suggestions to add some nuances.

1.A lot of attention is given to dN/dS analysis to find signs of positive and negative selection. However, this is a rather rough estimate of selection that has several limitations. It would be good to evaluate the results more critically. In particular, I wonder whether many of the higher dN/dS figures found for peripheral residues are reflecting a lack of negative (purifying) selection, rather than true positive selection? In that sense, focusing on residues that are not neutral and bound to have an effect (such as those located in an active site) and looking for smaller deviations in dN/dS could provide more valuable insight; and/or finding examples of parallel evolution. In addition, it would be good to dive a bit deeper into some examples, and to be a bit more cautious with drawing overly strong conclusions (eg lines 379).

Response: To find the signature of positive selection in codon (residue site) level, we used two different procedures (FEL[18] and FUBAR[19]) to do the calculation. These procedures gave divergent results (Figure 4B) and to reduce false positives, only genes (862) with positive selected sites identified by all procedures were further analyzed at the pathway level (Figure 4C).

Nonetheless, the reviewer is right that residue sites with higher dN/dS (e.g., >2) may reflect a lack of negative selection, rather than true positive selection. In our work, positive selection was selected based on a strict cut-off defined in FUBAR and FEL, for example, in FURAR, a residue site with dN/dS >1 and with posterior probabilities either larger than 0.9 [19] can be regarded as positively selected. With this approach the false positives will be filtered out, but some true positive cases would not be identified.

To further explore the relation between the residue site function and its evolution, we further analyzed the evolution rate for the functionally important sites, e.g., active sites and binding sites, recorded in *S. cerevisiae* for 1364 proteins. It shows that the dN/dS for all these sites are in ranges of 0-0.66, signifying that purifying selection plays a main role in these functionally important sites. Such a tendency could verify that the function at the residue site level could largely constrain the evolution across yeast species. We also found that most of these functionally important sites are extremely conserved as 2440 of 3370 residues sites are kept same across species. For example, all 5 sites (H9, R60, E87, K98, H182) of Phosphoglycerate mutase 1 (YKL152C) are the same across all yeast species. Also, we could find divergent evolution (but not parallel evolution, the number of parallel evolution should be rare for these important sites after our initial manual check) at some important sites, for example, for the putative 6-phosphofructo-2-kinase (YLR345W), the active site at D173 in 23 of 337 species mainly from *Metschnikowia* genus (these 23 species actually are close with each other in the phylogenetic tree according to [10]) was substituted in by glutamic acid, while both the original and alternative residues belong to ionic amino acids. We have updated Figure 4 and the main text based on this new analysis.

For the original sentence in line 379, we changed it as “These findings demonstrate that though the evolution is multi-dimensional and dynamic, the metabolic functions reflected by ssGEMs and protein 3D structures potentially shape the evolution at both gene and amino acid residue level.”

2. Some aspects are perhaps a bit underdeveloped, such as for example the role of promiscuous enzymes. Is it possible to provide more in-depth analyses there? The archetypal evolutionary model is that evolutionary innovation happens through duplication and subsequent divergence of promiscuous enzymes, allowing a side-activity to develop further and become much higher in one of the paralogs. For example, the residues that provide specificity or promiscuity in maltose-degrading enzymes have been relatively well studied (and there must be more examples), and this information could be mapped onto the networks to link it to copy numbers, residues and phenotypes.
Response: Following the reviewer’s suggestion we have connected the evolution of key residues with trait loss in maltose utilization by looking into maltase in our models. It showed that genes related to maltose degradation belong to ortholog group of OG1179. We added more analysis in the main txt as the following:

The duplications of promiscuous enzymes followed by divergent sequence evolution are frequently observed in yeast. As one typical example, maltase is able to degrade maltose, turanose, maltotriose and sucrose[20]. We found that the responsible gene had at least one duplication in 77 yeast species, among which 74 could utilize maltose and 69 utilize sucrose. With the enzyme functional annotation from *S. cerevisiae* S288c, it showed that three key residue sites (active site: D214, E276; site: D349) of maltase are highly conserved in each duplication, maintaining the same in about 96% of all ortholog gene members. In some species, divergent sequence evolution at key residue sites can be still observed. For instance, in *Nadsonia fulvescens var. elongata*, one of these key residue sites was mutated in one duplication (from D349 to E349) while remaining unchanged in another duplication. Such a divergent sequence evolution in promiscuous enzyme may determine the specificity of enzyme in catalyzing various substrates. More interestingly, in 6 yeast species with only one duplication of maltase, gaps were found at these three key residue sites, coincidently with the trait loss in maltose utilization in these species.

3. Whereas I do see the correlation between selection for certain EMP genes and emergence of the Crabtree effect; but I am less convinced that you can write that the emergence of the Crabtree effect benefitted from selection on the genes; it seems difficult to establish a direct causal relationship here (did the flux first change, stimulating the Crabtree effect, or did the Crabtree effect evolve and then cause more positive selecton on the EMP, or -perhaps most likely- are both events simply intertwined, likely also with many other events? I think that this is also the theory of the authors, but some of the sentences (eg line 406-407) miss that nuance.
Response: We agree with the comments and note that the other reviewers had also indicated their reservations about the causation. Accordingly, we extensively rephased the results and discussion in relation to the Crabtree effect.

4. I must admit being confused about the proposed link between the EMP and temperature tolerance. While the link between the EMP and the Crabtree effect is also not crystal clear (see above), the link is quite believable and obvious. As far as I can judge, this is less the case for EMP and temperature tolerance; so I would again advise to either provide more evidence (which could entail making mutations in some of the enzymes and studying their stability and activity at various temperatures; as well as studying the effect of expressing combinations of alleles on the temperature tolerance of a given yeast species), or soften the claims a bit.
Response: We have now removed the description about the potential correlation between EMP and temperature tolerance. Instead, we have included evidence that supports our evolution analysis in revealing the potential mechanism of thermotolerance in yeast. "More importantly, a recent experiment by transferring the genes from a thermotolerant yeast species - *Ogataea polymorpha* to *S. cerevisiae* verified that numerous genes (about 60 gene were tested) can contribute to thermotolerance of yeast and these genes are significantly enriched in GO-term of "translation" and "ribosome"[17], partially consistent with the result here." Six positive selected genes can be verified by this study even only a limited number of genes were tested. Accordingly, we added the discussion as "However, to date, systematic experimental evidence is still lacking to testify the mechanical correlation between the top positively selected genes (or unique mutations) and growth fitness under higher

temperature. Thus, a similar gene transfer experiment between yeasts with distinct fitness can be designed for these top positively selected gene in future studies to further testify the polygenetic and/or pathway-level evolutionary mechanism for the emergence of thermotolerance in yeast."

But we did not find evidences about the effect of unique mutations on the temperature tolerance. Considering that multiple genes and mutations render the heat tolerance for a given yeast species, here we soften the discussion about the potential effects of listing two mutations on the heat-tolerance based on your kind suggestion. The related figures are transferred to supplementary figures. We think the amounts of unique mutations in heat-tolerant yeast species will serve as good resources to be further tested by the yeast community. We added one comment in description of the content in the revised version: "However, the functionalities of these mutations and their correlations with thermotolerance need more in-depth molecular studies."

Typo line 414 („)
Response: Fixed.

best wishes-
Kevin Verstrepen

Reference

1. Price, M.N., Dehal, P.S. & Arkin, A.P. FastTree 2--approximately maximum-likelihood trees for large alignments. *PLoS One* **5**, e9490 (2010).
2. Nguyen, L.-T., Schmidt, H.A., Von Haeseler, A. & Minh, B.Q. IQ-TREE: a fast and effective stochastic algorithm for estimating maximum-likelihood phylogenies. *Molecular biology and evolution* **32**, 268-274 (2015).
3. Minh, B.Q., Nguyen, M.A.T. & von Haeseler, A. Ultrafast approximation for phylogenetic bootstrap. *Molecular biology and evolution* **30**, 1188-1195 (2013).
4. Opulente, D.A. et al. Factors driving metabolic diversity in the budding yeast subphylum. *BMC Biol* **16**, 26 (2018).
5. Nishimura, K. et al. Identification of enzyme responsible for erythritol utilization and reaction product in yeast *Lipomyces starkeyi*. *Journal of Bioscience and Bioengineering* **101**, 303-308 (2006).
6. Carly, F. et al. Identification and characterization of EYD1, encoding an erythritol dehydrogenase in *Yarrowia lipolytica* and its application to bioconvert erythritol into erythrulose. *Bioresource Technology* **247**, 963-969 (2018).
7. Linder, T. A genomic survey of nitrogen assimilation pathways in budding yeasts (sub-phylum *Saccharomycotina*). *Yeast* **36**, 259-273 (2019).
8. Pearson, W.R. An Introduction to Sequence Similarity ("Homology") Searching. *Current Protocols in Bioinformatics* **42**, 3.1.1-3.1.8 (2013).
9. Juergens, H. et al. Contribution of Complex I NADH Dehydrogenase to Respiratory Energy Coupling in Glucose-Grown Cultures of *Ogataea parapolymorpha*. *Appl Environ Microbiol* **86** (2020).
10. Shen, X.X. et al. Tempo and Mode of Genome Evolution in the Budding Yeast Subphylum. *Cell* **175**, 1-13 (2018).

11. Goncalves, P., Goncalves, C., Brito, P.H. & Sampaio, J.P. The *Wickerhamiella/Starmerella* clade-A treasure trove for the study of the evolution of yeast metabolism. *Yeast* **37**, 313-320 (2020).
12. Richards, T.A., Leonard, G., Soanes, D.M. & Talbot, N.J. Gene transfer into the fungi. *Fungal Biology Reviews* **25**, 98-110 (2011).
13. Kurtzman, C.P., Fell, J.W. & Boekhout, T. The Yeasts: A Taxonomic Study (Elsevier Science). (2011).
14. Dashko, S., Zhou, N., Compagno, C. & Piskur, J. Why, when, and how did yeast evolve alcoholic fermentation? *FEMS Yeast Res* **14**, 826-832 (2014).
15. Kamrad, S. et al. Pyruvate kinase variant of fission yeast tunes carbon metabolism, cell regulation, growth and stress resistance. *Mol Syst Biol* **16**, e9270-e9270 (2020).
16. Yu, T. et al. Reprogramming Yeast Metabolism from Alcoholic Fermentation to Lipogenesis. *Cell* **174**, 1549-1558 e1514 (2018).
17. Seike, T., Narazaki, Y., Kaneko, Y., Shimizu, H. & Matsuda, F. Random Transfer of *Ogataea polymorpha* Genes into *Saccharomyces cerevisiae* Reveals a Complex Background of Heat Tolerance. *J Fungi (Basel)* **7** (2021).
18. Kosakovsky Pond, S.L. & Frost, S.D.W. Not So Different After All: A Comparison of Methods for Detecting Amino Acid Sites Under Selection. *Mol Biol Evol* **22**, 1208-1222 (2005).
19. Murrell, B. et al. FUBAR: a fast, unconstrained bayesian approximation for inferring selection. *Mol Biol Evol* **30**, 1196-1205 (2013).
20. Brown, C.A., Murray, A.W. & Verstrepen, K.J. Rapid expansion and functional divergence of subtelomeric gene families in yeasts. *Current biology : CB* **20**, 895-903 (2010).

Thank you for sending us your revised manuscript. We have now heard back from the two reviewers who were asked to evaluate your revised study. As you will see below, the reviewers are satisfied with the performed revisions and are supportive of publication. Reviewer #2 lists a couple of minor points, which we would ask you to address in a revision.

Before we formally accept the study for publication, we would also ask you to address some remaining editorial issues listed below.

REFEREE REPORTS

Reviewer #2:

We appreciate the authors careful consideration of our suggested revisions and their efforts to incorporate them into the paper. Our general comments about novelty, overgeneralization, and lack of clarity have been addressed and we are excited for this contribution to the field. A few minor comments:

Line 58: These studies demonstrate that the evolution from gene to pathways all to assure that the strains have the flexibility to gain new capabilities under specific niches.

Line 62: reword "to which extent" to "to what extent"

Line 63: sentence restructuring suggested, and perhaps worth considering cutting out some content or splitting into two sentences, for easier reading. For example, "Also, besides gene duplication and enzyme promiscuity, there has not been a systematic investigation in yeast for how to connect multiple evolutionary events and characterize general evolutionary trends under the emergence of different metabolic traits."

Line 79: "combining" instead of combing?

Line 331: replace "coincidently" with "coinciding"?

Reviewer #3:

I was already a fan of the original manuscript. With this revised version, the authors responded to all the concerns I raised, and I am therefore happy to support publication.
kevin

REFEREE REPORTS

Reviewer #2:

We appreciate the authors careful consideration of our suggested revisions and their efforts to incorporate them into the paper. Our general comments about novelty, overgeneralization, and lack of clarity have been addressed and we are excited for this contribution to the field. A few minor comments:
Response: We thanks for all the nice inputs and suggestions from the reviewer. We have revised paper based on the following comments.

Line 58: These studies demonstrate that the evolution from gene to pathways all to assure that the strains have the flexibility to gain new capabilities under specific niches. Response: We have revised the sentence as the following-"These studies demonstrate that the evolution from gene to pathways all potentially assure that the strains have the flexibility to gain new capabilities under specific niches"

Line 62: reword "to which extent" to "to what extent"
Response: Fixed it.

Line 63: sentence restructuring suggested, and perhaps worth considering cutting out some content or splitting into two sentences, for easier reading. For example, "Also, besides gene duplication and enzyme promiscuity, there has not been a systematic investigation in yeast for how to connect multiple evolutionary events and characterize general evolutionary trends under the emergence of different metabolic traits."
Response: We have rephrased this sentence accordingly.

Line 79: "combining" instead of combing?
Response: Fixed it. We are sorry for this error.

Line 331: replace "coincidently" with "coinciding"?
Response: we think "coincidently" is correct here.

Reviewer #3:

I was already a fan of the original manuscript. With this revised version, the authors responded to all the concerns I raised, and I am therefore happy to support publication.
Kevin

Response: We thanks for all the nice inputs and suggestions from the reviewer.

ACCEPTED **4th Oct 2021**

Thank you again for sending us your revised manuscript. We are now satisfied with the modifications made and I am pleased to inform you that your paper has been accepted for publication.

EMBO PRESS

YOU MUST COMPLETE ALL CELLS WITH A PINK BACKGROUND ➔

PLEASE NOTE THAT THIS CHECKLIST WILL BE PUBLISHED ALONGSIDE YOUR PAPER

Corresponding Author Name: Jens Nielsen

Journal Submitted to: Molecular Systems Biology

Manuscript Number: MSB-2021-10427

USEFUL LINKS FOR COMPLETING THIS FORM

http://www.antibodypedia.com
http://1degreebio.org
http://www.equator-network.org/reporting-guidelines/improving-bioscience-research-repor
http://grants.nih.gov/grants/olaw/olaw.htm
http://www.mrc.ac.uk/Ourresearch/Ethicsresearchguidance/Useofanimals/index.htm
http://ClinicalTrials.gov
http://www.consort-statement.org
http://www.consort-statement.org/checklists/view/32-consort/66-title
http://www.equator-network.org/reporting-guidelines/reporting-recommendations-for-tumo
http://datadryad.org
http://figshare.com
http://www.ncbi.nlm.nih.gov/gap
http://www.ebi.ac.uk/ega
http://biomodels.net/
http://biomodels.net/miriam/
http://jjj.biochem.sun.ac.za
https://osp.od.nih.gov/biosafety-biosecurity-and-emerging-biotechnology/
http://www.selectagents.gov/

Reporting Checklist For Life Sciences Articles (Rev. June 2017)

This checklist is used to ensure good reporting standards and to improve the reproducibility of published results. These guidelines are consistent with the Principles and Guidelines for Reporting Preclinical Research issued by the NIH in 2014. Please follow the journal's authorship guidelines in preparing your manuscript.

A- Figures
1. Data
The data shown in figures should satisfy the following conditions:

- the data were obtained and processed according to the field's best practice and are presented to reflect the results of the experiments in an accurate and unbiased manner.
- figure panels include only data points, measurements or observations that can be compared to each other in a scientifically meaningful way.
- graphs include clearly labeled error bars for independent experiments and sample sizes. Unless justified, error bars should not be shown for technical replicates.
- if n< 5, the individual data points from each experiment should be plotted and any statistical test employed should be justified
- Source Data should be included to report the data underlying graphs. Please follow the guidelines set out in the author ship guidelines on Data Presentation.

2. Captions

Each figure caption should contain the following information, for each panel where they are relevant:

- a specification of the experimental system investigated (eg cell line, species name).
- the assay(s) and method(s) used to carry out the reported observations and measurements
- an explicit mention of the biological and chemical entity(ies) that are being measured.
- an explicit mention of the biological and chemical entity(ies) that are altered/varied/perturbed in a controlled manner.

- the exact sample size (n) for each experimental group/condition, given as a number, not a range;
- a description of the sample collection allowing the reader to understand whether the samples represent technical or biological replicates (including how many animals, litters, cultures, etc.).
- a statement of how many times the experiment shown was independently replicated in the laboratory.
- definitions of statistical methods and measures:
  - common tests, such as t-test (please specify whether paired vs. unpaired), simple χ2 tests, Wilcoxon and Mann-Whitney tests, can be unambiguously identified by name only, but more complex techniques should be described in the methods section;
  - are tests one-sided or two-sided?
  - are there adjustments for multiple comparisons?
  - exact statistical test results, e.g., P values = x but not P values < x;
  - definition of 'center values' as median or average;
  - definition of error bars as s.d. or s.e.m.

Any descriptions too long for the figure legend should be included in the methods section and/or with the source data.

In the pink boxes below, please ensure that the answers to the following questions are reported in the manuscript itself. Every question should be answered. If the question is not relevant to your research, please write NA (non applicable). We encourage you to include a specific subsection in the methods section for statistics, reagents, animal models and human subjects.

B- Statistics and general methods

Please fill out these boxes ➔ (Do not worry if you cannot see all your text once you press return)

1.a. How was the sample size chosen to ensure adequate power to detect a pre-specified effect size?	In all the related analysis, sample size in one group is larger than 3 for the statistical test analysis.
1.b. For animal studies, include a statement about sample size estimate even if no statistical methods were used.	No animal studies.
2. Describe inclusion/exclusion criteria if samples or animals were excluded from the analysis. Were the criteria pre-established?	For each gene ortholog group, if the number of species under ortholog group is smaller than 6, then the related ortholog group was not used for further analysis.
3. Were any steps taken to minimize the effects of subjective bias when allocating animals/samples to treatment (e.g. randomization procedure)? If yes, please describe.	No. We have no related experiments.
For animal studies, include a statement about randomization even if no randomization was used.	No animal studies.
4.a. Were any steps taken to minimize the effects of subjective bias during group allocation or/and when assessing results (e.g. blinding of the investigator)? If yes please describe.	No.
4.b. For animal studies, include a statement about blinding even if no blinding was done	No animal studies.
5. For every figure, are statistical tests justified as appropriate?	Yes.
Do the data meet the assumptions of the tests (e.g., normal distribution)? Describe any methods used to assess it.	Yes. For two group comparisons in this work, a two tailed Wilcoxon rank sum test was used. In general, the data could meet the assumptions of this two tailed Wilcoxon rank sum test: 1) The two samples are independent of one another; 2) The two populations have equal variance or spread
Is there an estimate of variation within each group of data?	Yes.

Is the variance similar between the groups that are being statistically compared?	Yes. But in some cases, variance may exist between groups.

C- Reagents

6. To show that antibodies were profiled for use in the system under study (assay and species), provide a citation, catalog number and/or clone number, supplementary information or reference to an antibody validation profile. e.g., Antibodypedia (see link list at top right), 1DegreeBio (see link list at top right).	Antibodies were not used.
7. Identify the source of cell lines and report if they were recently authenticated (e.g., by STR profiling) and tested for mycoplasma contamination.	Cell lines were not used.

* for all hyperlinks, please see the table at the top right of the document

D- Animal Models

8. Report species, strain, gender, age of animals and genetic modification status where applicable. Please detail housing and husbandry conditions and the source of animals.	No animal models were used.
9. For experiments involving live vertebrates, include a statement of compliance with ethical regulations and identify the committee(s) approving the experiments.	No animal models were used.
10. We recommend consulting the ARRIVE guidelines (see link list at top right) (PLoS Biol. 8(6), e1000412, 2010) to ensure that other relevant aspects of animal studies are adequately reported. See author guidelines, under 'Reporting Guidelines'. See also: NIH (see link list at top right) and MRC (see link list at top right) recommendations. Please confirm compliance.	No animal models were used.

E- Human Subjects

11. Identify the committee(s) approving the study protocol.	No human subjects are related.
12. Include a statement confirming that informed consent was obtained from all subjects and that the experiments conformed to the principles set out in the WMA Declaration of Helsinki and the Department of Health and Human Services Belmont Report.	No human subjects are related.
13. For publication of patient photos, include a statement confirming that consent to publish was obtained.	No human subjects are related.
14. Report any restrictions on the availability (and/or on the use) of human data or samples.	No human subjects are related.
15. Report the clinical trial registration number (at ClinicalTrials.gov or equivalent), where applicable.	No human subjects are related.
16. For phase II and III randomized controlled trials, please refer to the CONSORT flow diagram (see link list at top right) and submit the CONSORT checklist (see link list at top right) with your submission. See author guidelines, under 'Reporting Guidelines'. Please confirm you have submitted this list.	No human subjects are related.
17. For tumor marker prognostic studies, we recommend that you follow the REMARK reporting guidelines (see link list at top right). See author guidelines, under 'Reporting Guidelines'. Please confirm you have followed these guidelines.	No human subjects are related.

F- Data Accessibility

18: Provide a "Data Availability" section at the end of the Materials & Methods, listing the accession codes for data generated in this study and deposited in a public database (e.g. RNA-Seq data: Gene Expression Omnibus GSE39462, Proteomics data: PRIDE PXD000208 etc.) Please refer to our author guidelines for 'Data Deposition'.<br><br>Data deposition in a public repository is mandatory for:<br>a. Protein, DNA and RNA sequences<br>b. Macromolecular structures<br>c. Crystallographic data for small molecules<br>d. Functional genomics data<br>e. Proteomics and molecular interactions	Yes. We have prepared for it.
19. Deposition is strongly recommended for any datasets that are central and integral to the study; please consider the journal's data policy. If no structured public repository exists for a given data type, we encourage the provision of datasets in the manuscript as a Supplementary Document (see author guidelines under 'Expanded View' or in unstructured repositories such as Dryad (see link list at top right) or Figshare (see link list at top right).	Yes. We have prepared for it.
20. Access to human clinical and genomic datasets should be provided with as few restrictions as possible while respecting ethical obligations to the patients and relevant medical and legal issues. If practically possible and compatible with the individual consent agreement used in the study, such data should be deposited in one of the major public access-controlled repositories such as dbGAP (see link list at top right) or EGA (see link list at top right).	We have no this type of data.
21. Computational models that are central and integral to a study should be shared without restrictions and provided in a machine-readable form. The relevant accession numbers or links should be provided. When possible, standardized format (SBML, CellML) should be used instead of scripts (e.g. MATLAB). Authors are strongly encouraged to follow the MIRIAM guidelines (see link list at top right) and deposit their model in a public database such as Biomodels (see link list at top right) or JWS Online (see link list at top right). If computer source code is provided with the paper, it should be deposited in a public repository or included in supplementary information.	We have prepared models in various format, like .mat and .sbml. All the models can be found in https://github.com/SysBioChalmers/Yeast-Species-GEMs. Due to the huge file size of the mat models, we put models in .mat and .sbml format in https://figshare.com/articles/dataset/Comprehensive_evolution_analysis_with_genome_scale_metabolic_models_reveals_diverse_mechanisms_in_metabolic_innovations_across_332_yeast_species/14473776. Additionally, as editor suggested we are in the process of having all models available via Biomodels. Once the paper is published, we will update the citation information in Biomodels database accordingly.

G- Dual use research of concern

22. Could your study fall under dual use research restrictions? Please check biosecurity documents (see link list at top right) and list of select agents and toxins (APHIS/CDC) (see link list at top right). According to our biosecurity guidelines, provide a statement only if it could.	No.
